# Supplementary material for: Synthesis and Anti-Angiogenic Activity of Novel c(RGDyK) Peptide-Based JH-VII-139-1 Conjugates
Source: Pharmaceutics. 2023 Jan 22;15(2):381. doi: 10.3390/pharmaceutics15020381 (PMC9962512; doi:10.3390/pharmaceutics15020381)
Supplement: Supplementary file 1 [file pharmaceutics-15-00381-s001.zip › pharmaceutics-2148996-supplementary.pdf]

Supplementary Material

Synthesis and Anti-Angiogenic Activity of Novel c(RGDyK) Peptide-Based JH-VII-139-1 Conjugates

George Leonidis [1], Anastasia Koukiali [1], Ioanna Sigala [1], Katerina Tsimaratou [2], Dimitris Beis [2], Thomas Giannakouros [1], Eleni Nikolakaki [1] and Vasiliki Sarli [1,*]

[1] Department of Chemistry, Aristotle University of Thessaloniki, University Campus, 54124 Thessaloniki, Greece
[2] Zebrafish Disease Model Laboratory, Biomedical Research Foundation Academy of Athens, 11527 Athens, Greece
* Correspondence: sarli@chem.auth.gr

Supporting Info

LC/ESI-MS, HPLC parameters and method development	S3
^{1}H-NMR and ^{13}C-NMR spectra for 2	S5
LC/ESI-MS analysis of 2	S6
^{1}H-NMR and ^{13}C-NMR spectra for 3	S7
^{1}H-NMR and ^{13}C-NMR spectra for 5	S8
^{1}H-NMR and ^{13}C-NMR spectra for 6a	S9
LC/ESI-MS analysis of 6a	S10
^{1}H-NMR and ^{13}C-NMR spectra for 6b	S11
^{1}H-NMR and ^{13}C-NMR spectra for 7	S12
LC/ESI-MS analysis of 7	S13
^{1}H-NMR and ^{13}C-NMR spectra for 8	S14
LC/ESI-MS analysis of 8	S15
^{1}H-NMR and ^{13}C-NMR spectra for 9	S16
LC/ESI-MS analysis of 9	S17
^{1}H-NMR and ^{13}C-NMR spectra for 10	S18
LC/ESI-MS analysis of 10	S19
^{1}H-NMR spectra for 12 and 13	S20
^{1}H-NMR and ^{13}C-NMR spectra for JH-VII-139-1	S21
ESI-HRMS analysis of JH-VII-139-1	S22
^{1}H-NMR spectrum for 16	S23
^{1}H-NMR and ^{13}C-NMR spectra for 17	S24

**LC/ESI-MS analysis of 17**	**S25**
**^{1}H-NMR and ^{13}C-NMR spectra for geo75**	**S26**
**LC/ESI-MS analysis of geo75**	**S27**
**^{1}H-NMR and ^{13}C-NMR spectra for 20**	**S28**
**LC/ESI-MS analysis for 20**	**S29**
**^{1}H-NMR and ^{13}C-NMR spectra for geo77**	**S30**
**LC/ESI-MS analysis of geo77**	**S31**
**^{1}H-NMR and ^{13}C-NMR spectra for 22**	**S32**
**^{1}H-NMR and ^{13}C-NMR spectra for 23**	**S33**
**ESI-MS analysis of 23**	**S34**
**^{1}H-NMR and ^{13}C-NMR spectra for geo85**	**S35**
**LC/ESI-MS analysis of geo85**	**S36**
**^{1}H-NMR and ^{13}C-NMR spectra for 24**	**S37**
**LC/ESI-MS analysis of 24**	**S38**
**^{1}H-NMR and ^{13}C-NMR spectra for geo105**	**S39**
**LC/ESI-MS analysis of geo105**	**S40**
**^{1}H-NMR and ^{13}C-NMR spectra for geo107**	**S41**
**LC/ESI-MS analysis of geo107**	**S42**
**^{1}H-NMR spectra for 28 and 29**	**S43**
**LC/ESI-MS analysis of 29**	**S44**
**^{1}H-NMR spectra for geo106**	**S45**
**LC/ESI-MS analysis of 106**	**S46**
**Chemostability Studies**	**S47**
**In vitro kinase inhibition assay**	**S48**

LC/ESI-MS, HPLC parameters and method development

LC-MS/HPLC: LC-20AD Shimadzu connected to Shimadzu LCMS-2010EV

HPLC column: Supelco discovery C18, 5μm 250 x 4.6mm

Flow rate: 0.4 mL/min

Column temperature: 26°C

UV detector: 254, 280 nm

MS detector: 1.65kV

- Method 1

gradient elution 90% H_2O – 10% ACN (+0.1% formic acid) to 5% H_2O - 95% ACN (+0.1% formic acid)

Table S1. Conditions for Method 1.

time (min)	H_2O (% v/v conc.)	ACN (% v/v conc.)
3	90	10
22	15	85
25	10	90
29	10	90
31	5	95
37	5	95

- Method 2

gradient elution 90% H_2O – 10% ACN (+0.1% formic acid) to 10% H_2O - 90% ACN (+0.1% formic acid)

Table S2. Conditions for Method 2.

time (min)	H_2O (% v/v conc.)	ACN (% v/v conc.)
3	90	10
22	15	85
25	10	90
26	10	90

- Method 3

gradient elution 90% H_2O – 10% ACN (+0.1% formic acid) to 10% H_2O - 90% ACN (+0.1% formic acid)

Table S3. Conditions for Method 3.

time (min)	H_2O (% v/v conc.)	ACN (% v/v conc.)
3	90	10
22	15	85
25	10	90

29	10	90

- Method 4

gradient elution 90% H_2O – 10% ACN (+0.1% formic acid) to 5% H_2O - 95% ACN (+0.1% formic acid)

Table S4. Conditions for Method 4.

time (min)	H_2O (% v/v conc.)	ACN (% v/v conc.)
3	90	10
22	15	85
25	10	90
29	10	90
31	5	95
33	5	95

- Method 5

gradient elution 90% H_2O – 10% ACN (+0.1% formic acid) to 10% H_2O - 90% ACN (+0.1% formic acid)

Table S5. Conditions for Method 5.

time (min)	H_2O (% v/v conc.)	ACN (% v/v conc.)
3	90	10
22	15	85
25	10	90

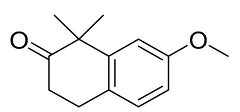

2

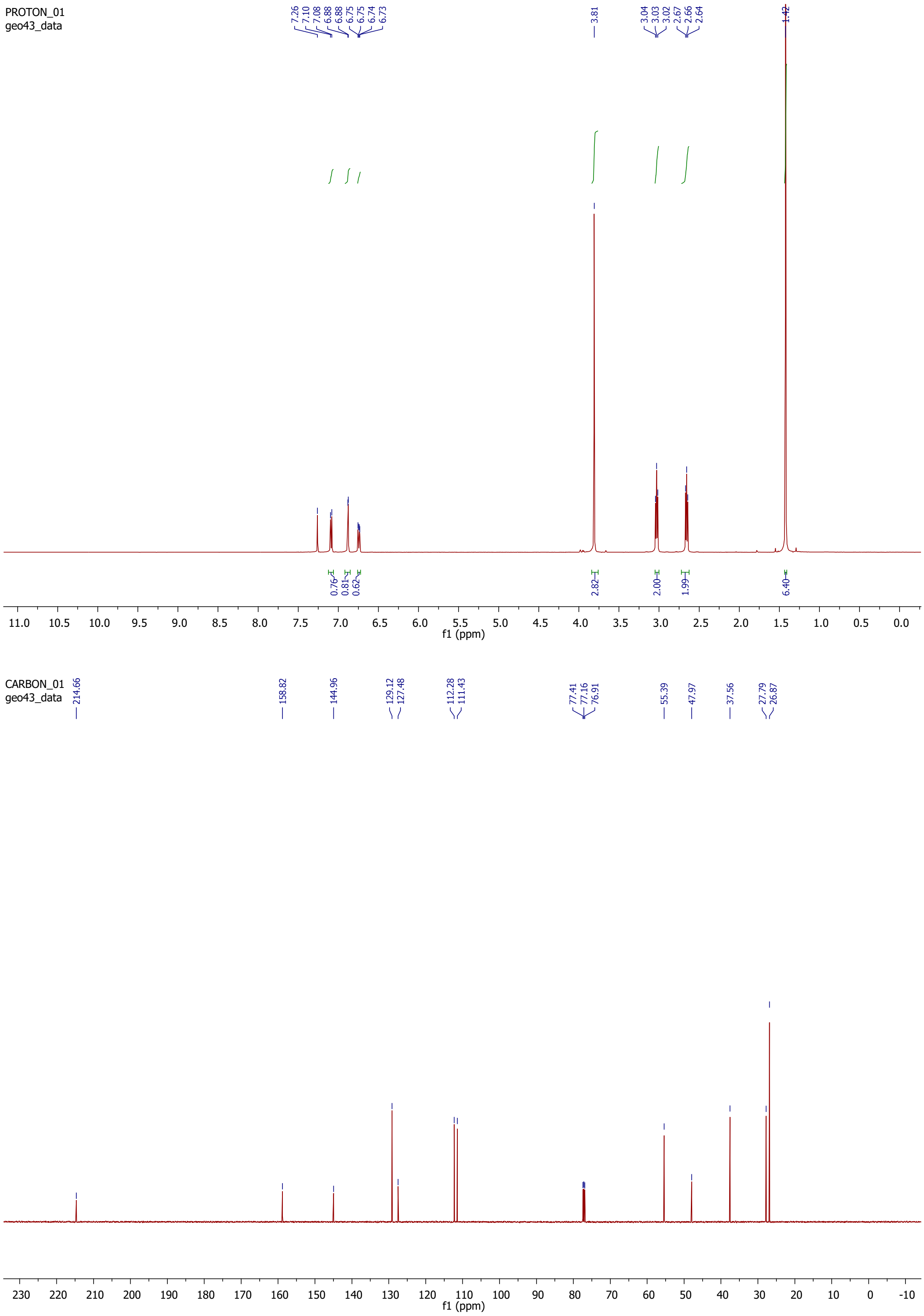

Figure S1. ^{1}H-NMR and ^{13}C-NMR spectra for compound **2**

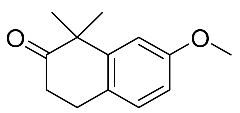

2

MeOH isocratic elution

Retention time: 8.6 m

area: 1536963 (absorbance units x minutes)

total area: 1622159 (absorbance units x minutes)

area %: (1536963/1622159)*100 = 94.7 %

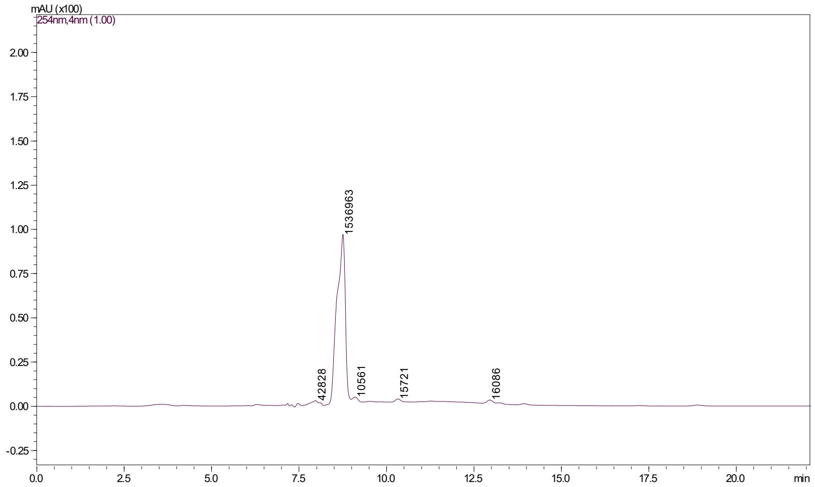

Figure S2. ESI-LCMS of compound **2** after column chromatography purification.

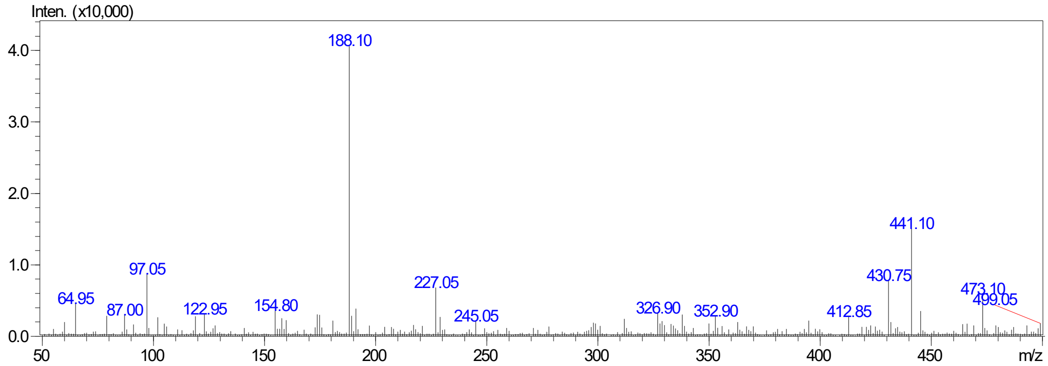

Figure S3. ESI-MS for **2**, positive mode: m/z calcd mass for $C_{13}H_{16}O_2Na$ $[M+Na]^+$=227.10, was found 227.05

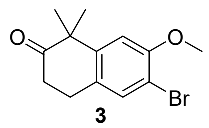

3

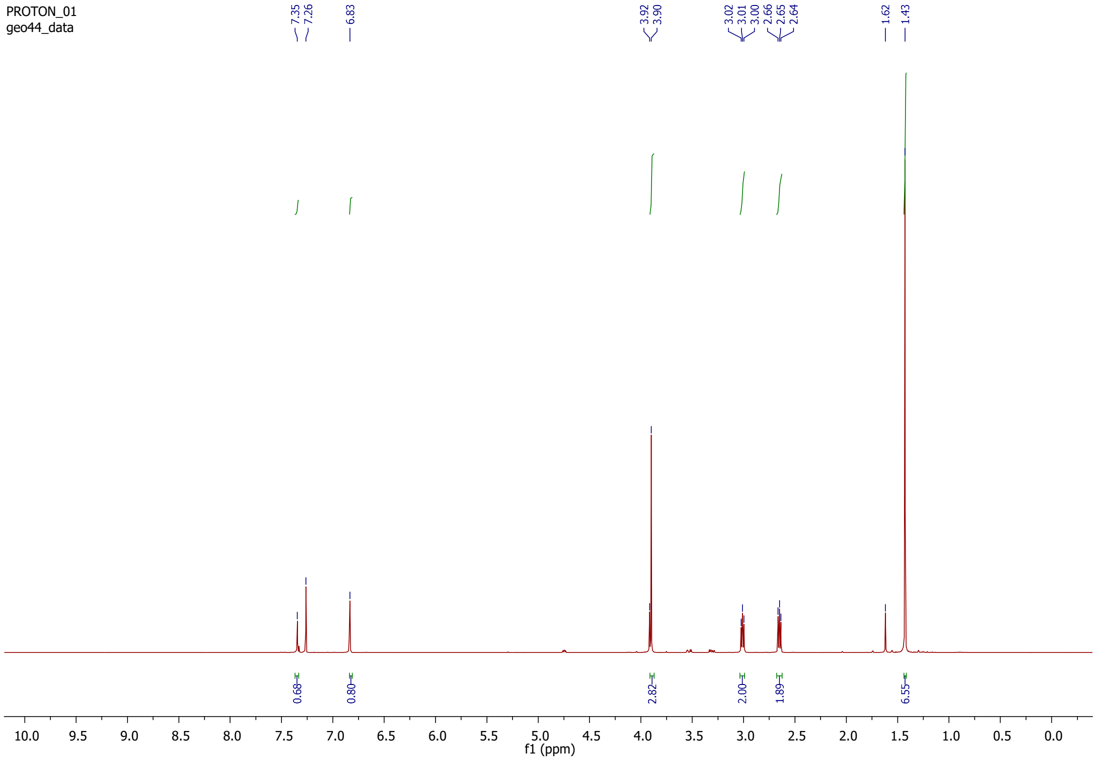

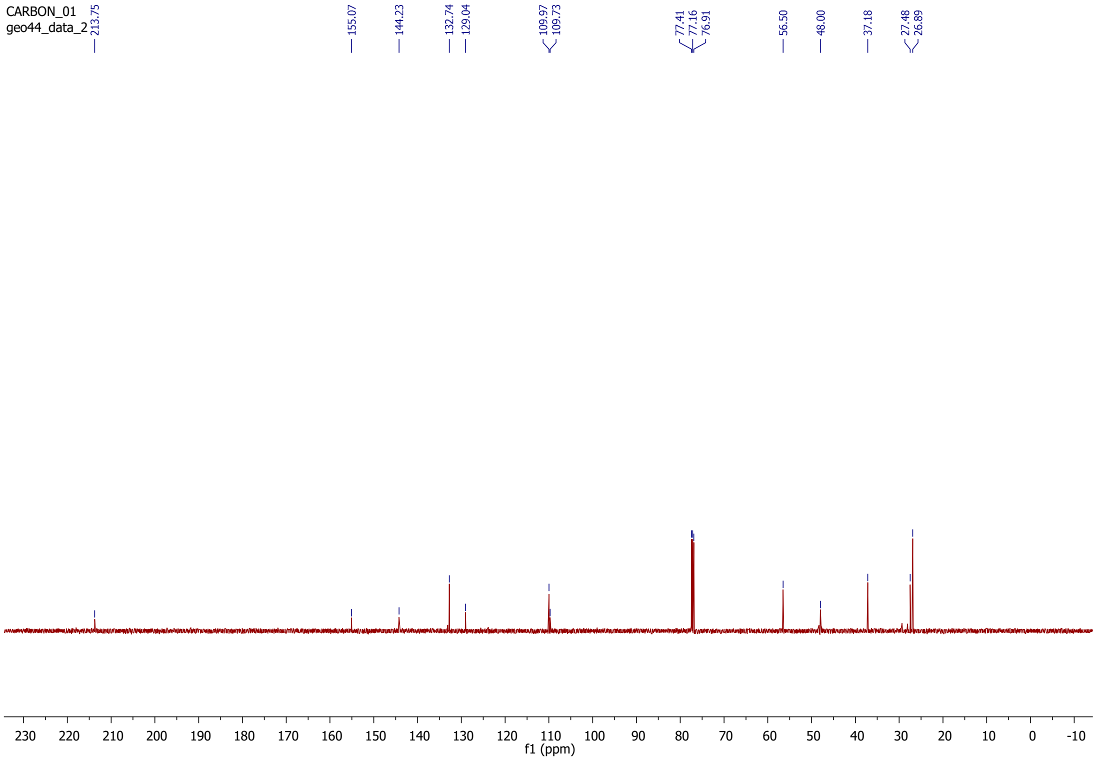

Figure S4. ^{1}H-NMR and ^{13}C-NMR spectra for compound **3**

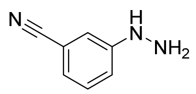

5

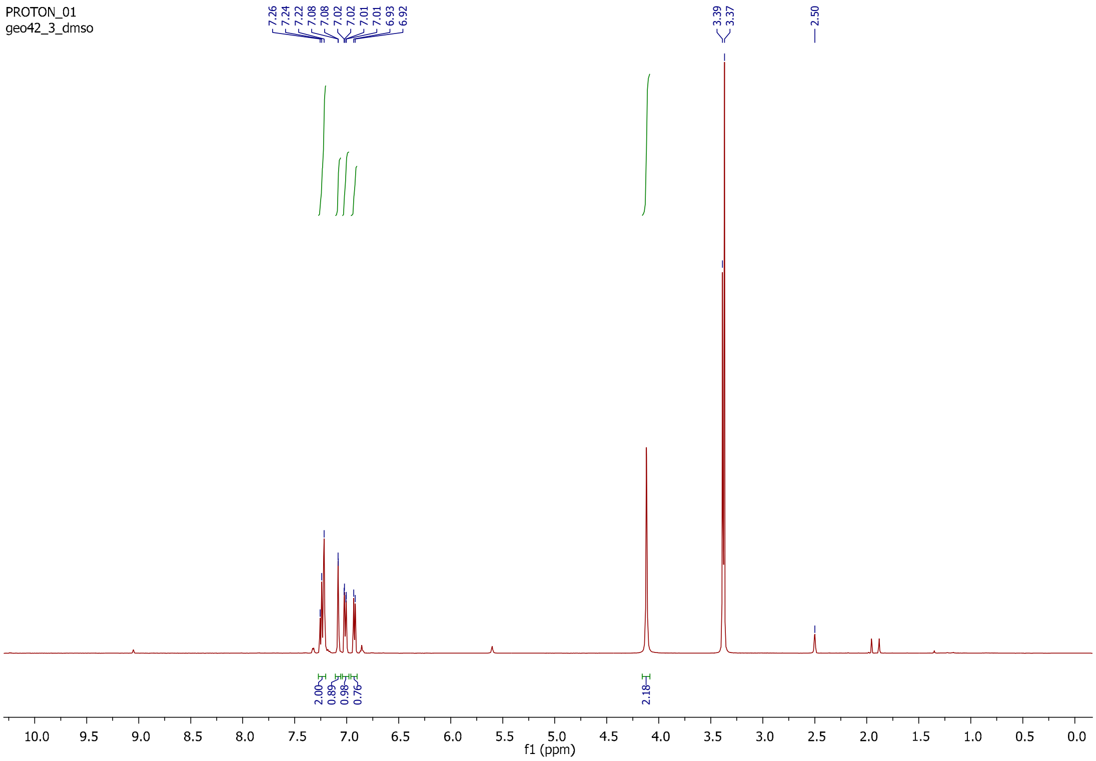

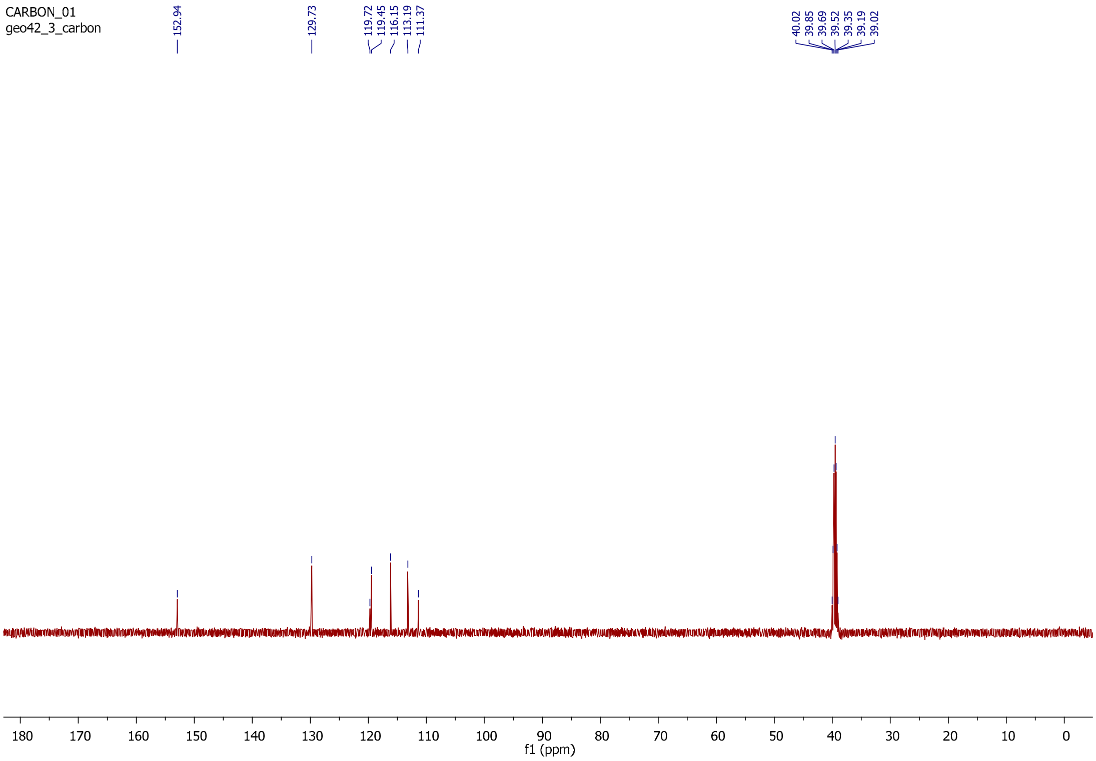

Figure S5. ^{1}H-NMR and ^{13}C-NMR spectra for compound **5**

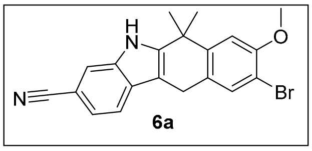

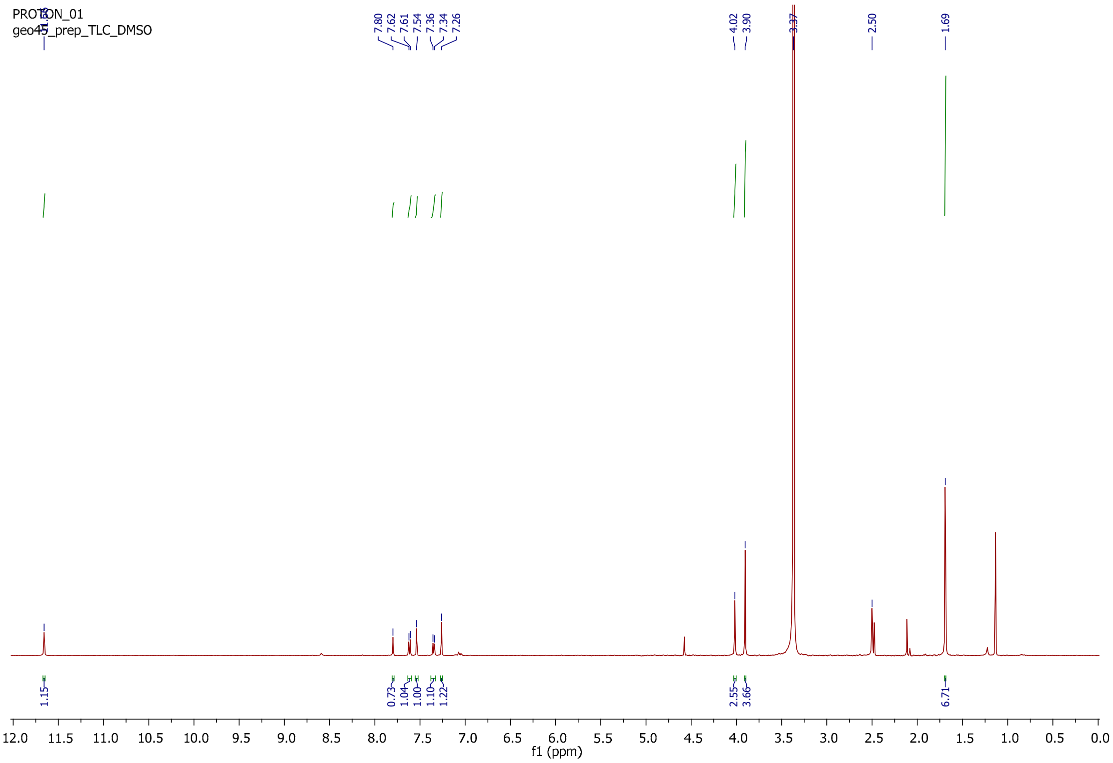

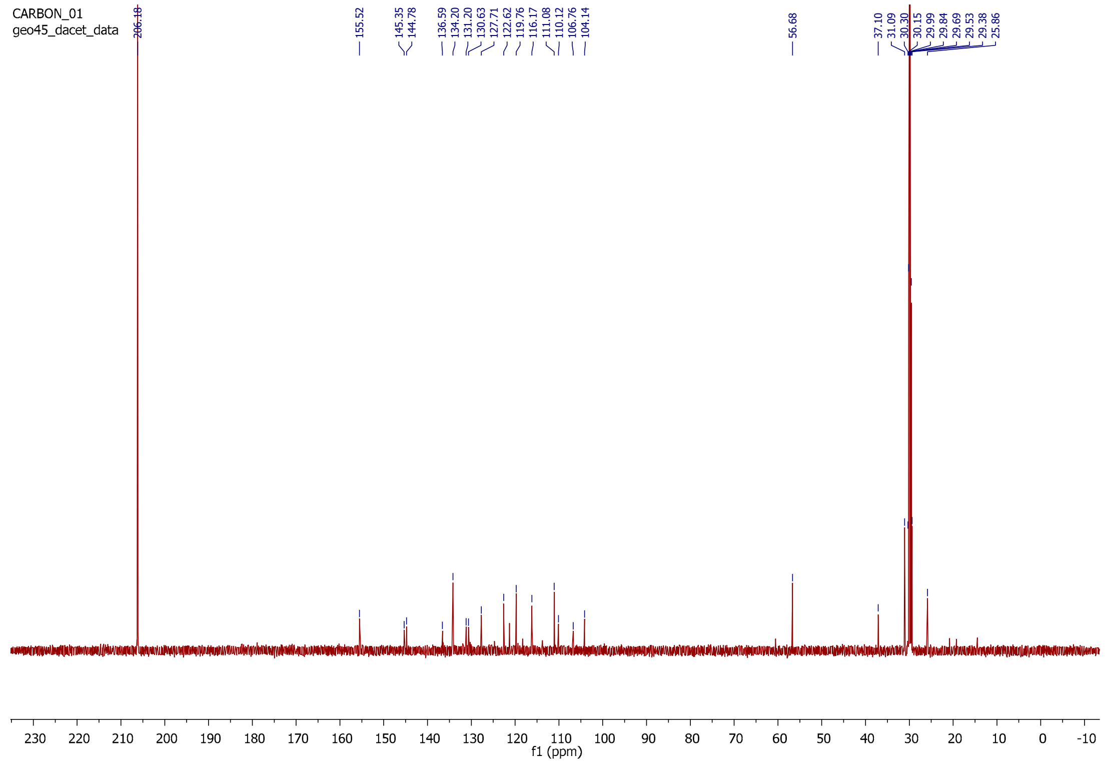

Figure S6. ^{1}H-NMR and ^{13}C-NMR spectra for compound **6a**

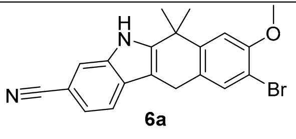

Method 1

Retention time: 32.8 m

area: 41951699 (absorbance units x minutes)

total area: 45555041 (absorbance units x minutes)

area %: (41951699/45555041)*100 = 92.1 %

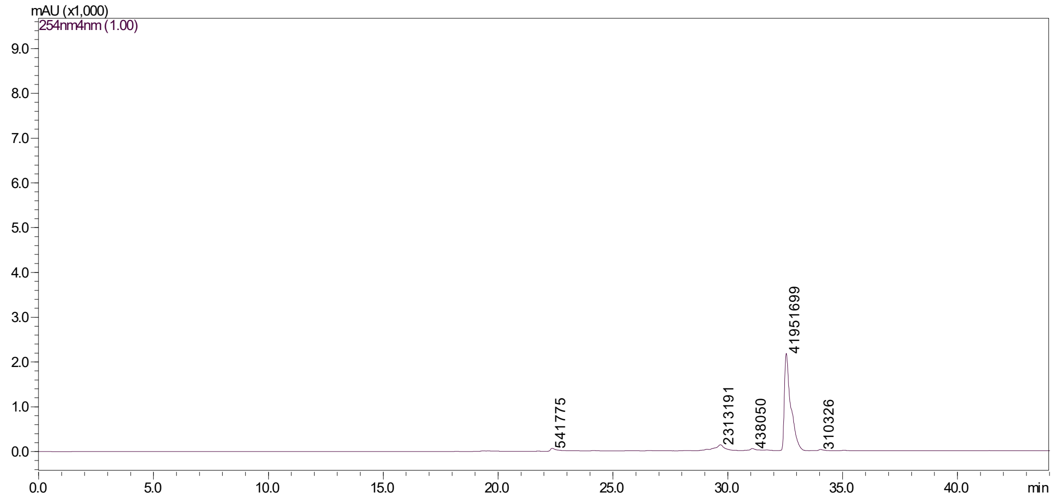

Figure S7. ESI-LCMS of compound **6a** after column chromatography purification

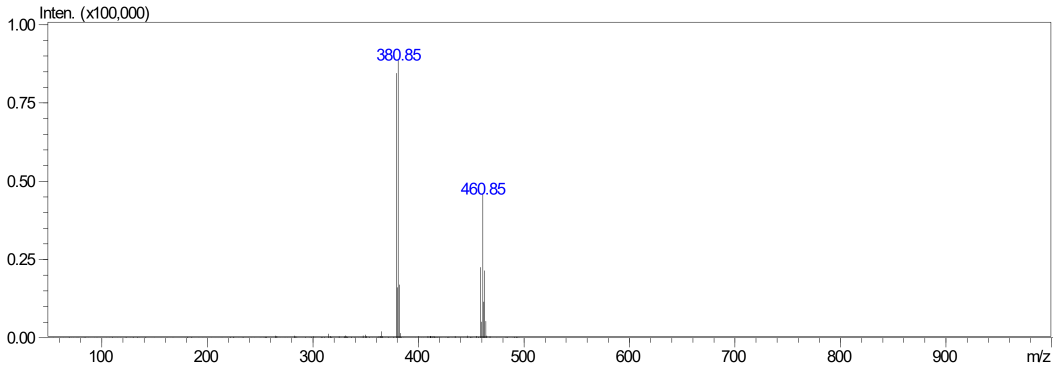

Figure S8. ESI-MS for **6a**, negative mode: m/z calcd mass for $C_{20}H_{16}BrN_2O$ $[M-H]^-$=379.04, was found 380.85

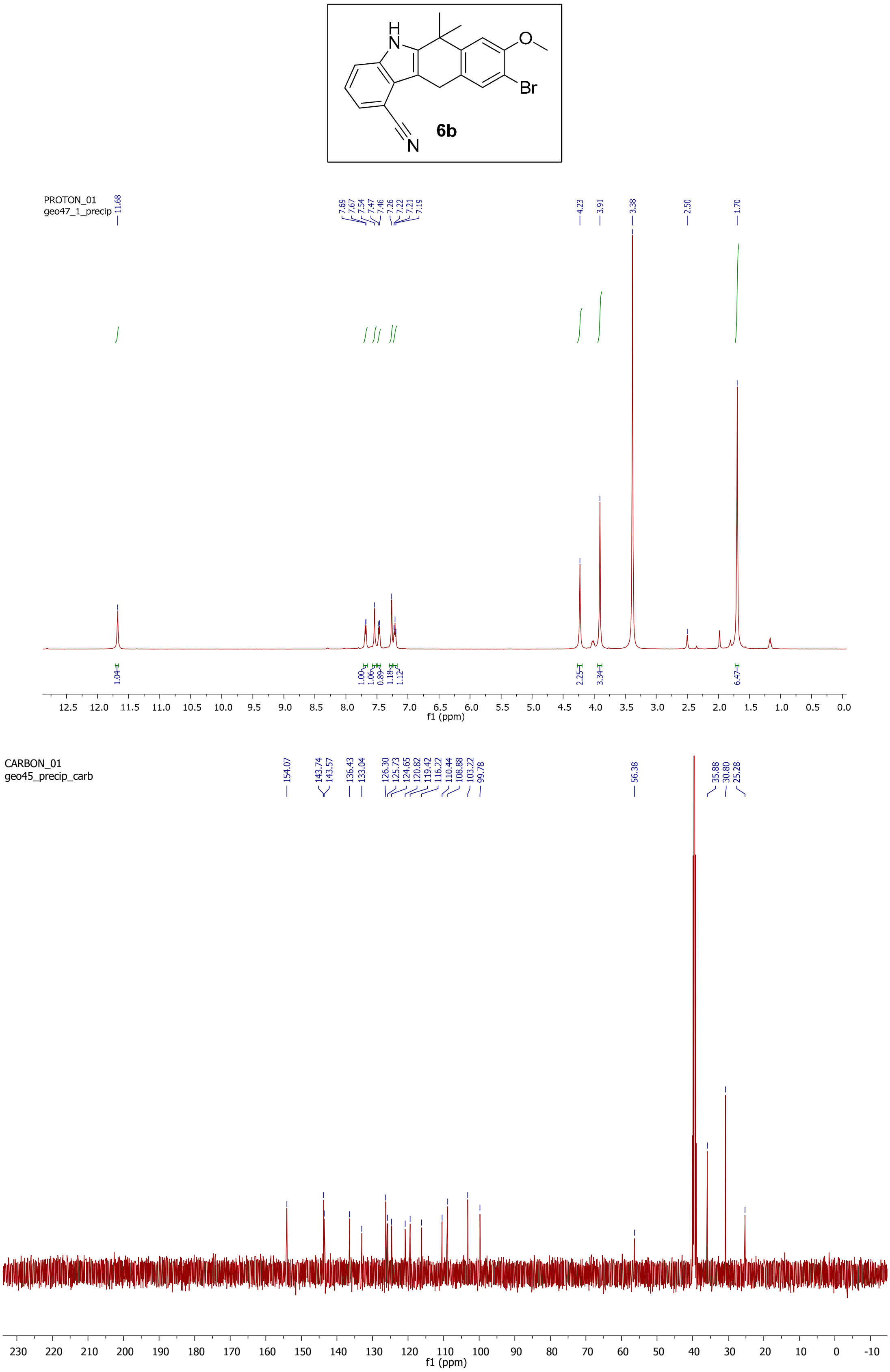

Figure S9. ^{1}H-NMR and ^{13}C-NMR spectra for compound **6b**

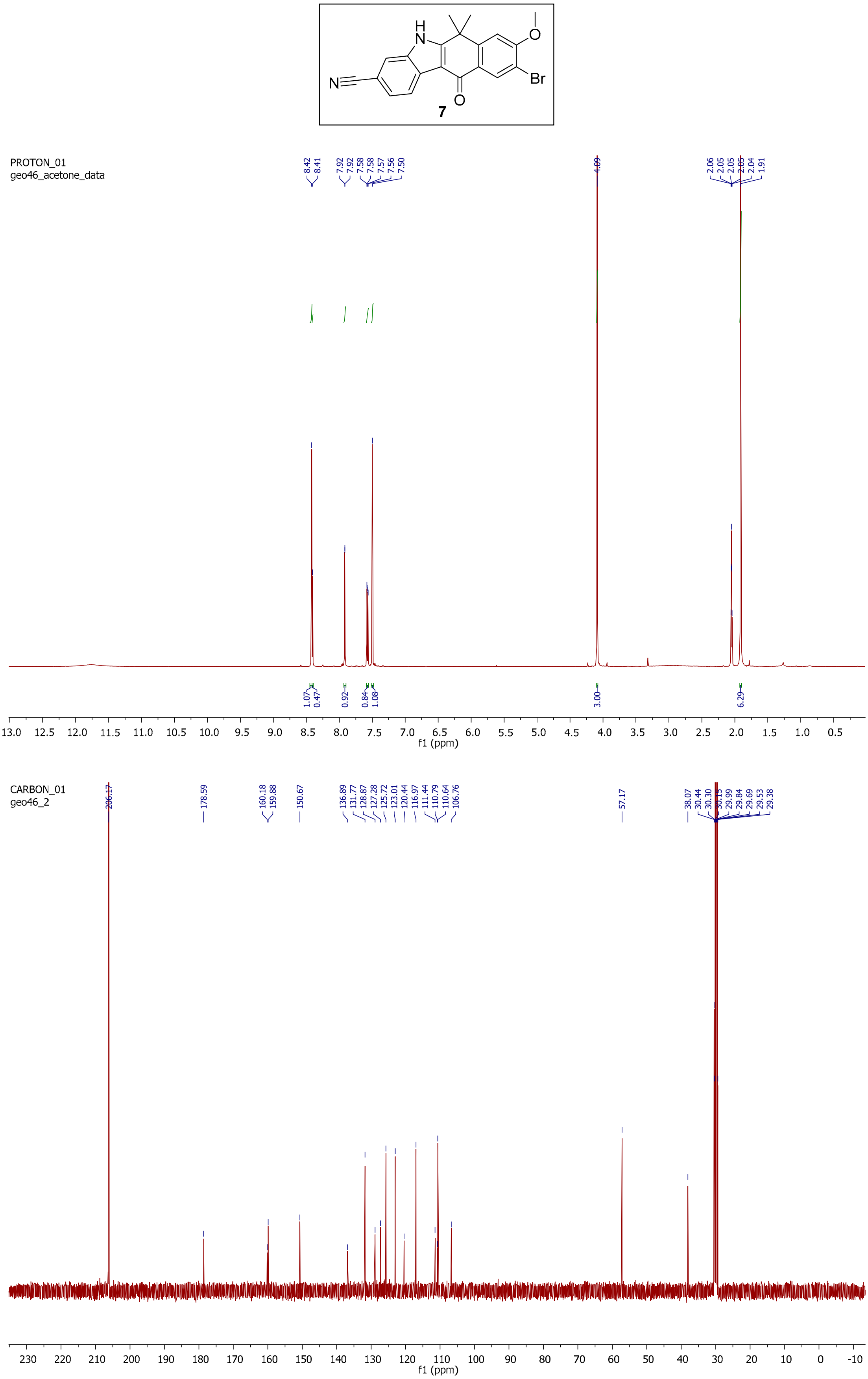

Figure S10. ^{1}H-NMR and ^{13}C-NMR spectra for compound **7**

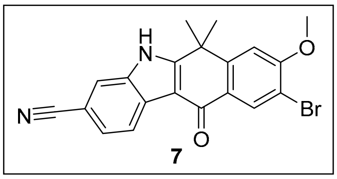

Method 1

Retention time: 29.1 m

area: 80446192 (absorbance units x minutes)

total area: 80745691 (absorbance units x minutes)

area %: (80446192/80745691)*100 = 99.6%

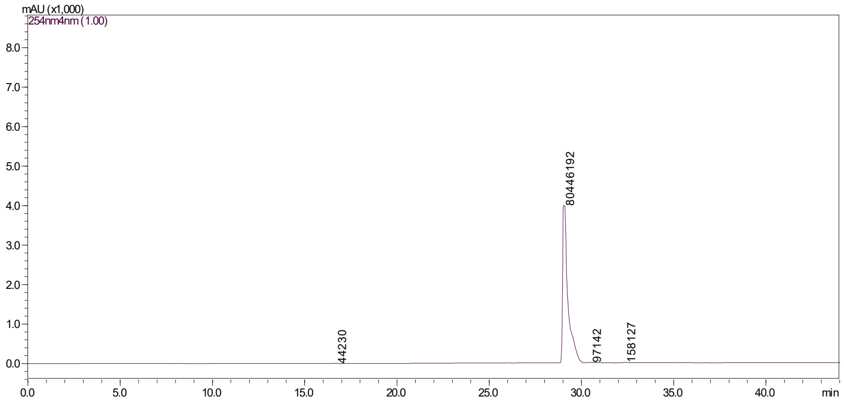

Figure S11. ESI-LCMS of compound **7** after column chromatography purification

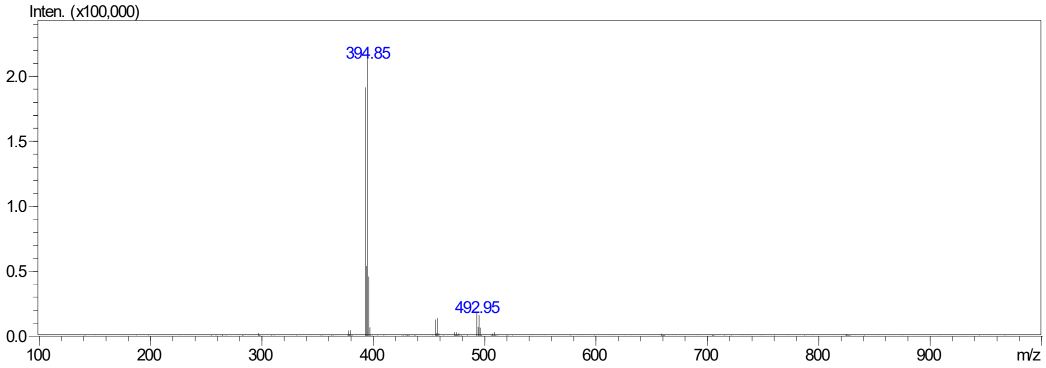

Figure S12. ESI-MS for **7**, negative mode: m/z calcd mass for $C_{20}H_{14}BrN_2O_2$ $[M-H]^-$=393.02, was found 394.85

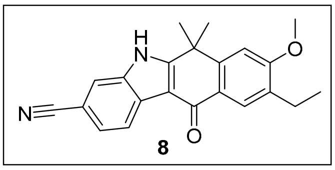

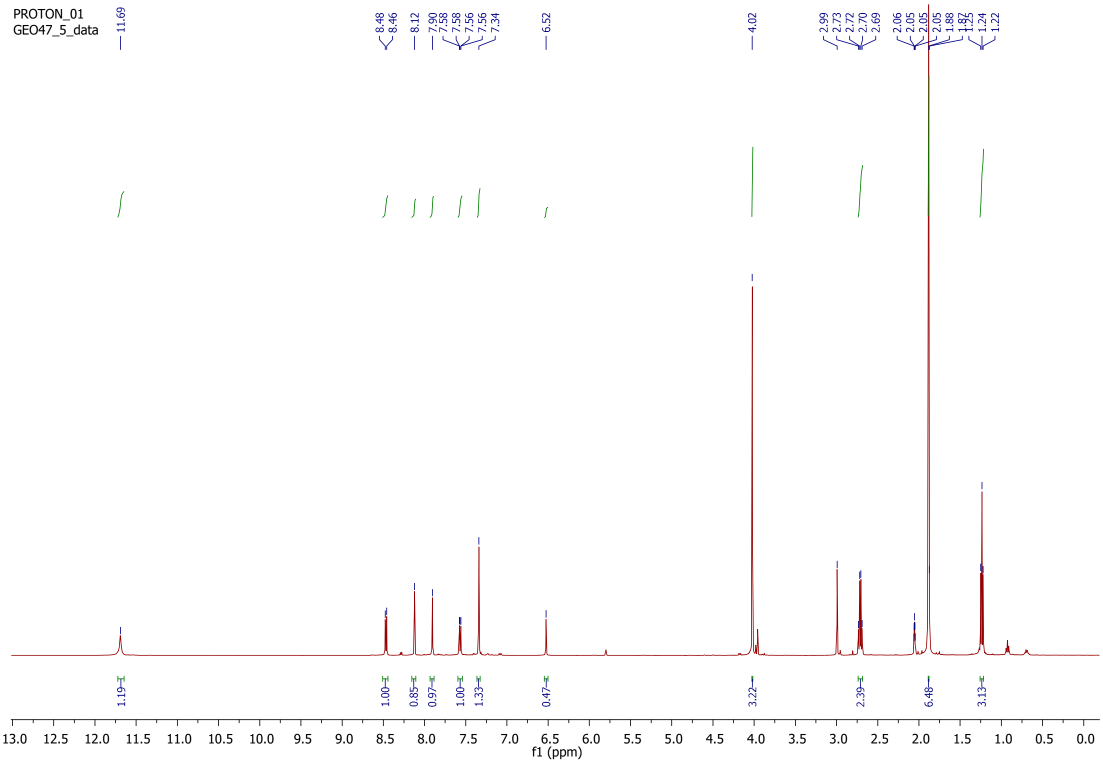

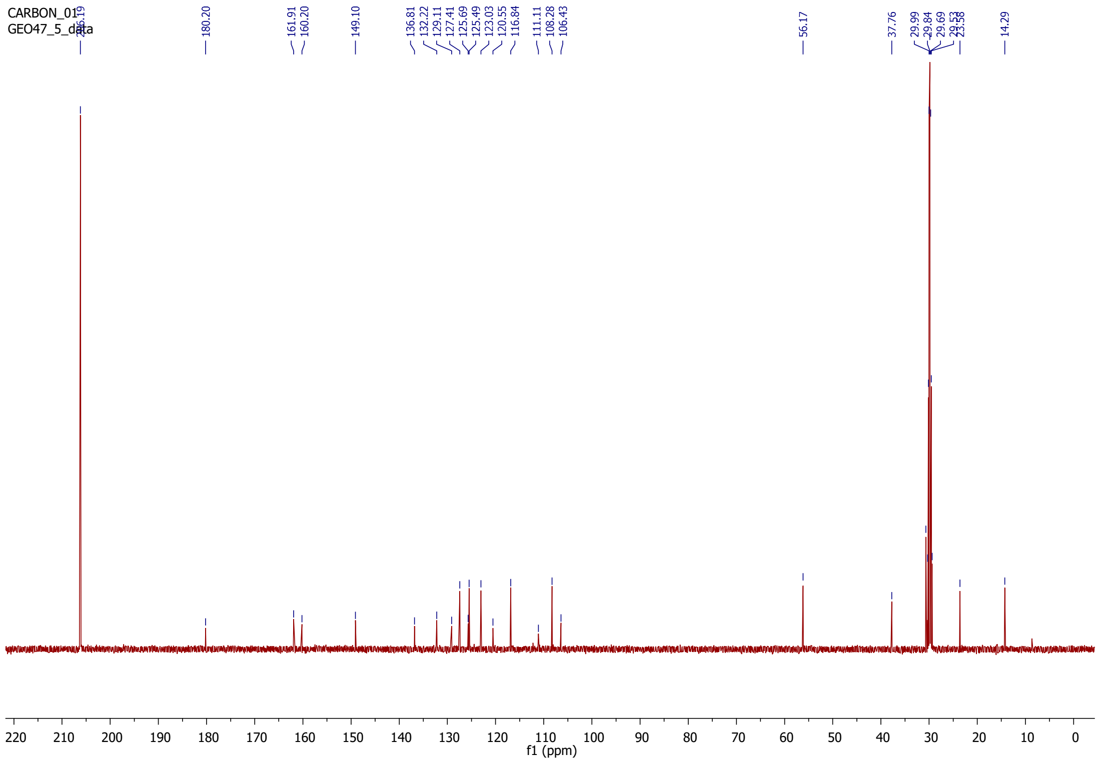

Figure S13. ^{1}H-NMR and ^{13}C-NMR spectra for compound **8**

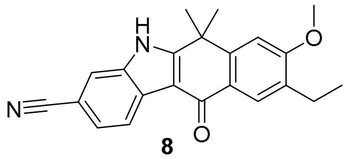

8

Method 3

Retention time: 29.4 m

area: 31599537 (absorbance units x minutes)

total area: 34213713 (absorbance units x minutes)

area %: (31599537/34213713)*100 = 92.3 %

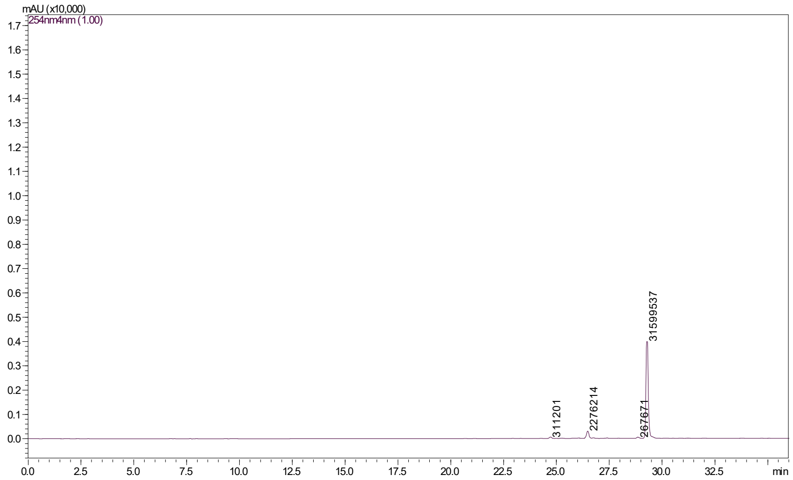

Figure S14. ESI-LCMS of compound **8** after column chromatography purification

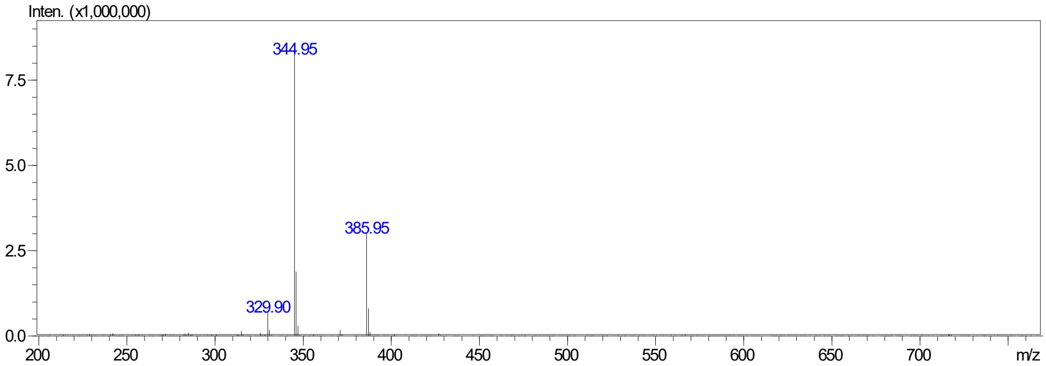

Figure S15. ESI-MS for **8**, positive mode: m/z calcd mass for $C_{22}H_{20}N_2O_2$ $[M]^+$=344.15, was found 344.95

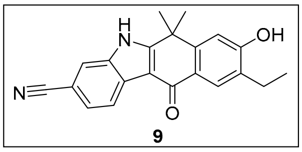

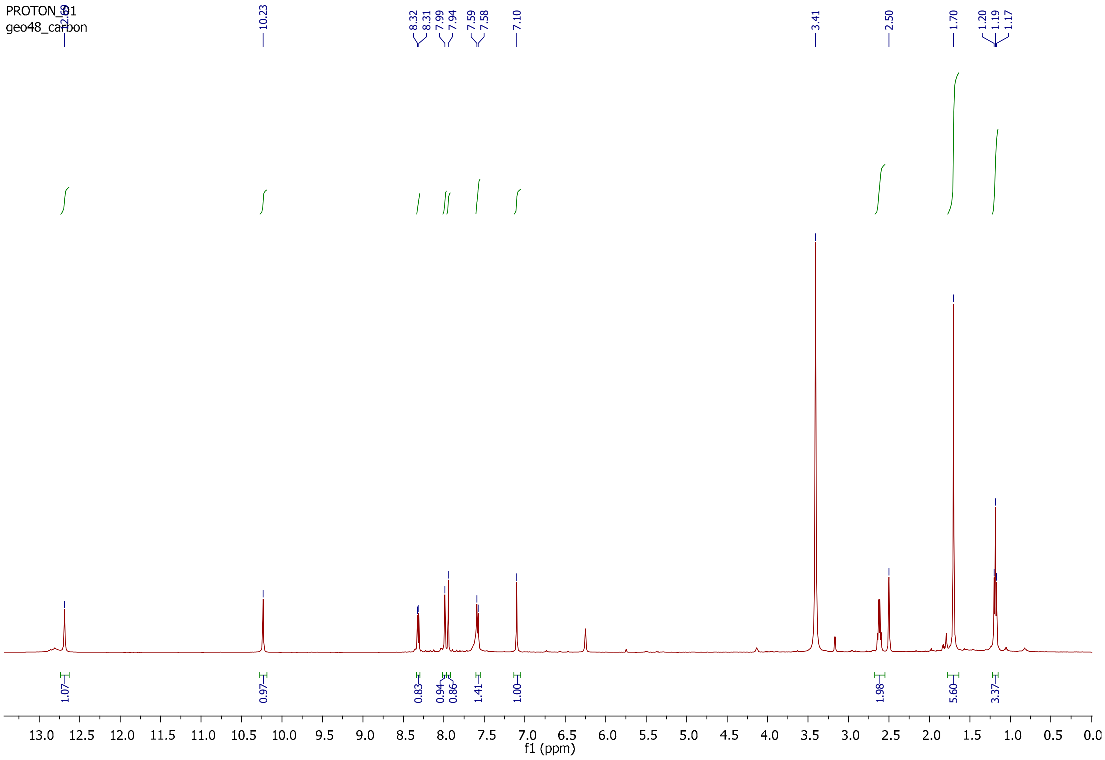

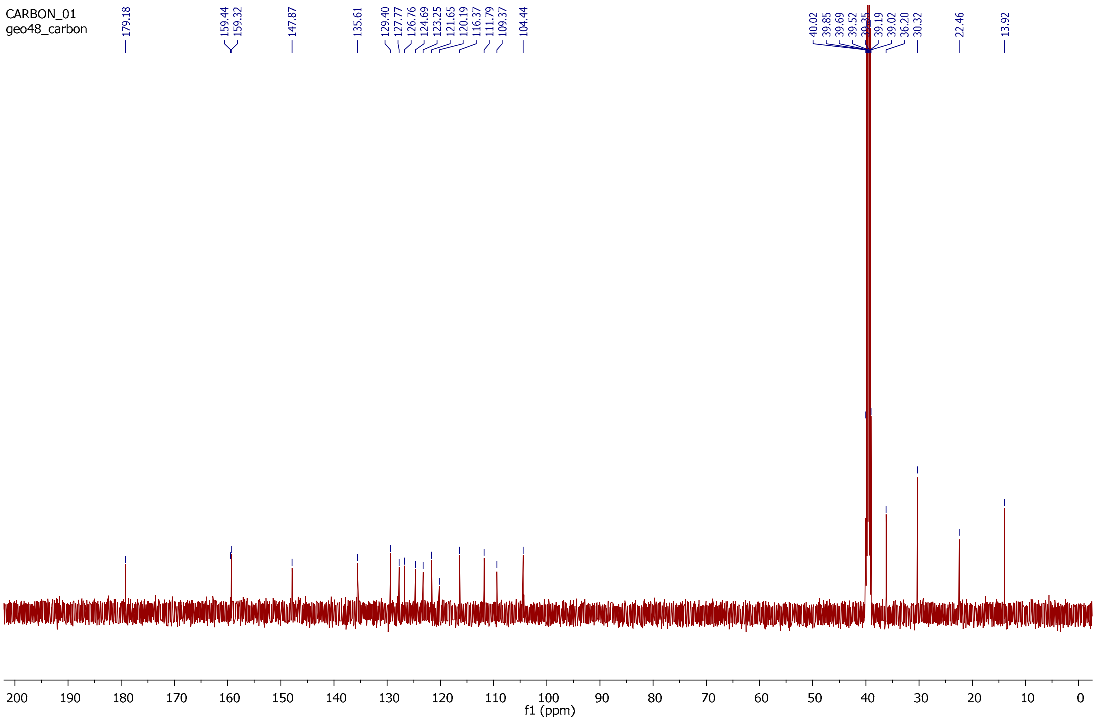

Figure S16. ^{1}H-NMR and ^{13}C-NMR spectra for compound **9**

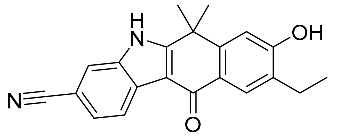

9

MeOH isocratic elution

Retention time: 10.5 m

area: 93581467 (absorbance units x minutes)

total area: 93806687 (absorbance units x minutes)

area %: (93581467/93806687)*100 = 99.7 %

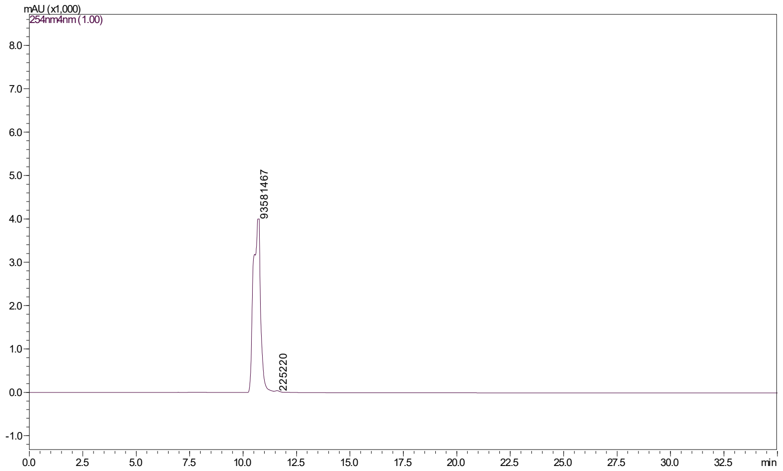

Figure S17. ESI-LCMS of compound **9** after column chromatography purification

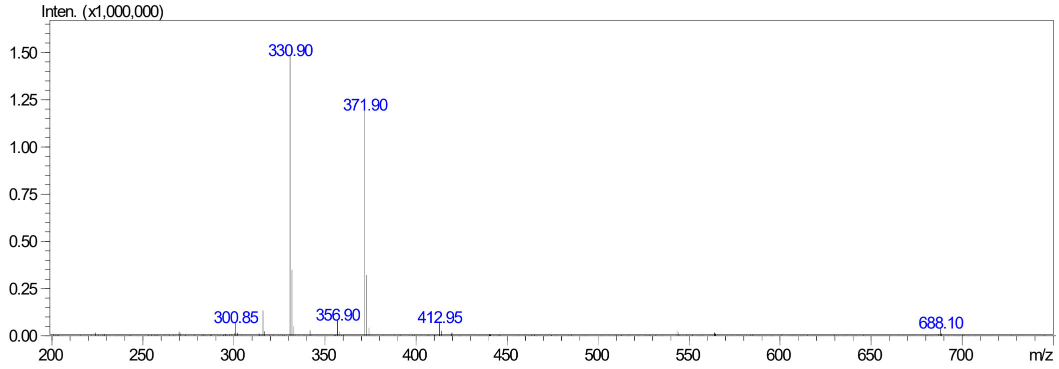

Figure S18. ESI-MS for **9**, positive mode: m/z calcd mass for $C_{21}H_{18}N_2O_2$ $[M+H]^+$=330.14, was found 330.90

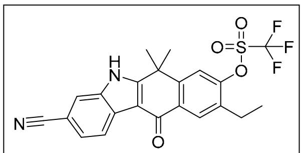

10

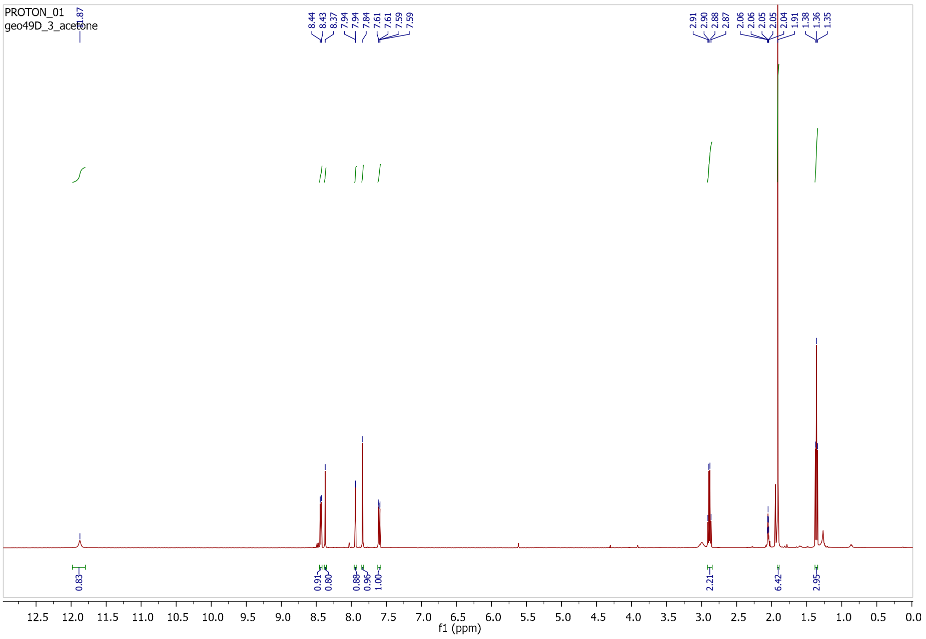

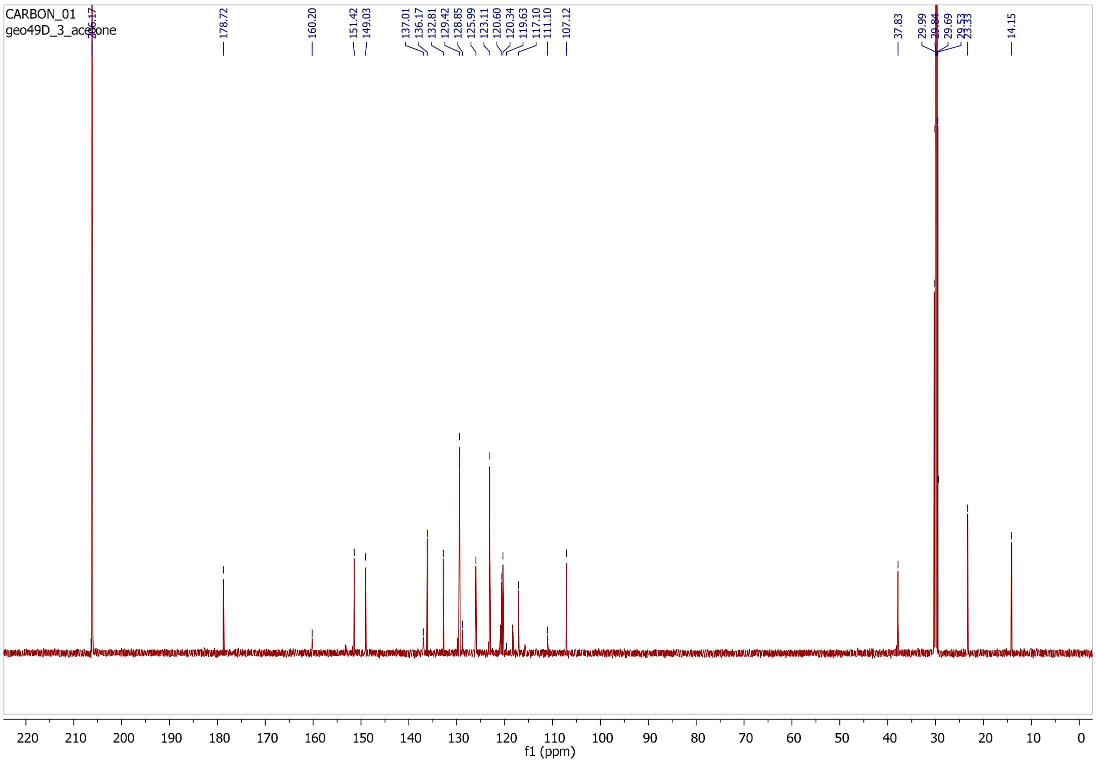

Figure S19. ^{1}H-NMR and ^{13}C-NMR spectra for compound **10**

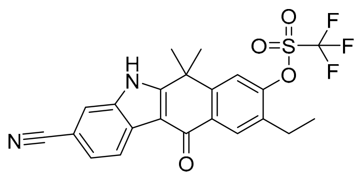

10

Method 1

Retention time: 32.1 m

area: 28426895 (absorbance units x minutes)

total area: 29099709 (absorbance units x minutes)

area %: (28426895/29099709)*100 = 97.7 %

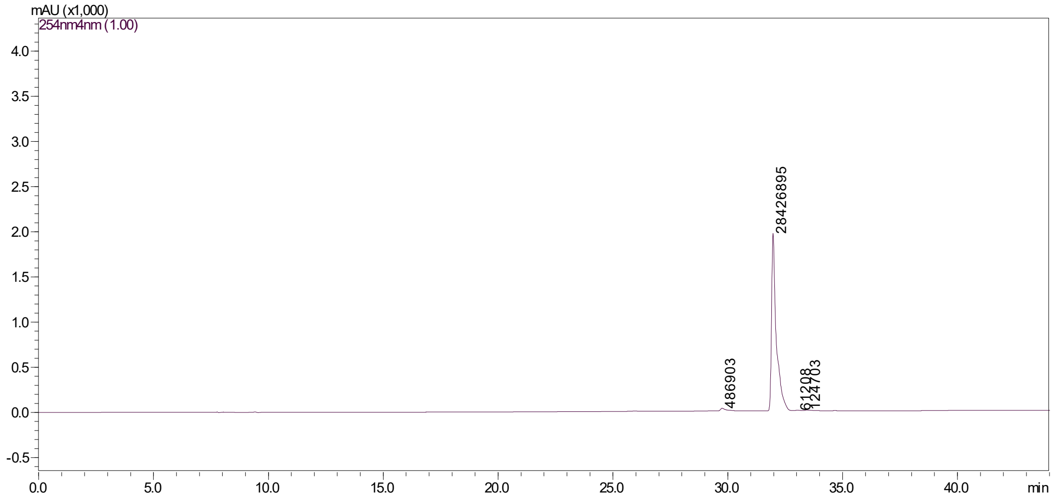

Figure S20. ESI-LCMS of compound **10** after column chromatography purification

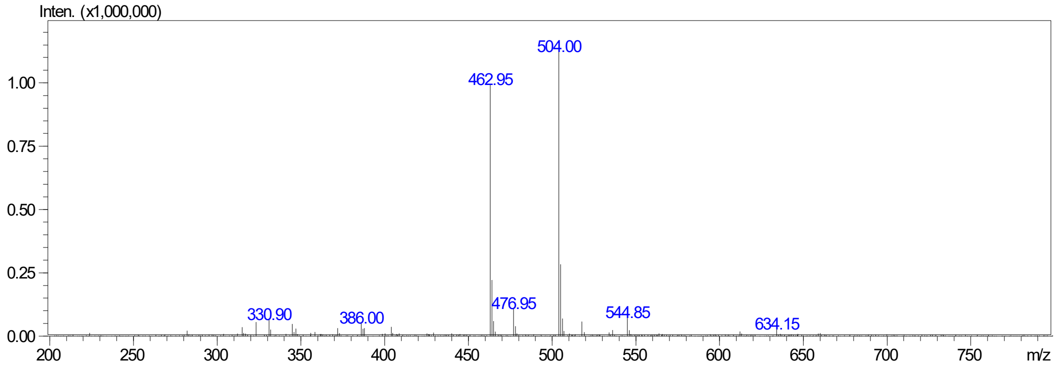

Figure S21. ESI-MS for **10**, positive mode: m/z calcd mass for $C_{22}H_{17}F_3N_2O_4S$ $[M]^+$=462.08, was found 462.95

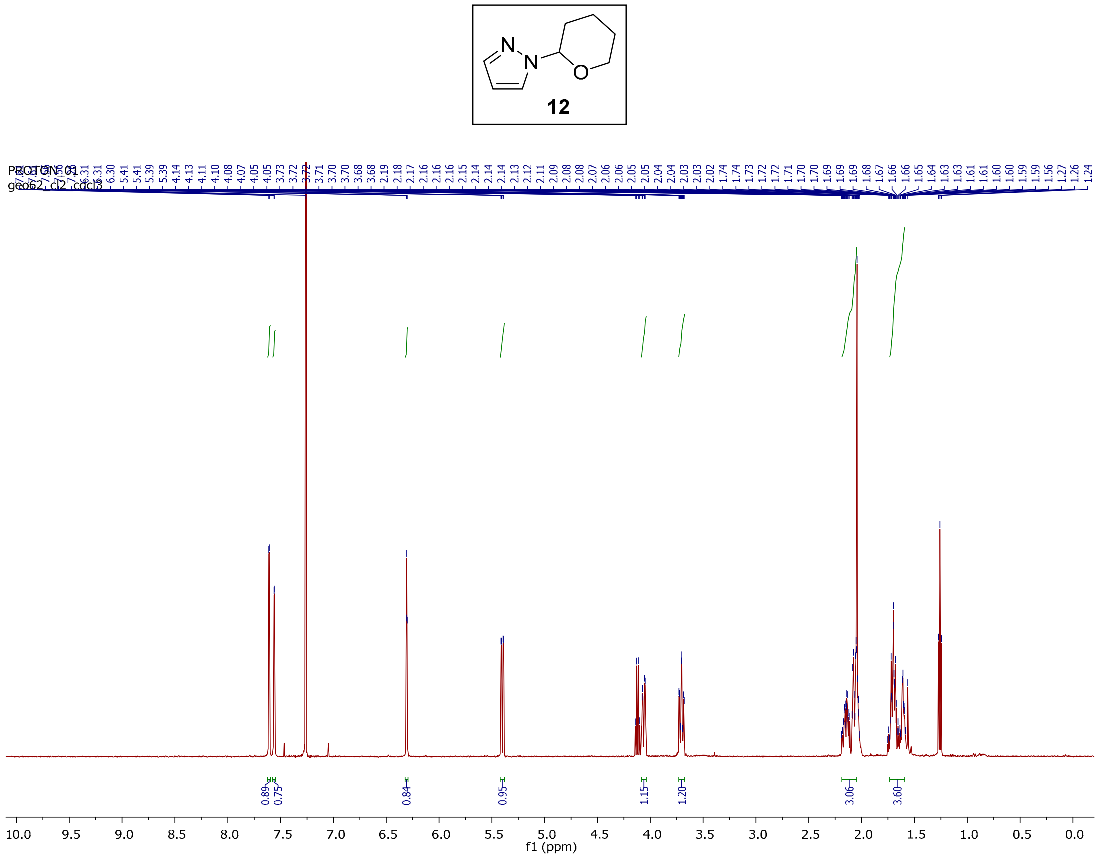

Figure S22. ^{1}H-NMR for compound **12**

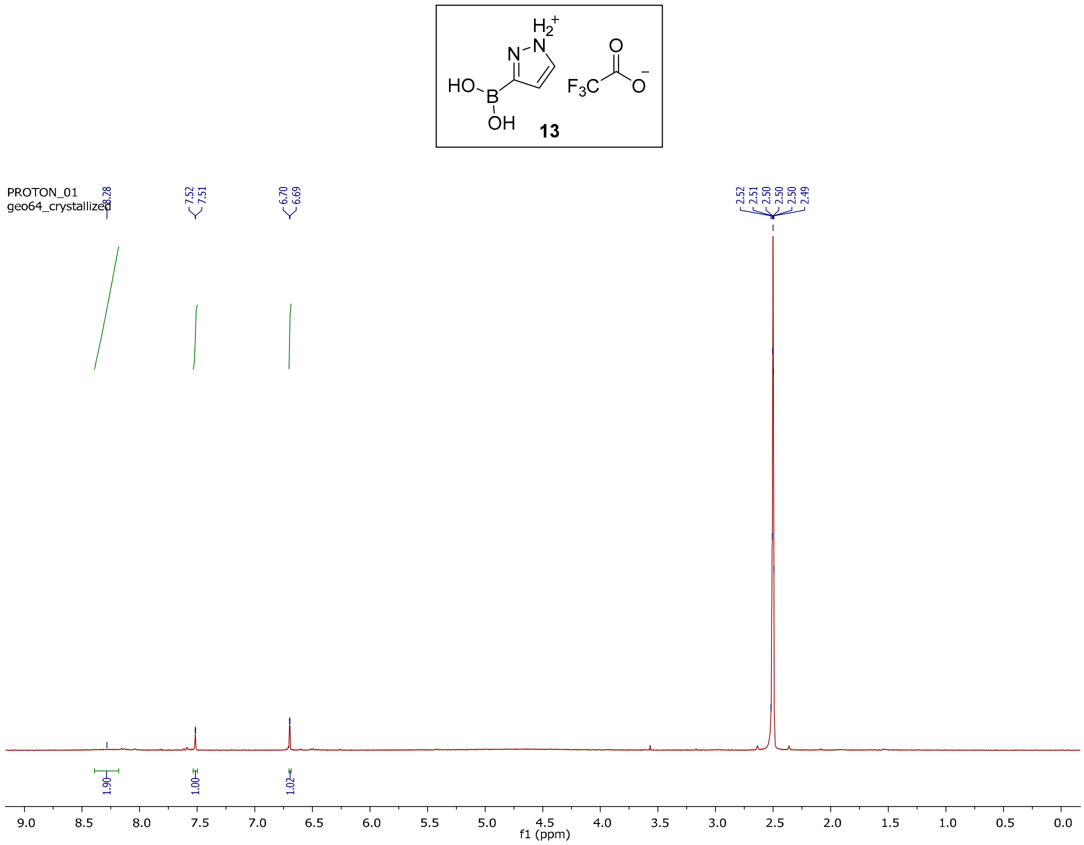

Figure S23. ^{1}H-NMR for compound **13**

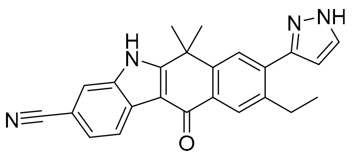

JH-VII-139-1

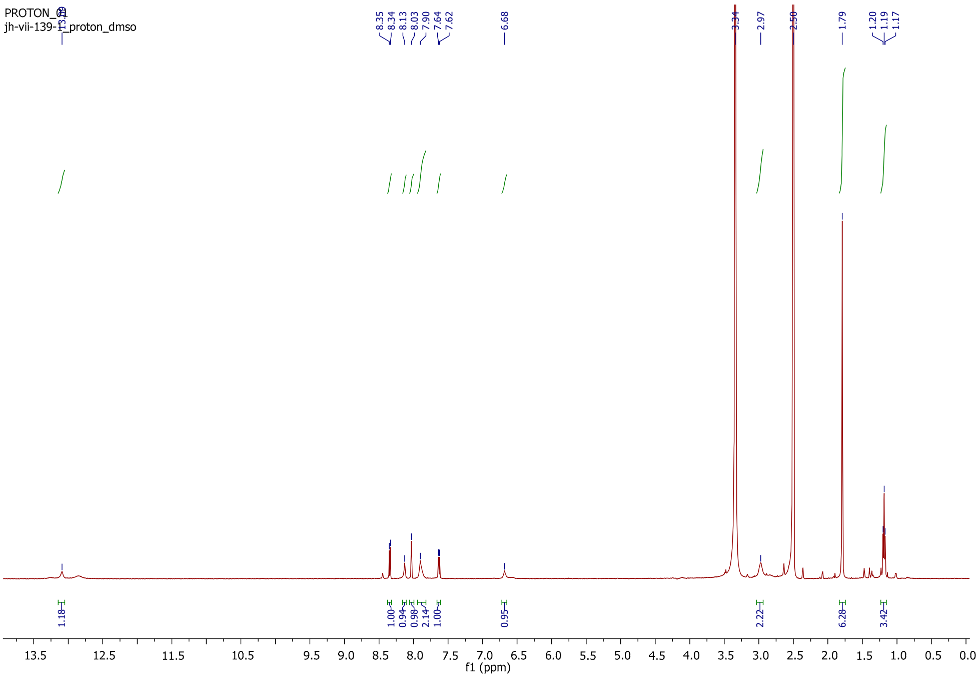

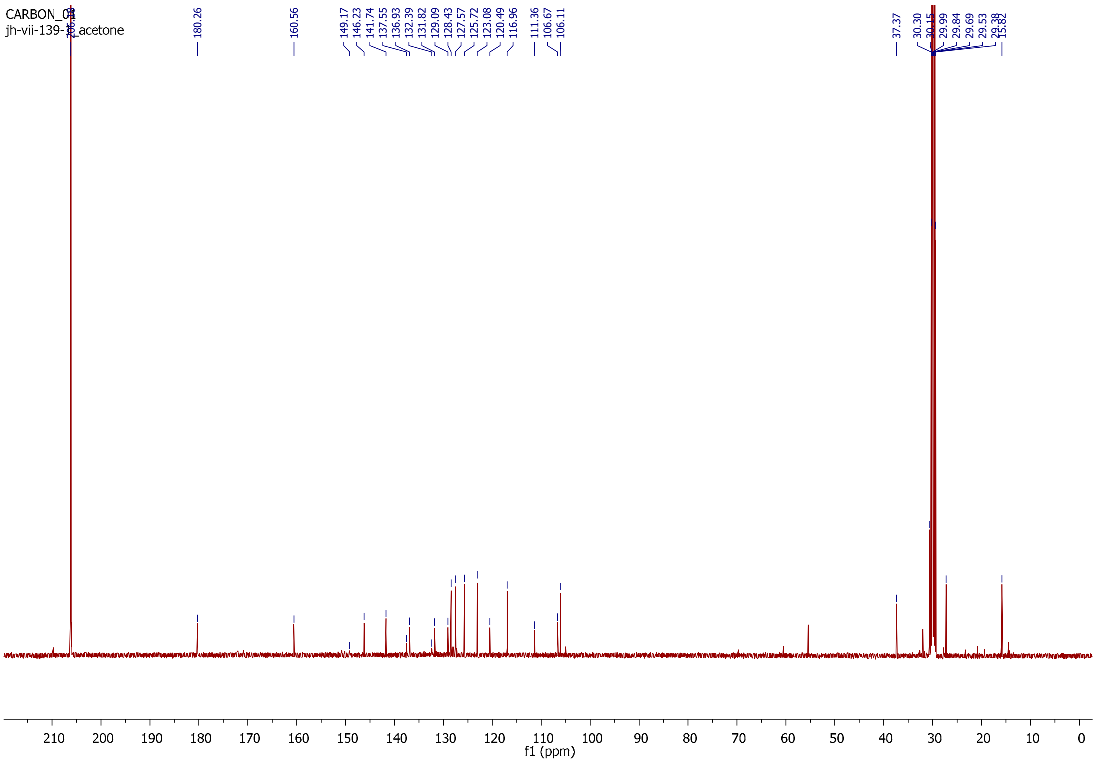

Figure S24. ^{1}H-NMR and ^{13}C-NMR spectra for compound **JH-VII-139-1**

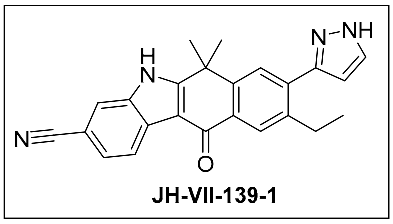

Method 1

Retention time: 26.3 m

area: 8070456 (absorbance units x minutes)

total area: 8258696 (absorbance units x minutes)

area %: (8070456/8258696)*100 = 97.7 %

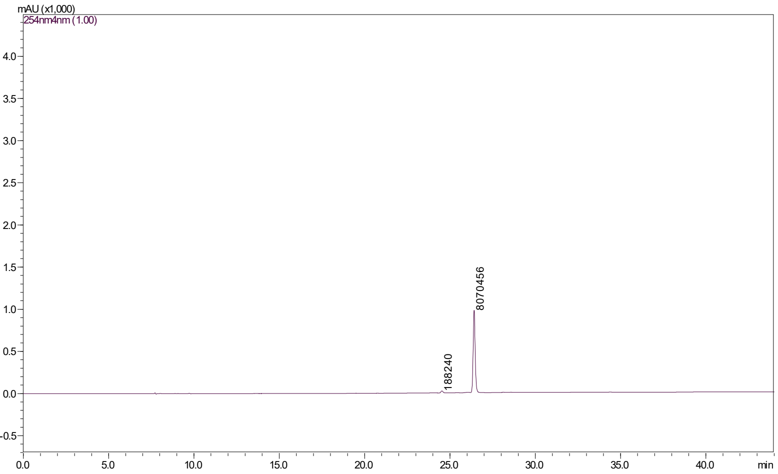

Figure S25. ESI-LCMS of compound **JH-VII-139-1** after column chromatography purification

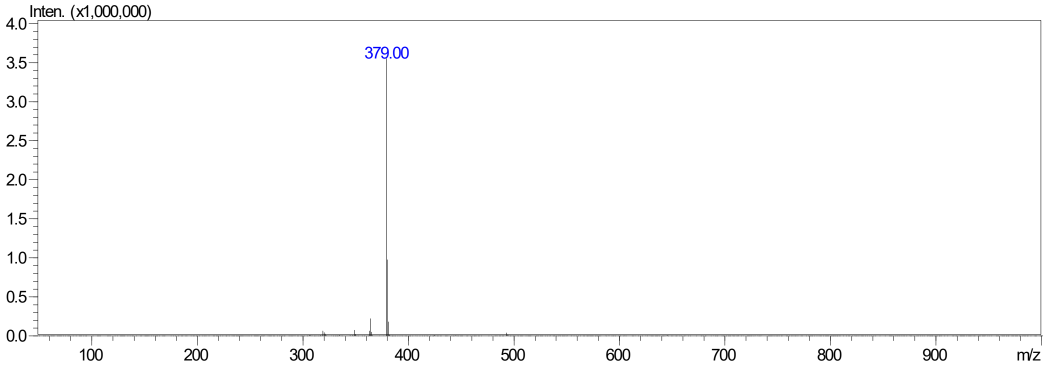

Figure S26. ESI-MS for **JH-VII-139-1**, negative mode: m/z calcd mass for $C_{24}H_{20}N_4O$ $[M-H]^-$ =379.16, was found 379.00

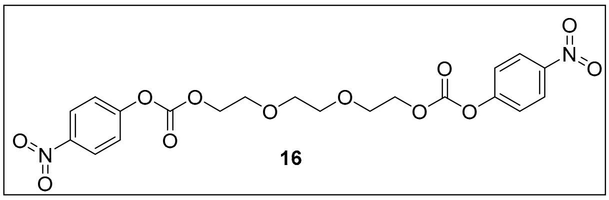

16

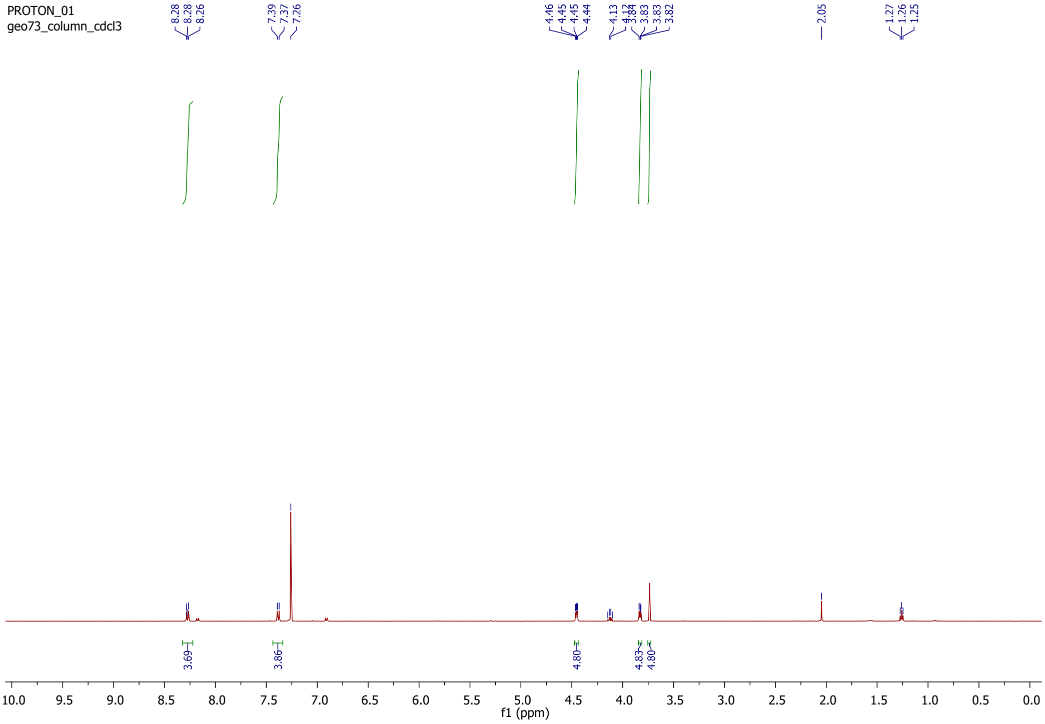

Figure S27. ^{1}H-NMR spectrum for compound **16**

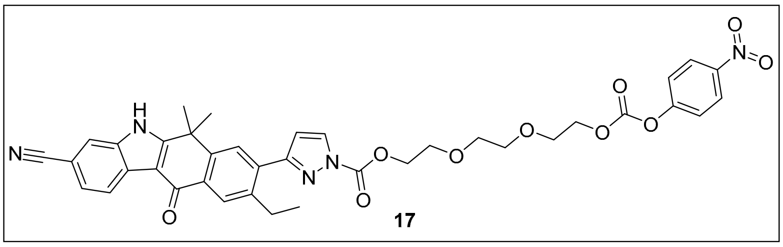

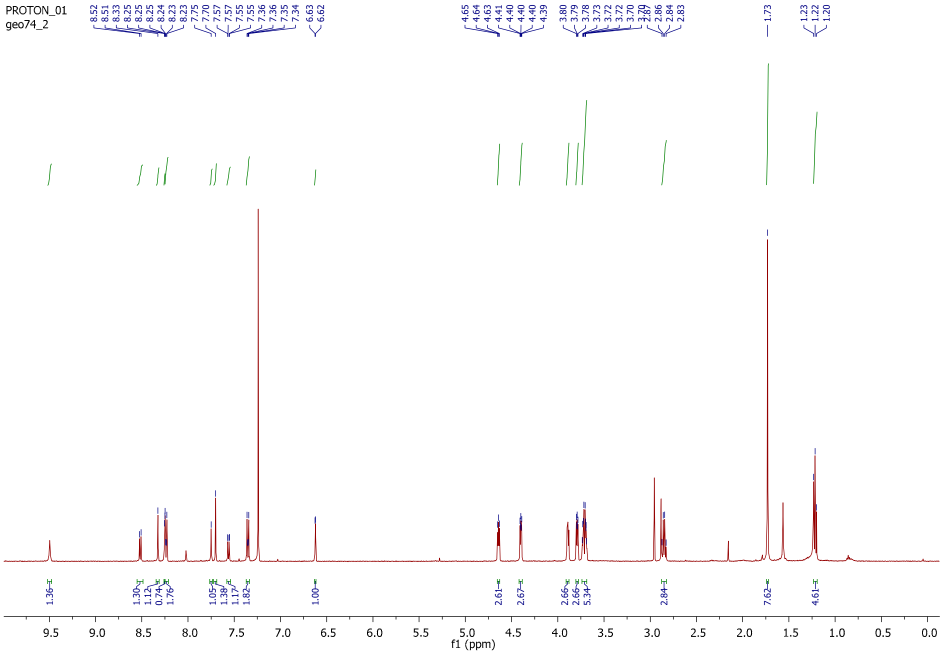

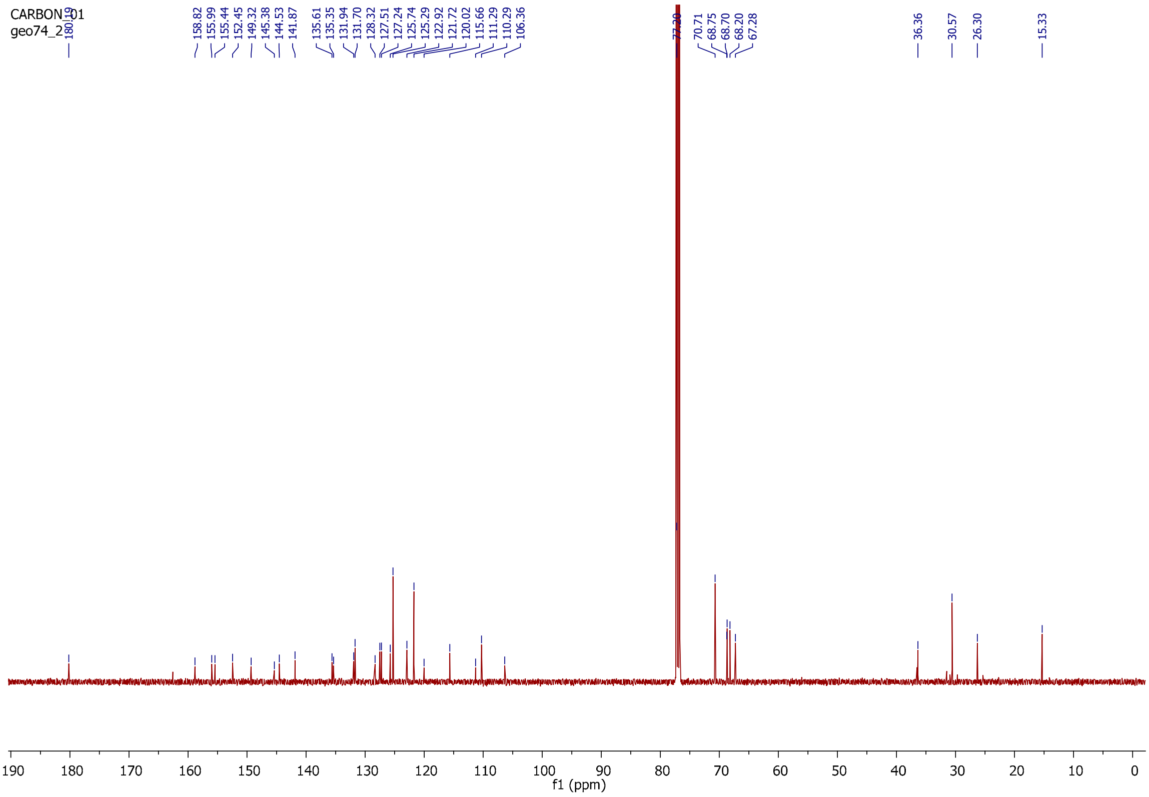

Figure S28. ^{1}H-NMR and ^{13}C-NMR spectra for compound **17**

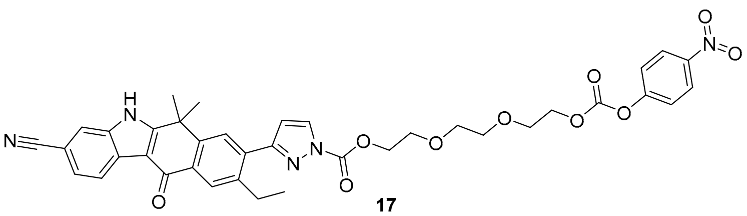

17

Method 1

Retention time: 29.5 m

area: 25509809 (absorbance units x minutes)

total area: 26264849 (absorbance units x minutes)

area %: (25509809/26264849)*100 = 97.1 %

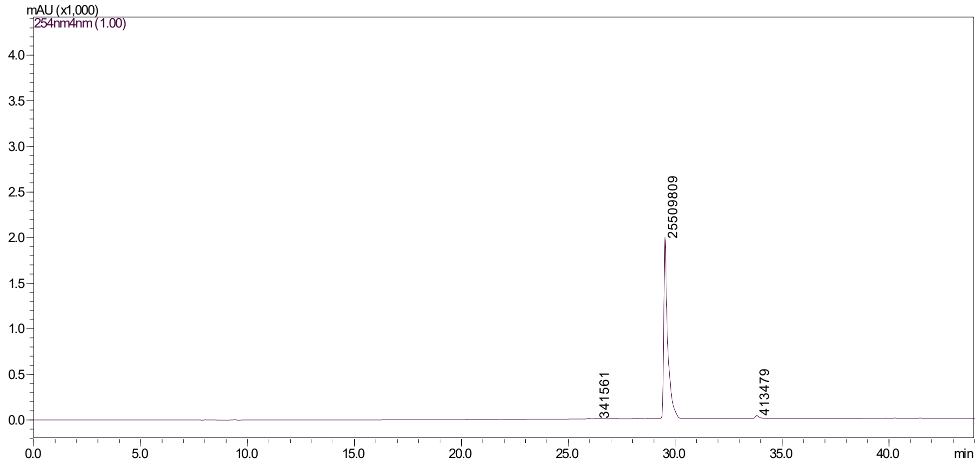

Figure S29. ESI-LCMS of compound **17** after column chromatography purification

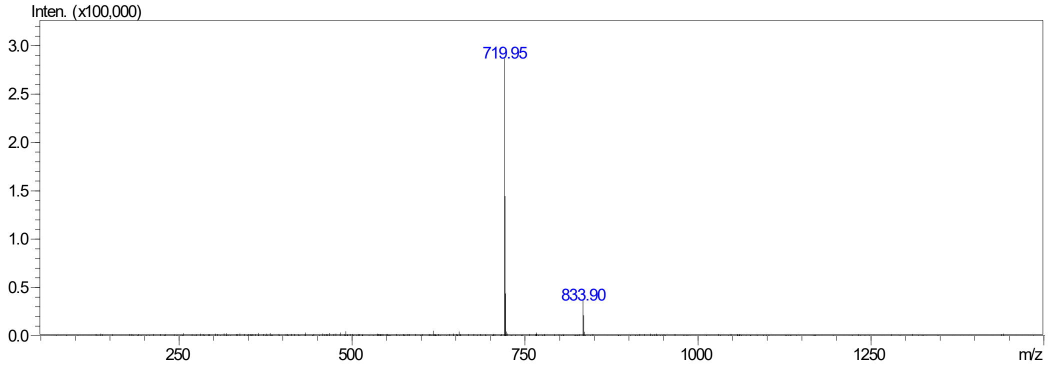

Figure S30. ESI-MS for **17**, negative mode: m/z calcd mass for $C_{38}H_{35}N_5O_{10}$ $[M+H]^+$=720.24, was found 719.95

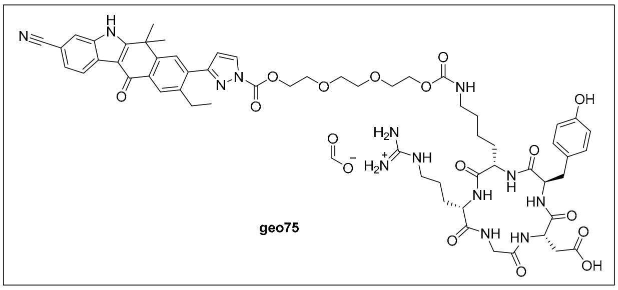

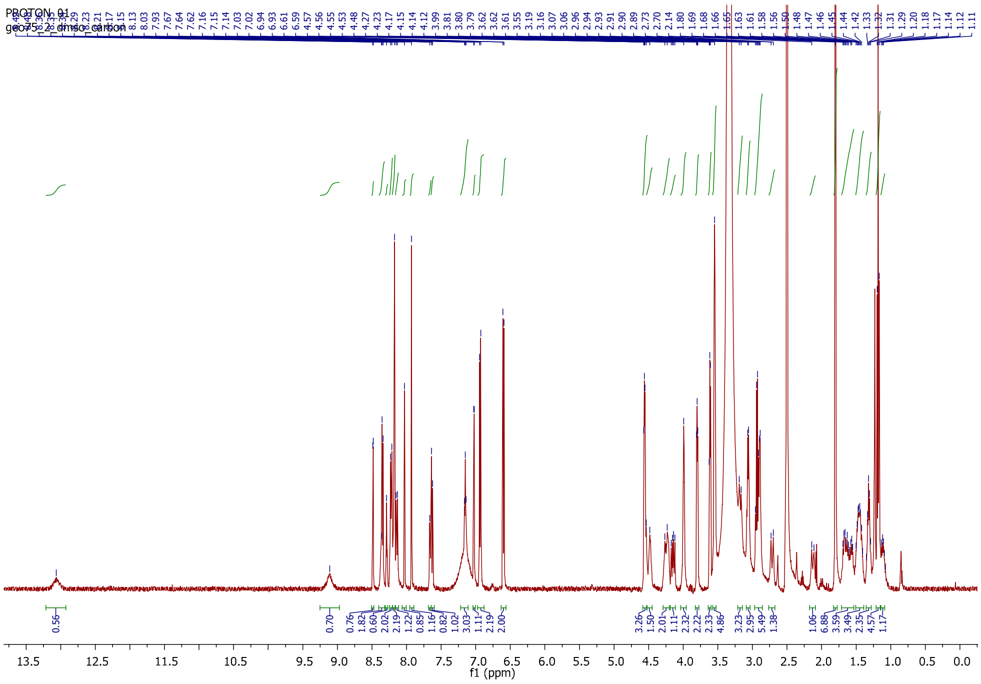

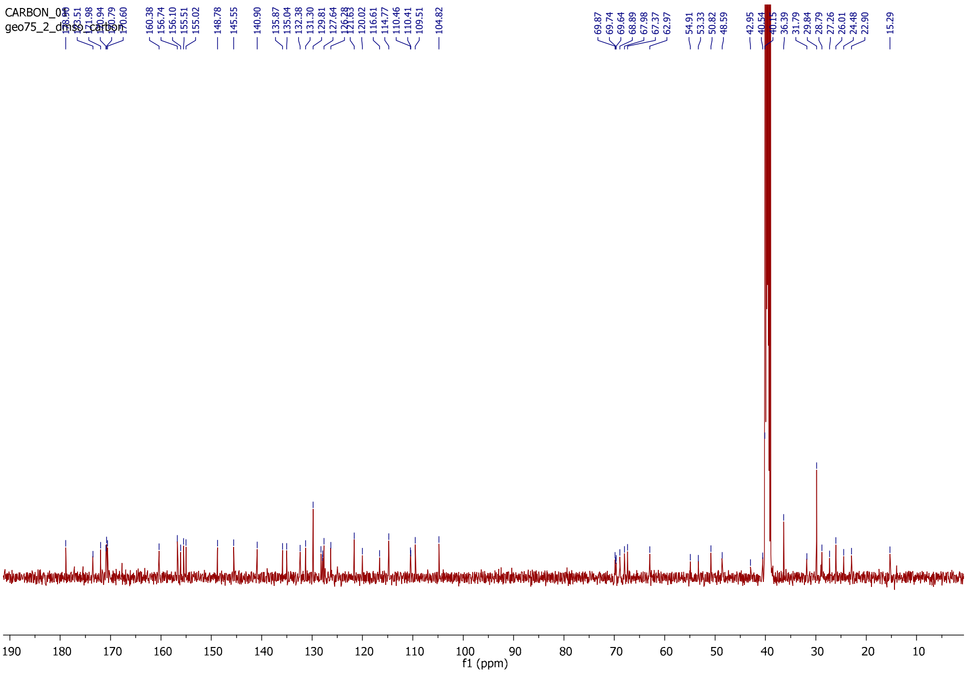

Figure S31. ^{1}H-NMR and ^{13}C-NMR spectra for compound **geo75**

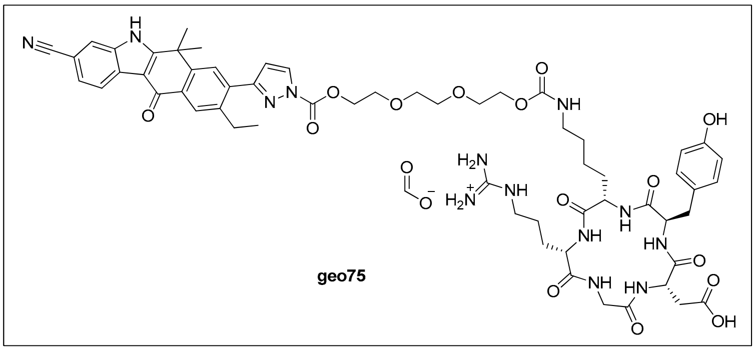

Method 1

Retention time: 20.9 m

area: 32830161 (absorbance units x minutes)

total area: 33143414 (absorbance units x minutes)

area %: (32830161/33143414)*100 = 99.0 %

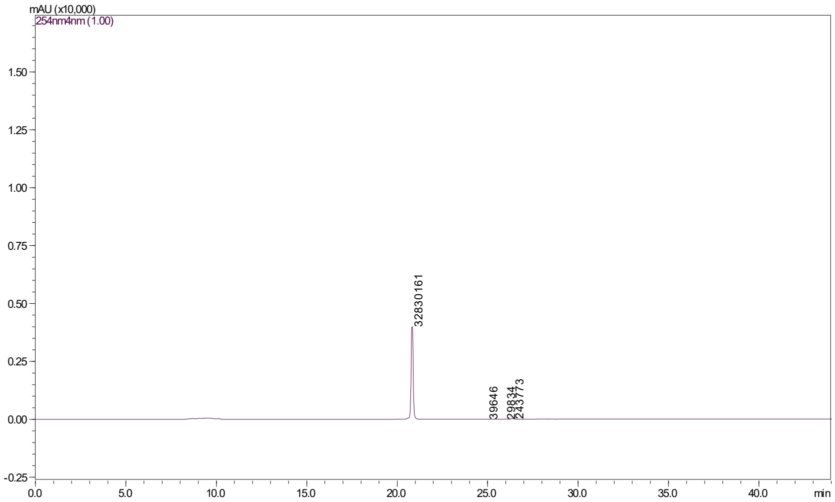

Figure S32. ESI-LCMS of compound **geo75** after column chromatography purification

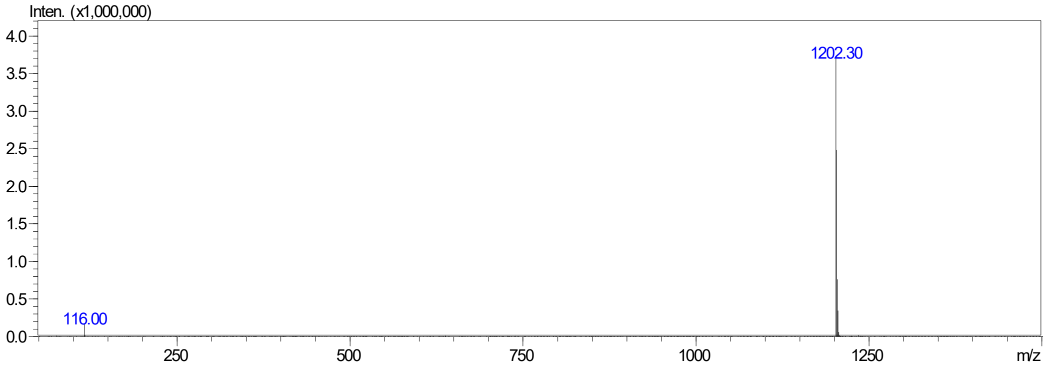

Figure S33. ESI-MS for **geo75**, positive mode: m/z calcd mass for $C_{59}H_{71}N_{13}O_{15}$ $[M+H]^+$=1202.52, was found 1202.30.

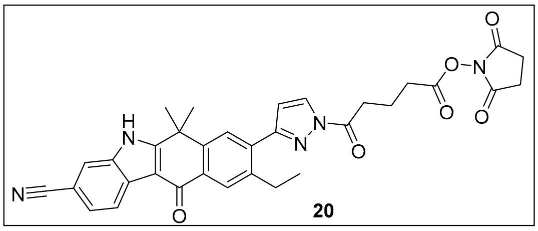

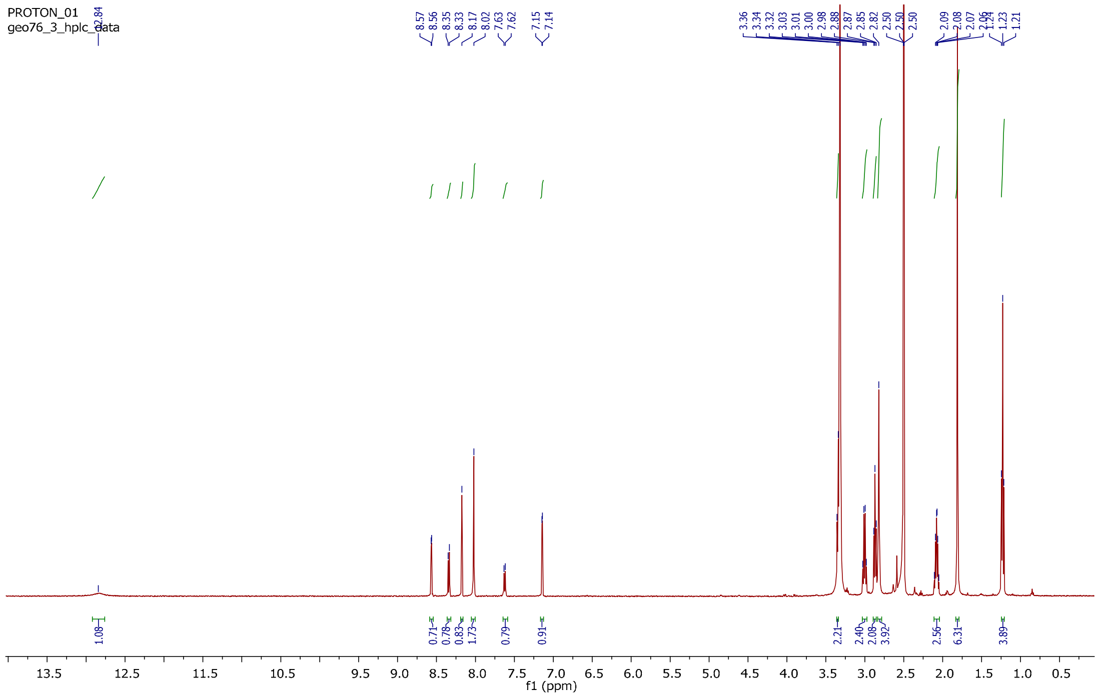

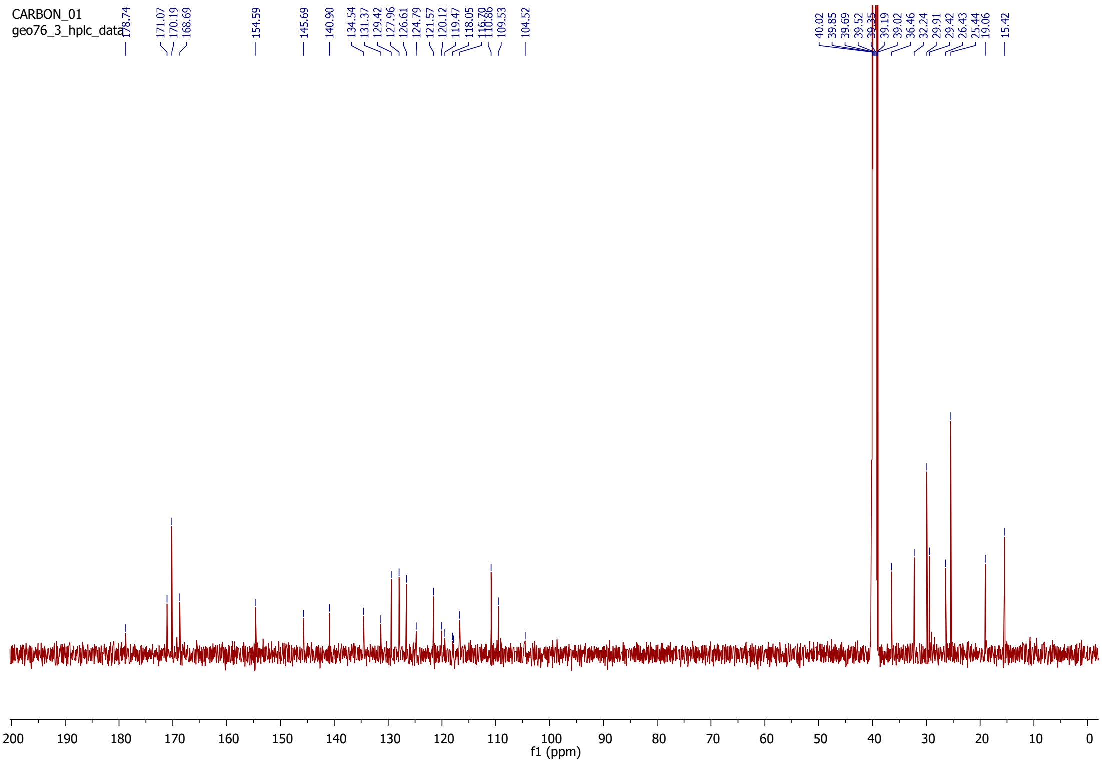

Figure S34. ^{1}H-NMR and ^{13}C-NMR spectra for compound **20**

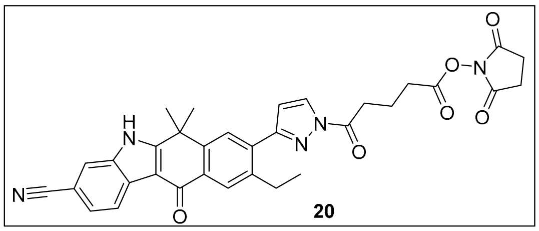

Method 1

Retention time: 28.9 m

area: 7423047 (absorbance units x minutes)

total area: 8244264 (absorbance units x minutes)

area %: (7423047/8244264)*100 = 90.1 %

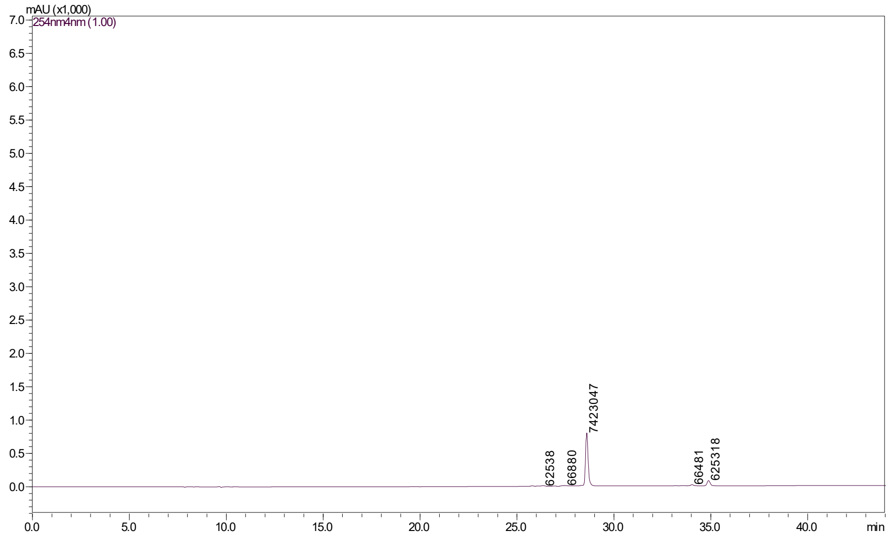

Figure S35. ESI-LCMS of compound **20** after column chromatography purification

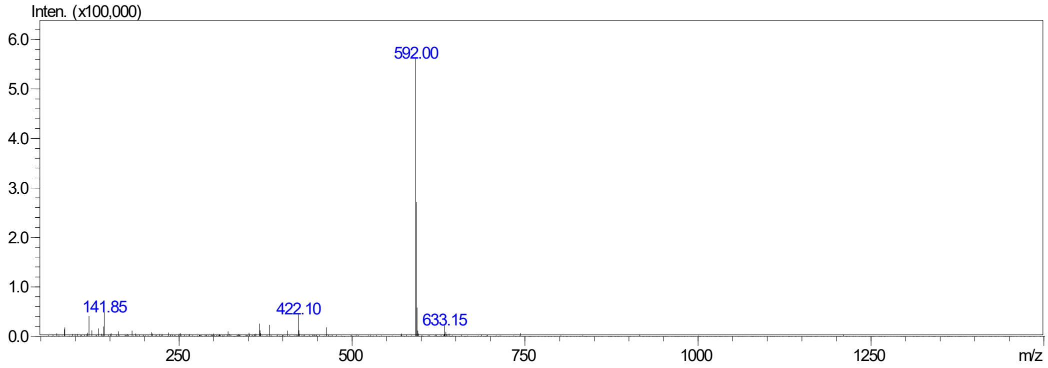

Figure S36. ESI-MS for **20**, positive mode: m/z calcd mass for $C_{33}H_{29}N_5O_6$ $[M+H]^+$=592.21, was found 592.00

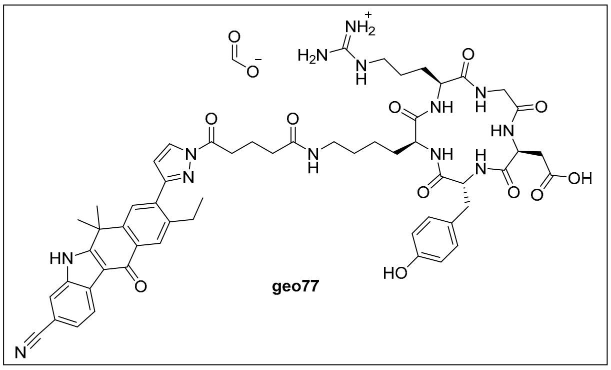

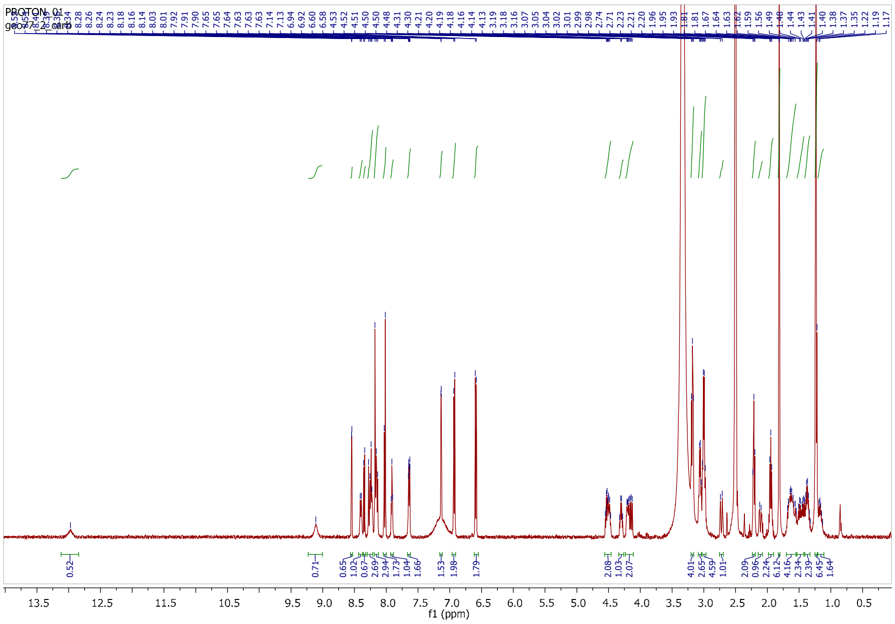

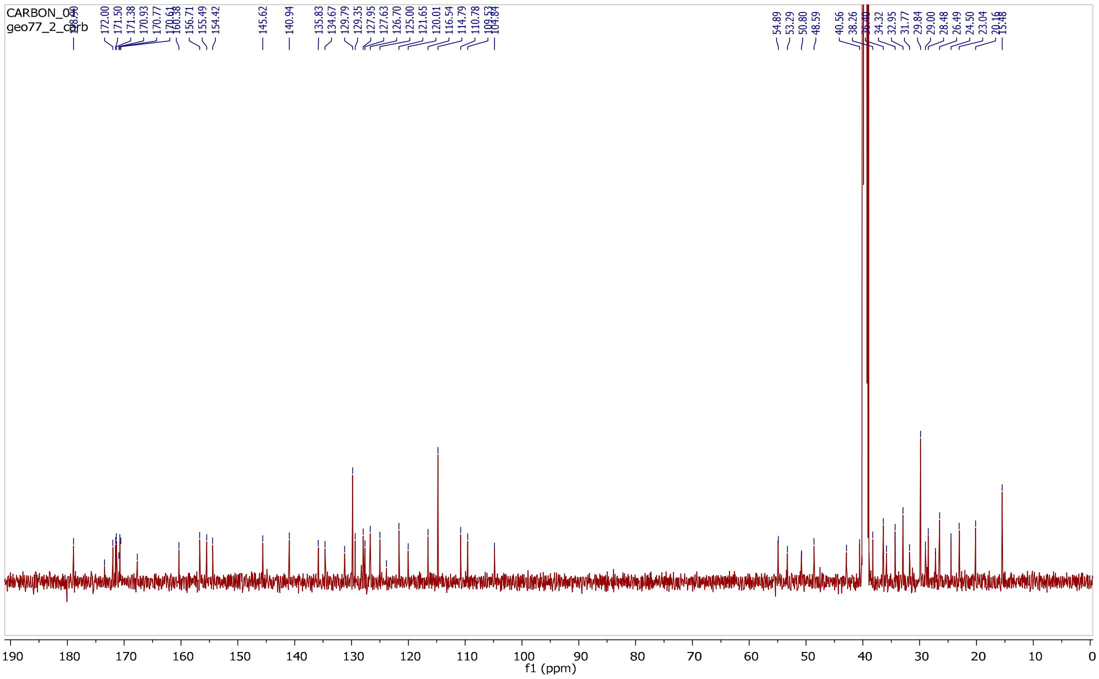

Figure S37. ^{1}H-NMR and ^{13}C-NMR spectra for compound **geo77**.

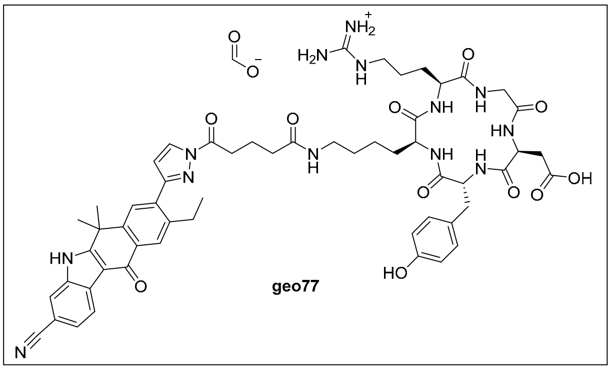

Method 1

Retention time: 20.9 m

area: 54752812 (absorbance units x minutes)

total area: 55219719 (absorbance units x minutes)

area %: (54752812/55219719)*100 = 99.1 %

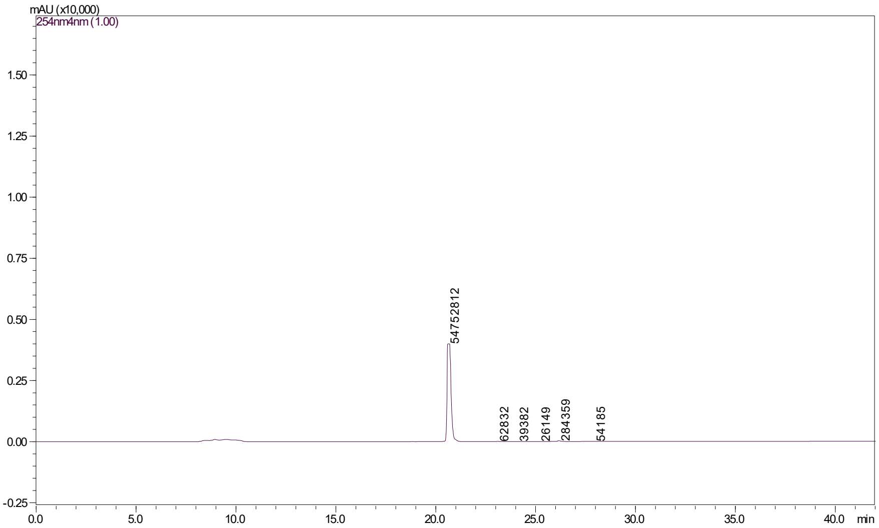

Figure S38. ESI-LCMS of compound **geo77** after HPLC purification.

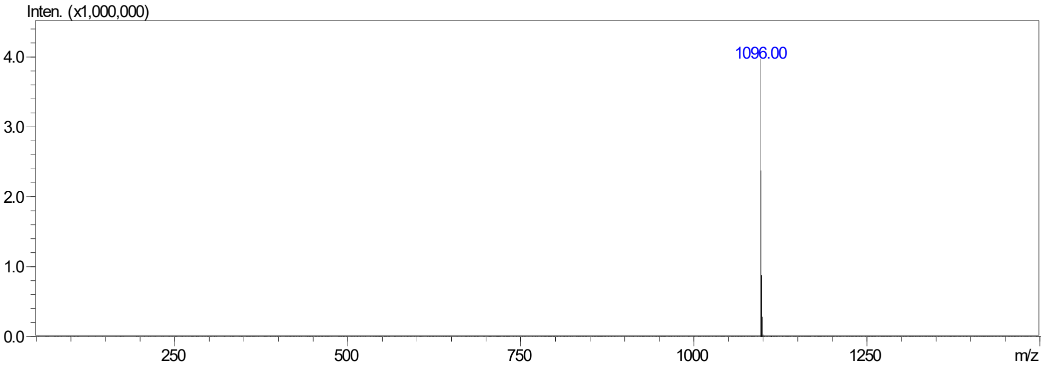

Figure S39. ESI-MS for **geo77**, positive mode: m/z calcd mass for $C_{56}H_{66}N_{13}O_{11}$ $[M+H]^+$=1096.49, was found 1096.00.

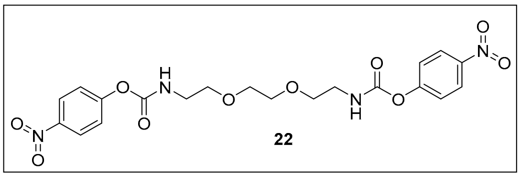

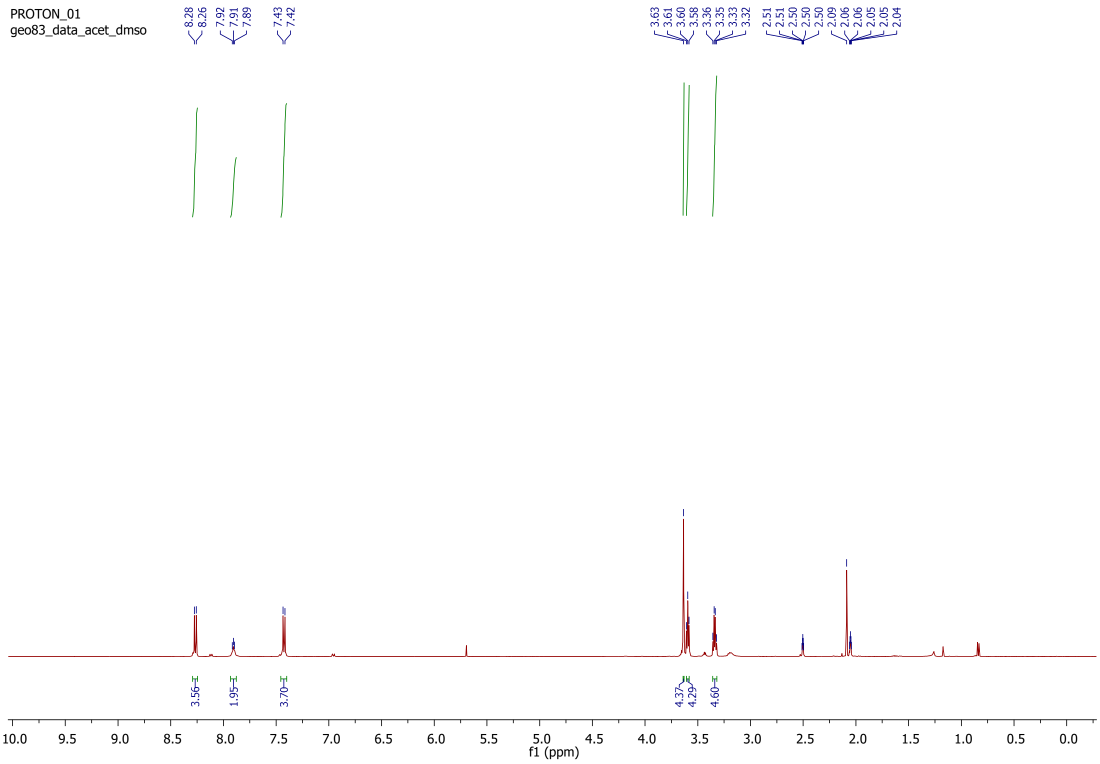

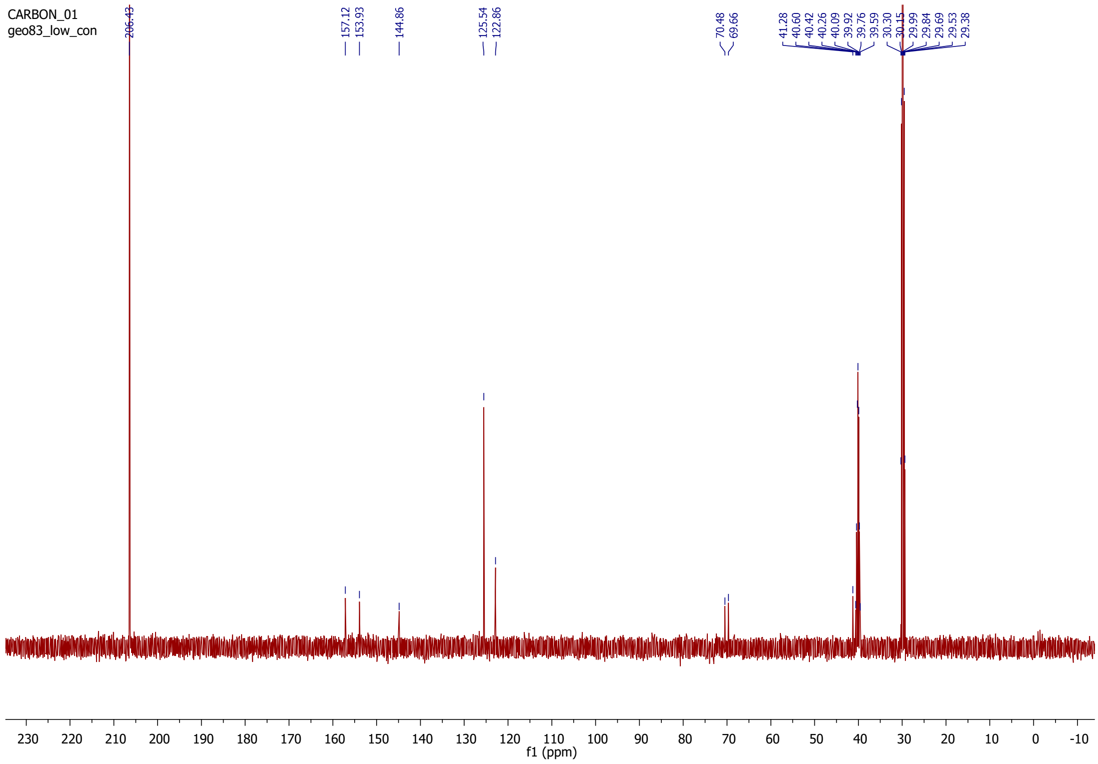

Figure S40. ^{1}H-NMR and ^{13}C-NMR spectra for compound **22**.

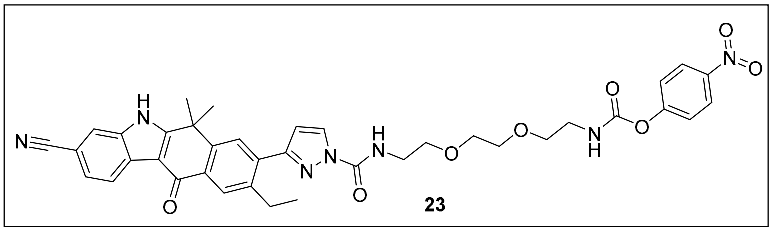

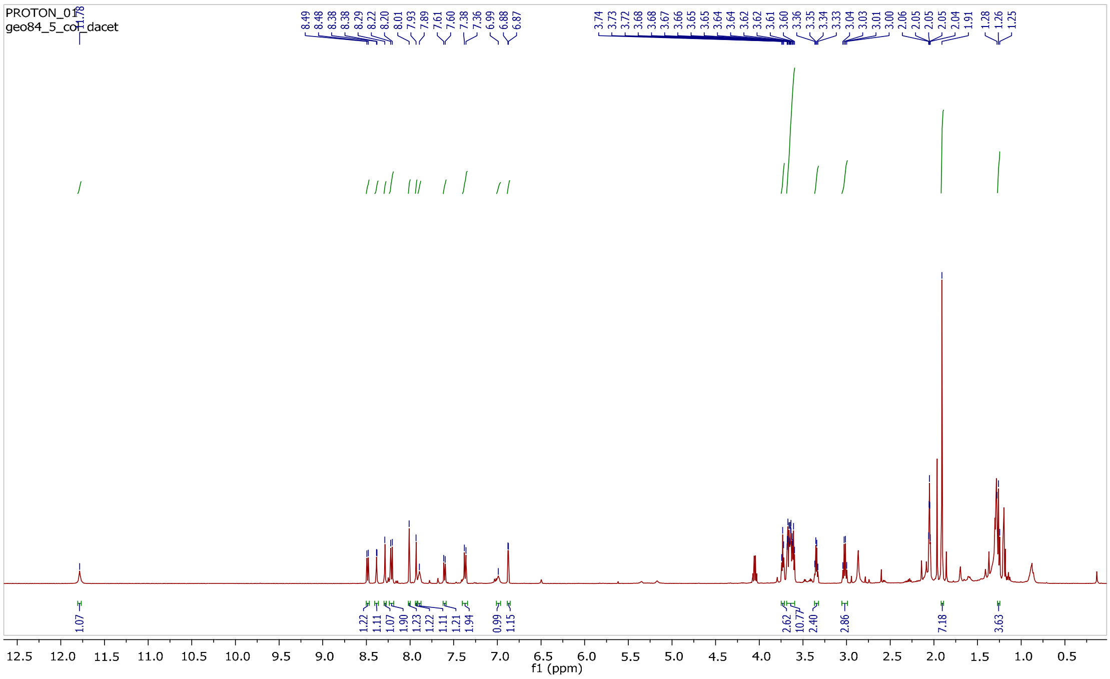

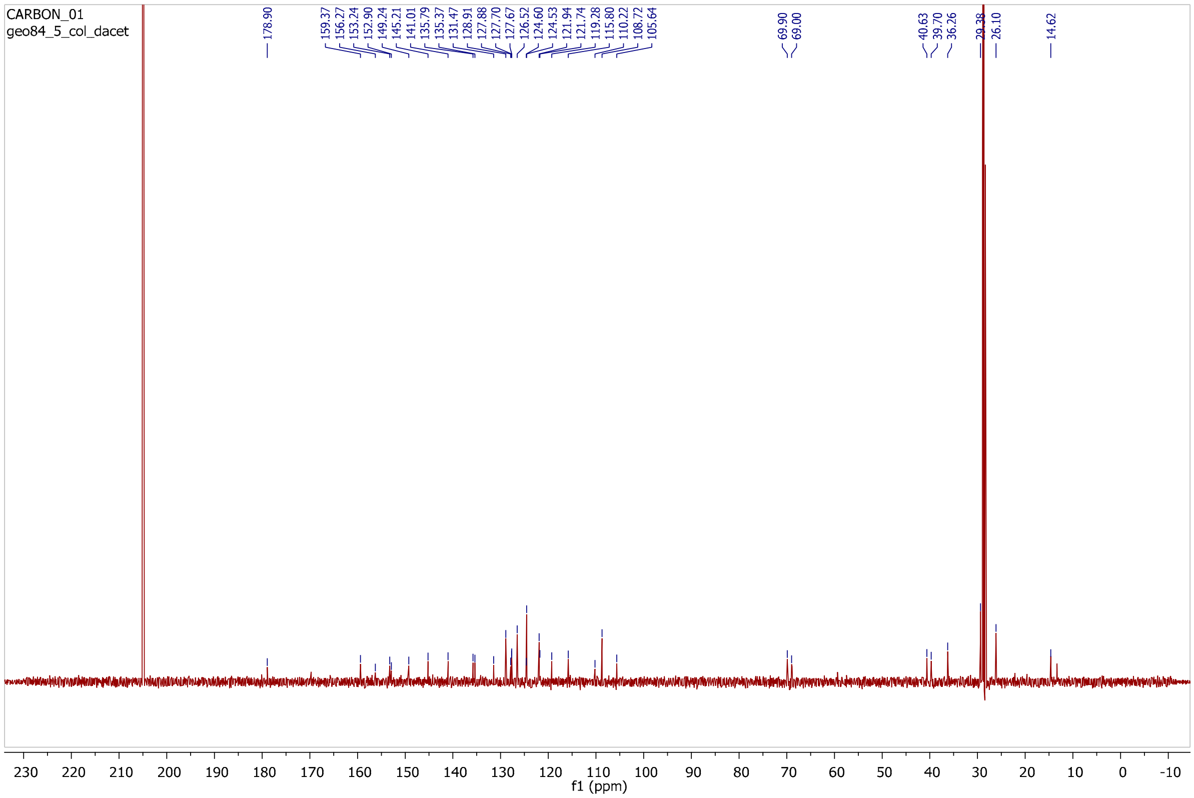

Figure S41. ^{1}H-NMR and ^{13}C-NMR spectra for compound **23**.

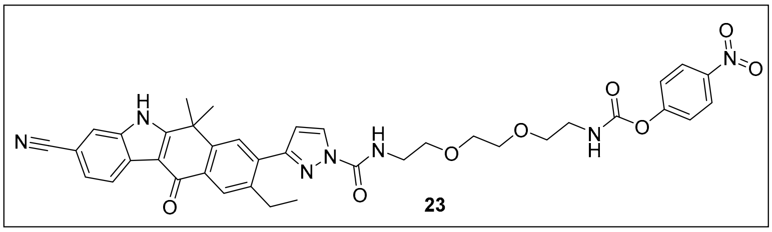

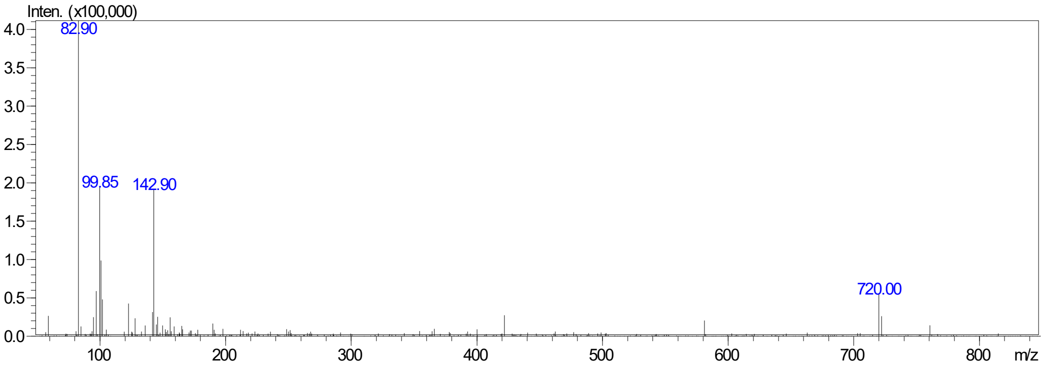

Figure S42. ESI-MS for **23**, positive mode: m/z calcd mass for $C_{38}H_{38}N_7O_8$ $[M+H]^+$=720.27, was found 720.00

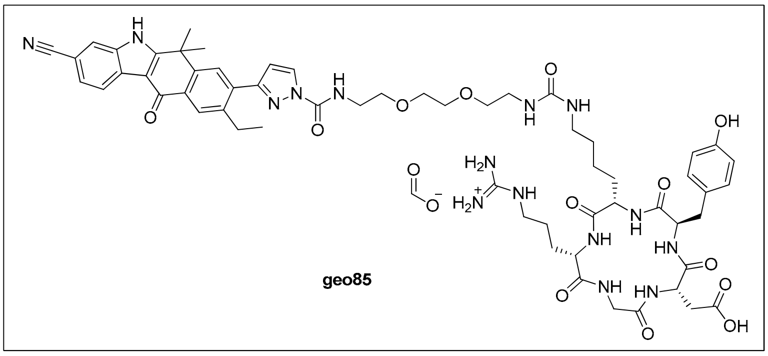

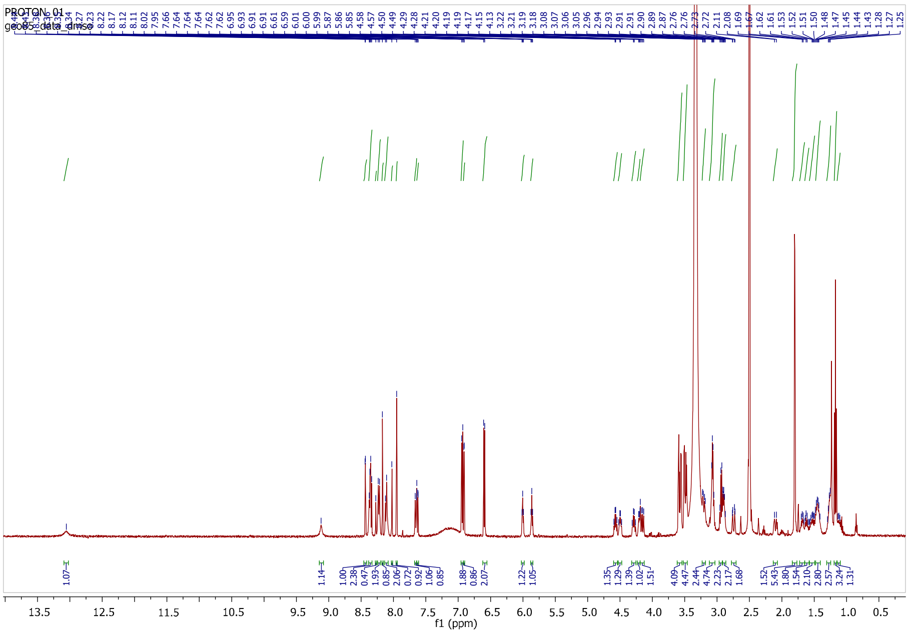

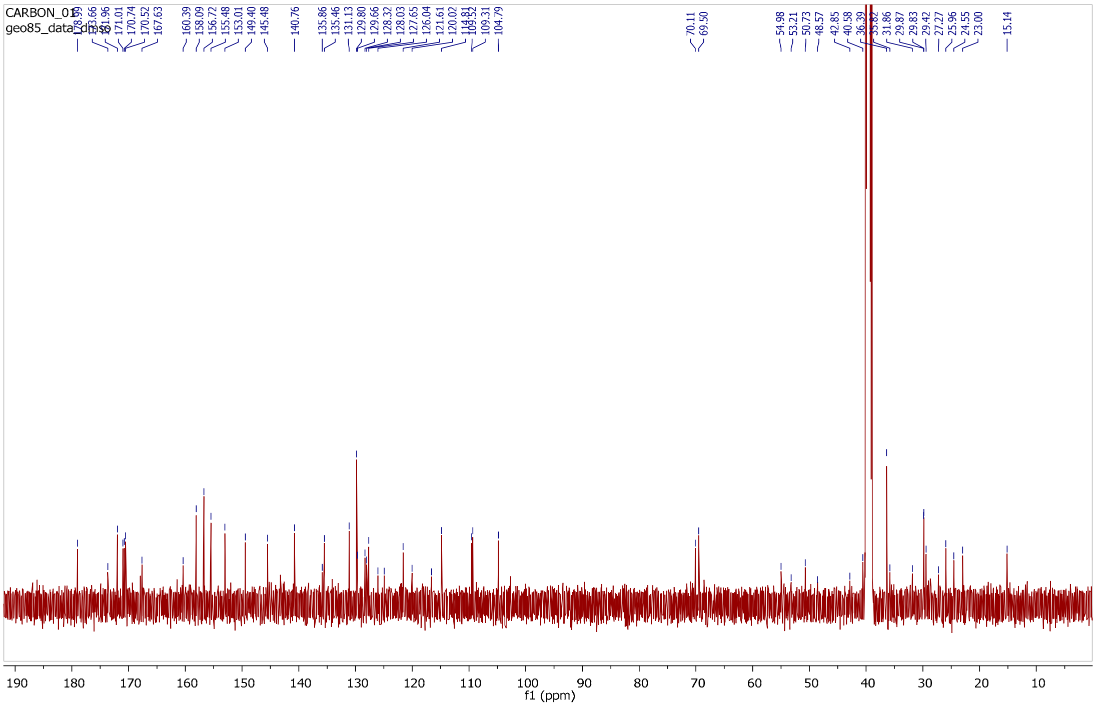

Figure S42. ^{1}H-NMR and ^{13}C-NMR spectra for compound **geo85**.

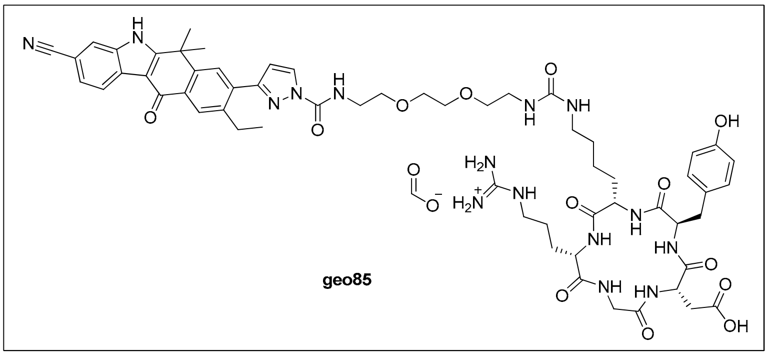

Method 1

Retention time: 20.9 m

area: 11577332 (absorbance units x minutes)

total area: 11811289 (absorbance units x minutes)

area %: (11577332/11811289)*100 = 98.0 %

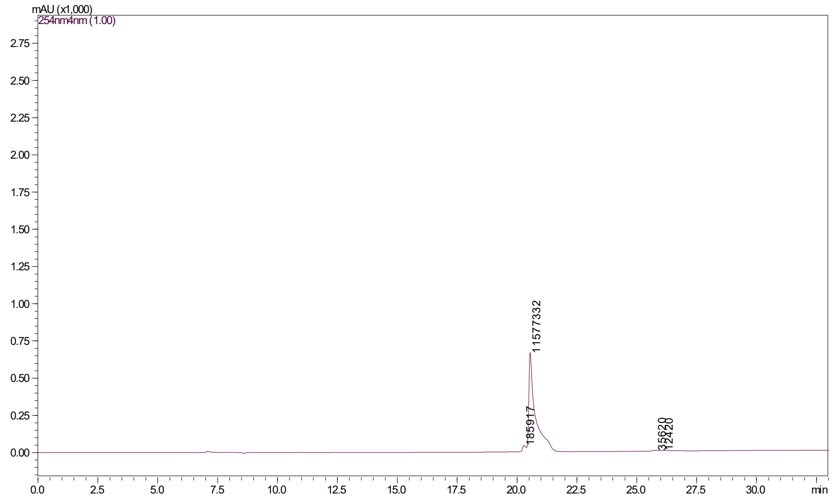

Figure S43. ESI-LCMS of compound **geo85** after HPLC purification

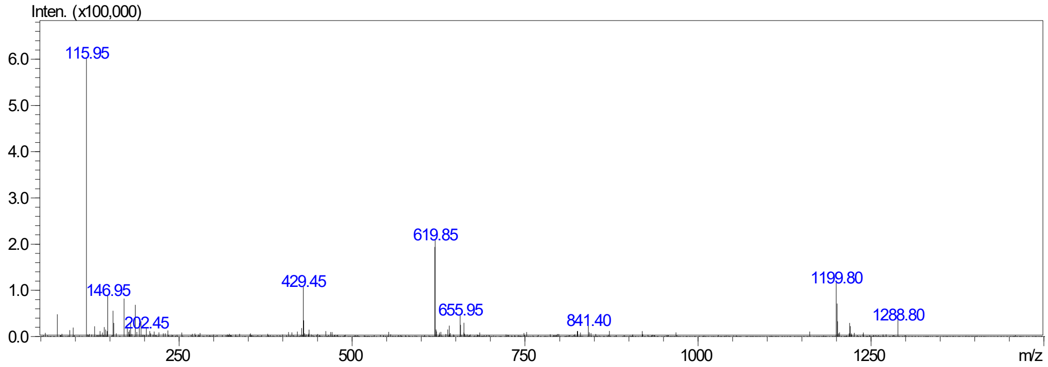

Figure S44. ESI-MS for **geo85**, positive mode: m/z calcd mass for $C_{59}H_{74}N_{15}O_{13}$ $[M+H]^{+}$=1200.55, was found 1199.80.

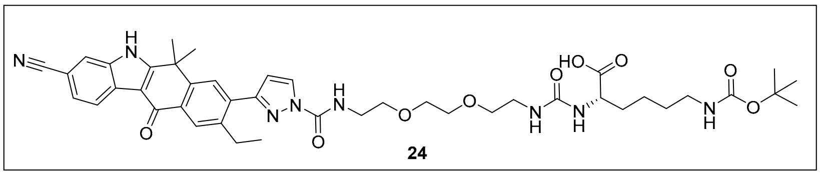

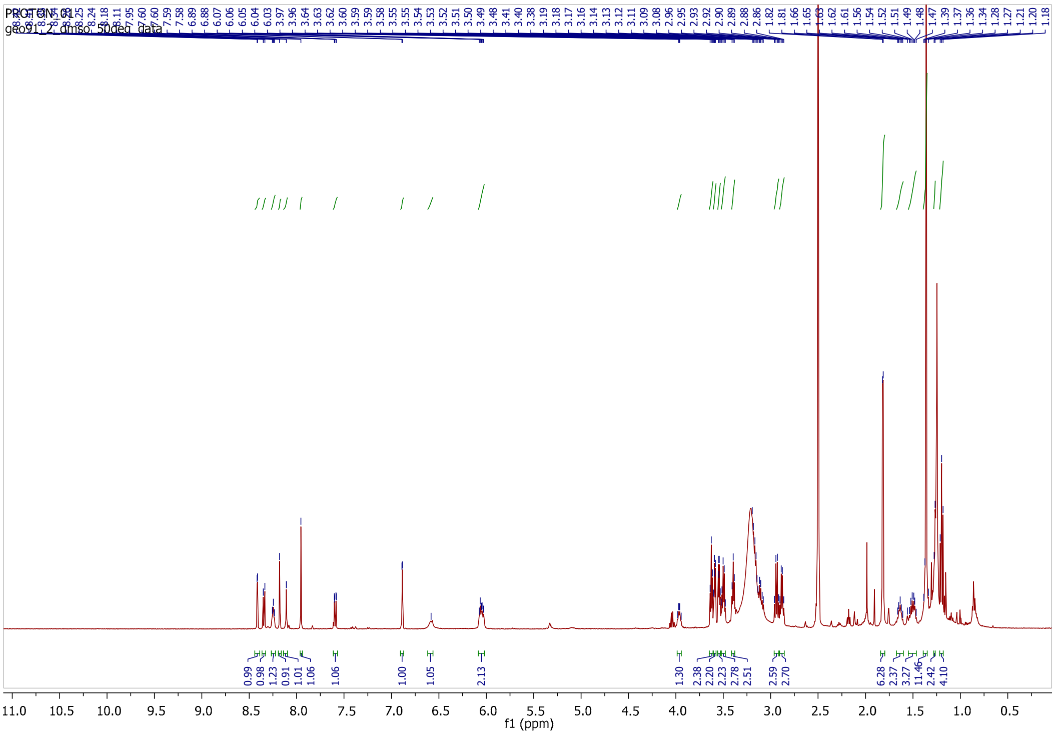

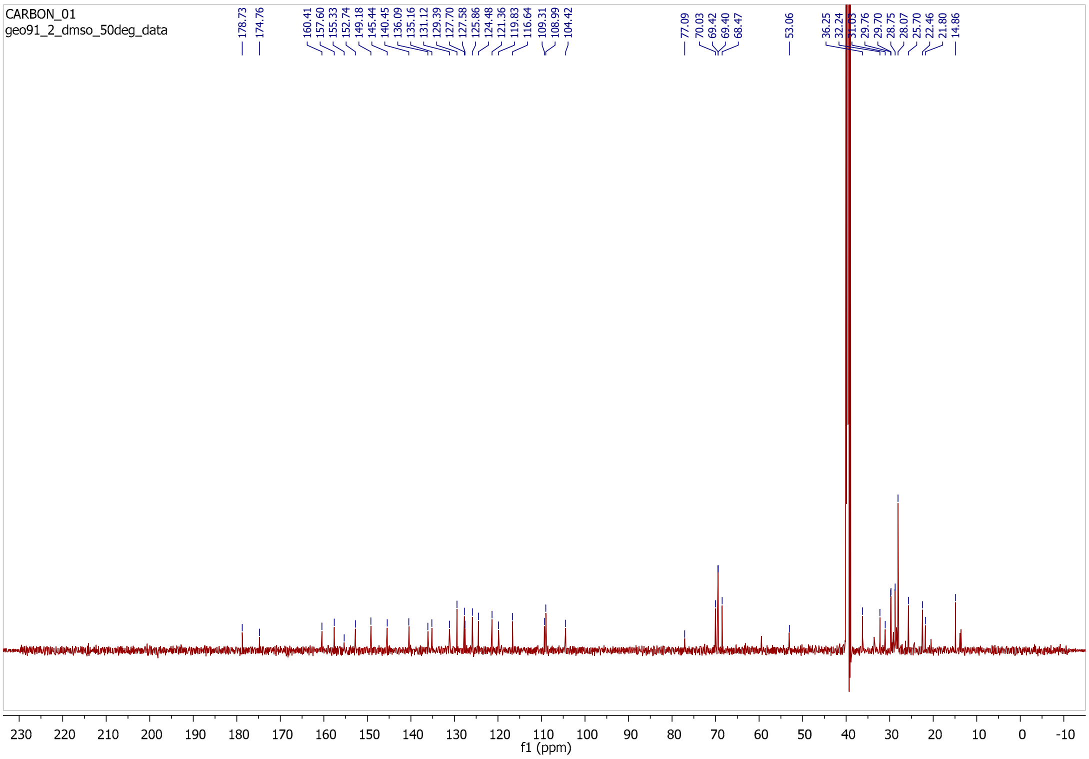

Figure S45. ^{1}H-NMR and ^{13}C-NMR spectra for compound **24**.

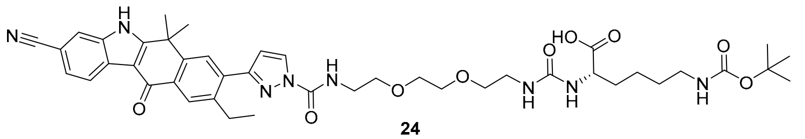

24

Method 1

Retention time: 27.4 m

area: 6702040 (absorbance units x minutes)

total area: 7142814 (absorbance units x minutes)

area %: (6702040/7142814)*100 = 93.8 %

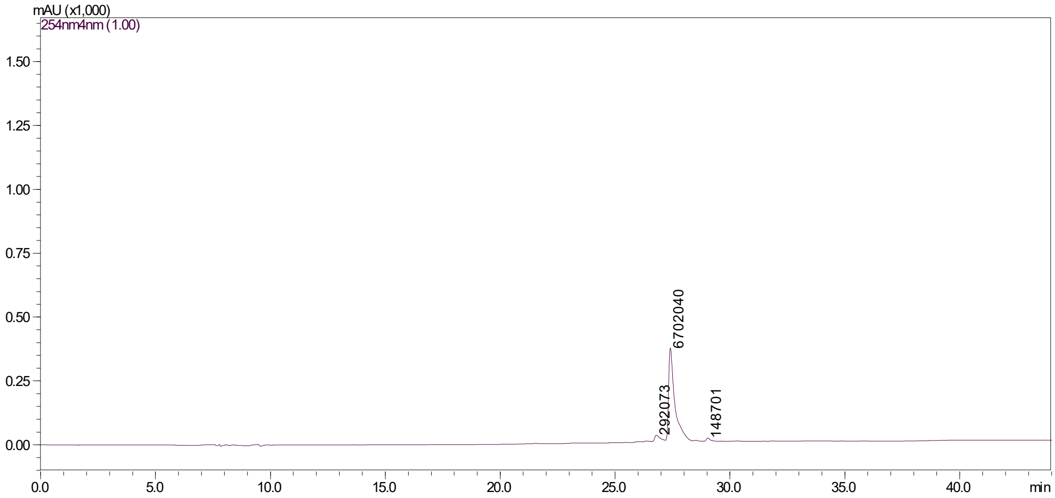

Figure S46. ESI-LCMS of compound **24** after column chromatography purification

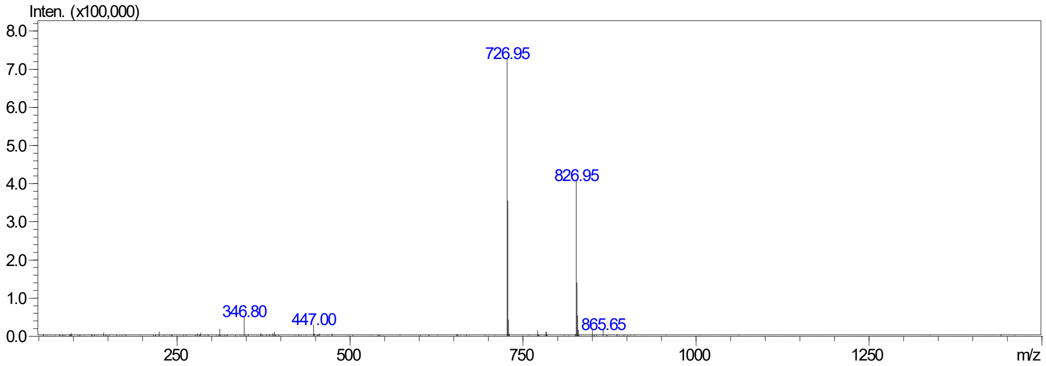

Figure S47. ESI-MS for **24**, positive mode: m/z calcd mass for $C_{43}H_{55}N_8O_9$ $[M+H]^+$=827.40, was found 826.95

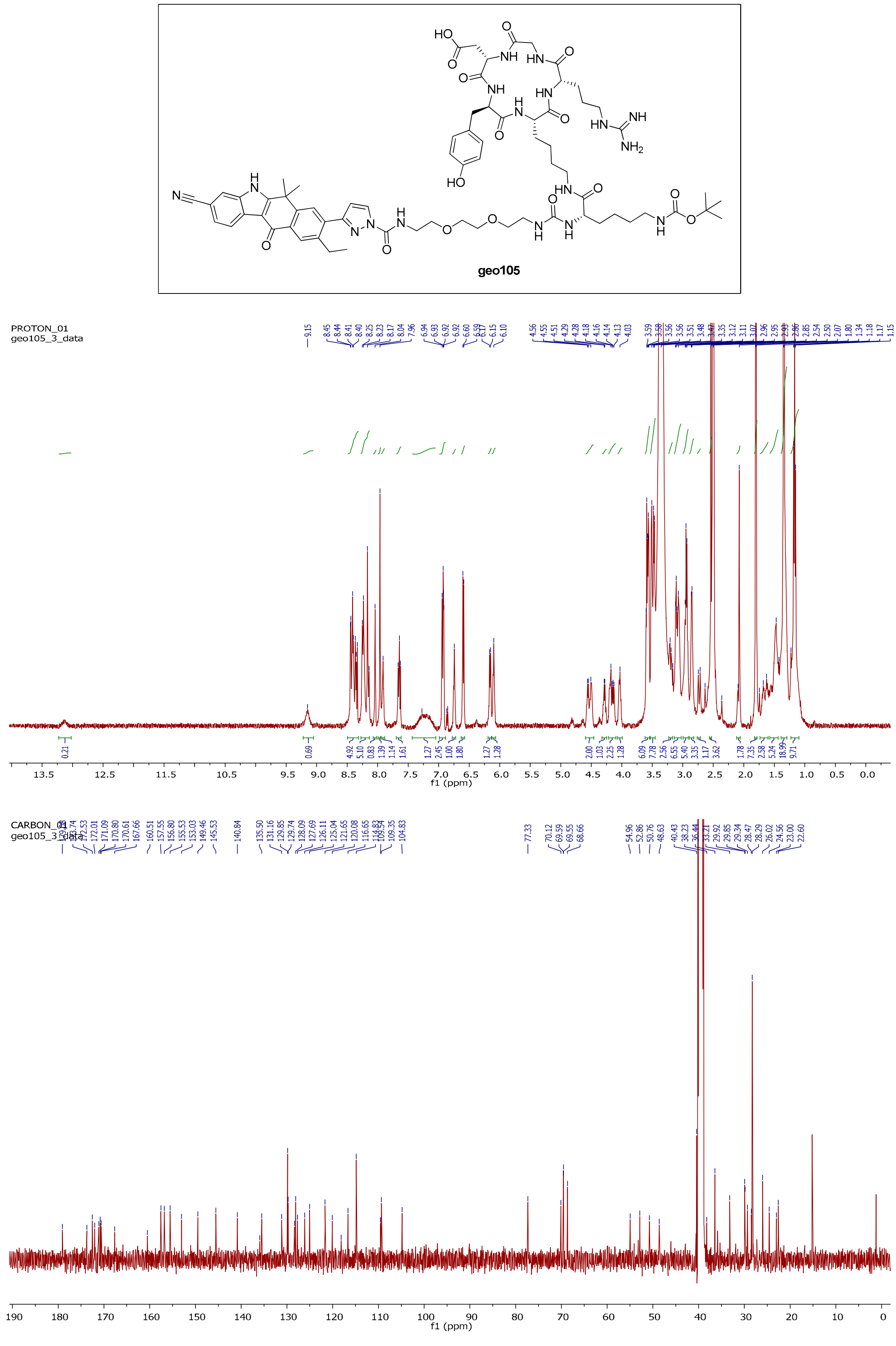

Figure S48. ^{1}H-NMR and ^{13}C-NMR spectra for compound **geo105**.

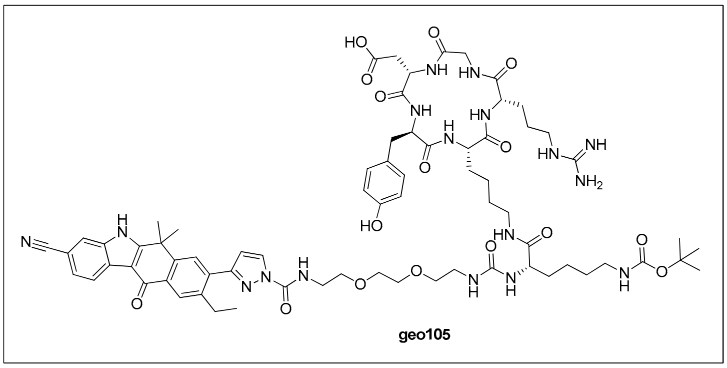

Method 1

Retention time: 21.8 m

area: 11056999 (absorbance units x minutes)

total area: 11122330 (absorbance units x minutes)

area %: (11056999/11122330)*100 = 99.4 %

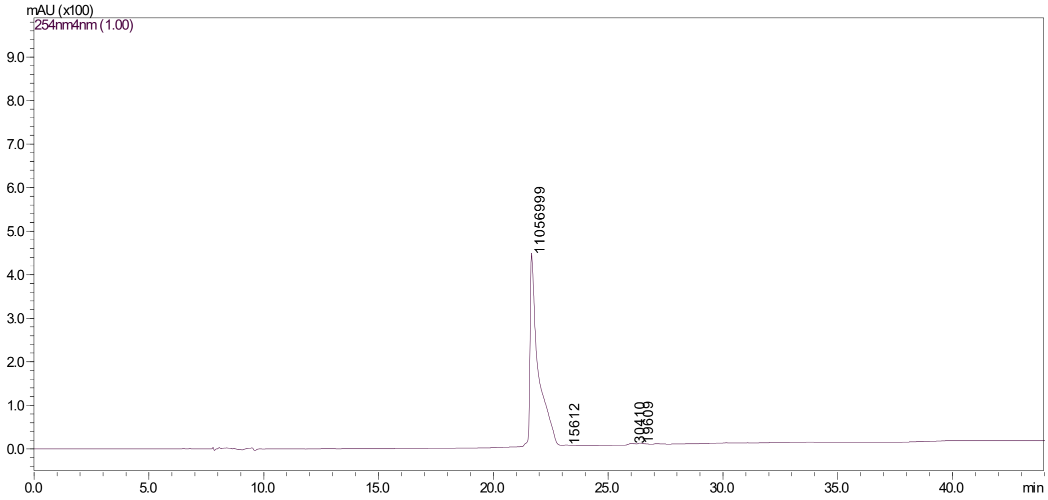

Figure S49. ESI-LCMS of compound **geo105** after column chromatography purification

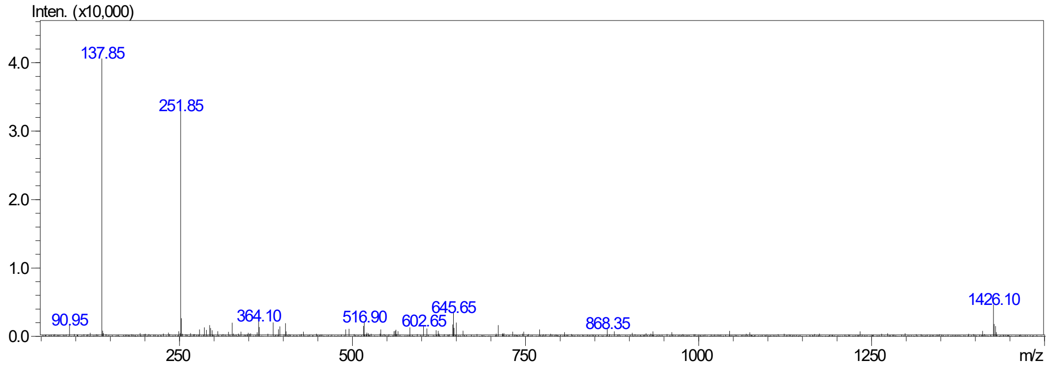

Figure S50. ESI-MS for **geo105**, negative mode: m/z calcd mass for $C_{70}H_{93}N_{17}O_{16}$ $[M-H]^-$ =1426.70, was found 1426.10.

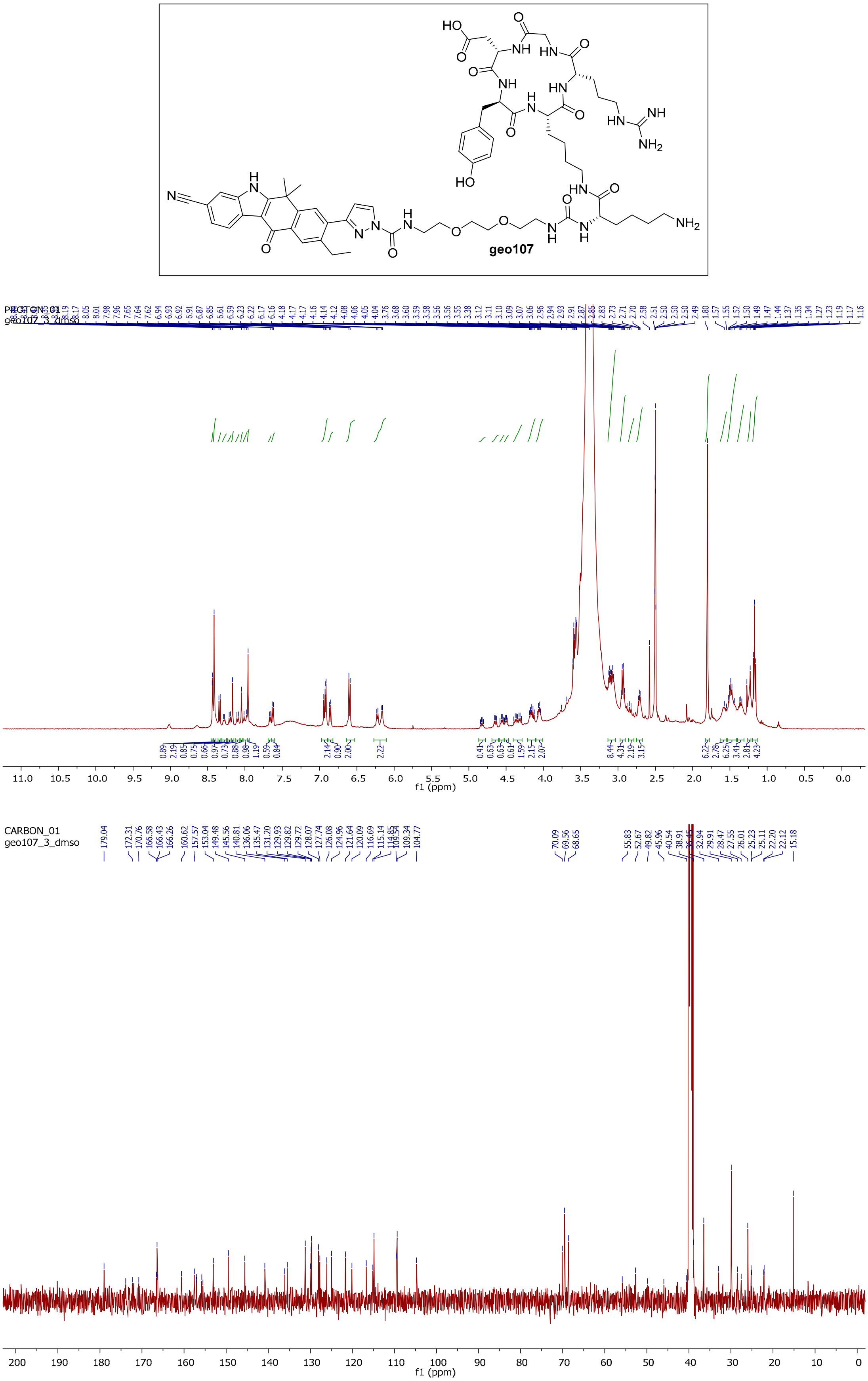

Figure S51. ^{1}H-NMR and ^{13}C-NMR spectra for compound **geo107**.

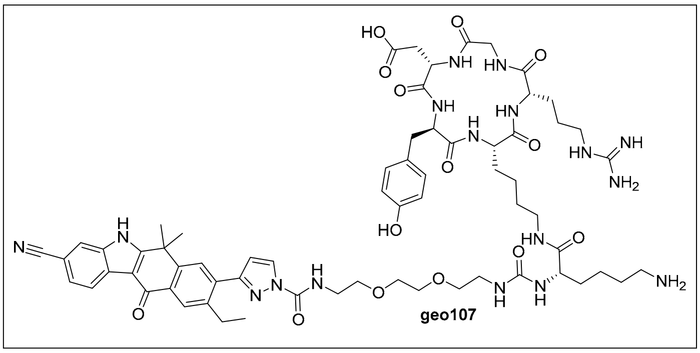

Method 1

Retention time: 18.8 m

area: 1819194 (absorbance units x minutes)

total area: 1855459 (absorbance units x minutes)

area %: (1819194/1855459)*100 = 98.0 %

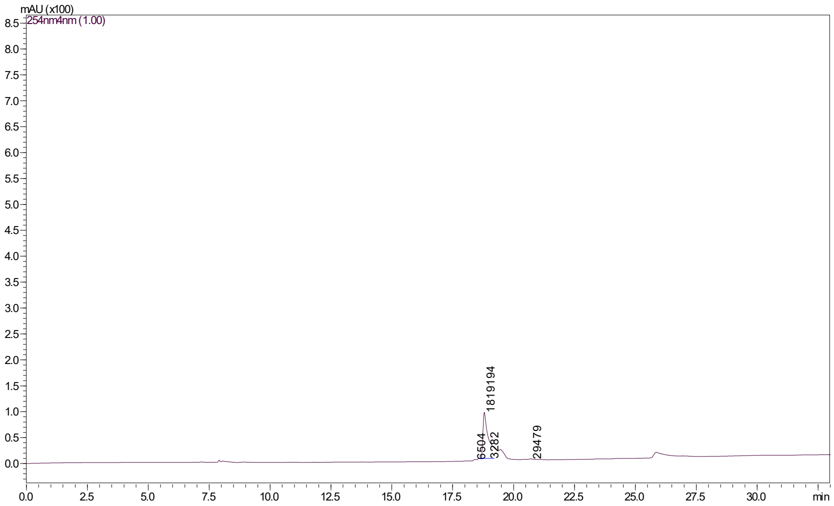

Figure S52. ESI-LCMS of compound **geo107** after HPLC purification

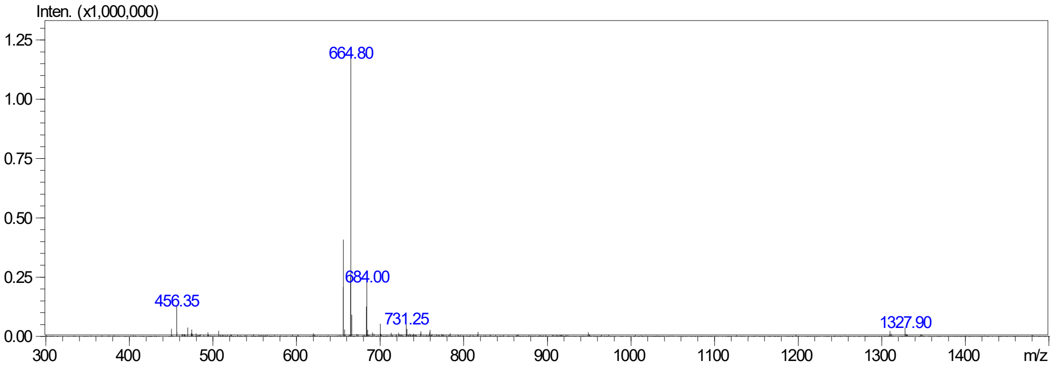

Figure S53. ESI-MS for **geo107**, positive mode: m/z calcd mass for $C_{65}H_{86}N_{17}O_{14}$ $[M+H]^+$= 1328.65, was found 1327.9.

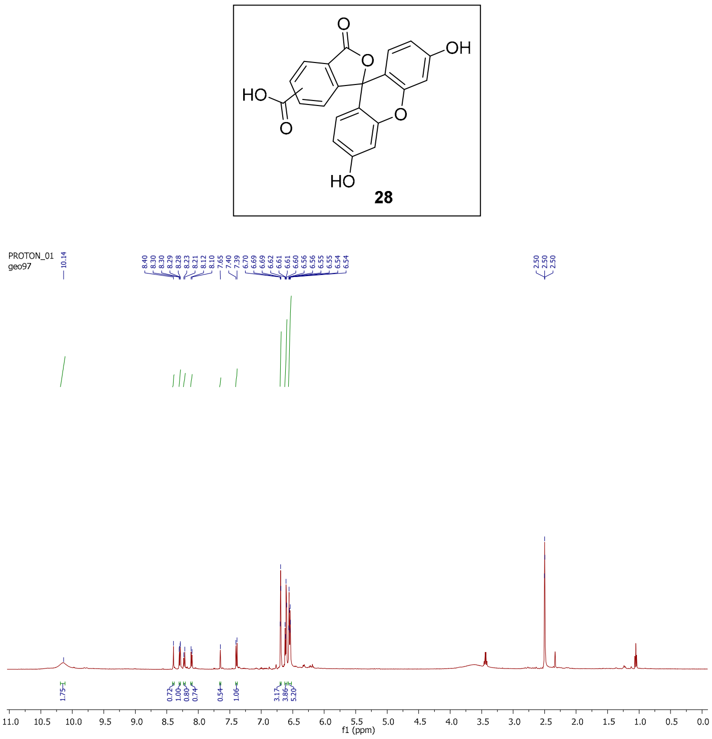

Figure S54. ^{1}H-NMR spectrum for compound **28**.

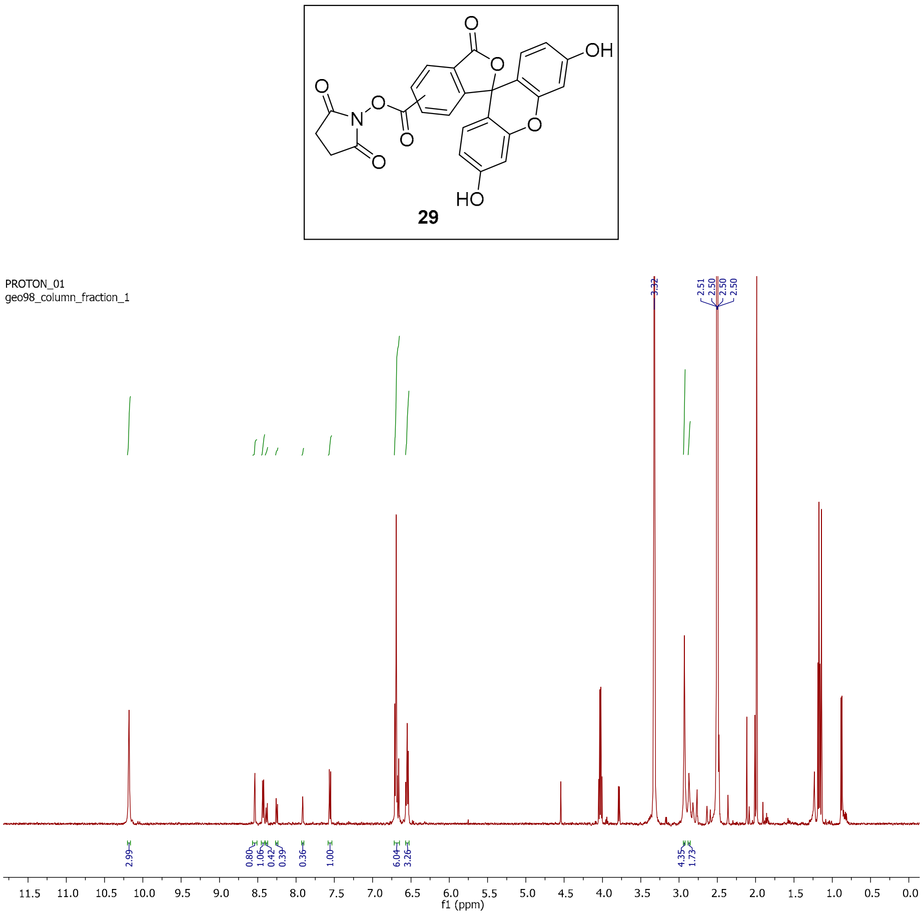

Figure S55. ^{1}H-NMR spectrum for compound **29**.

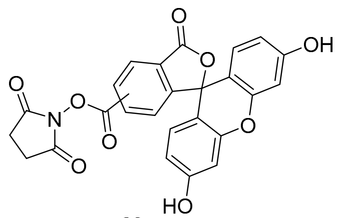

29

Method 1

Retention time: 22.6 m

area: 19307526 (absorbance units x minutes)

total area: 20715865 (absorbance units x minutes)

area %: (19307526/20715865)*100 = 93.2 %

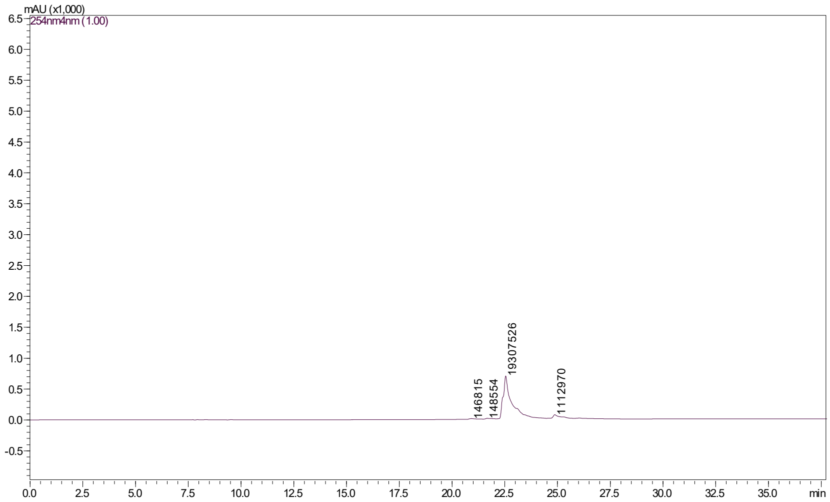

Figure S56. ESI-LCMS of compound **29** after column chromatography purification

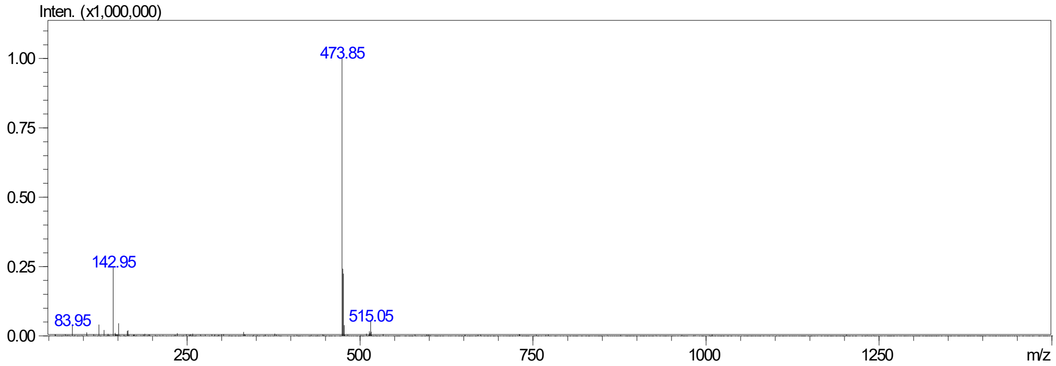

Figure S57. ESI-MS for **29**, positive mode: m/z calcd mass for $C_{25}H_{15}NO_9$ $[M]^+$=473.07, was found 473.85.

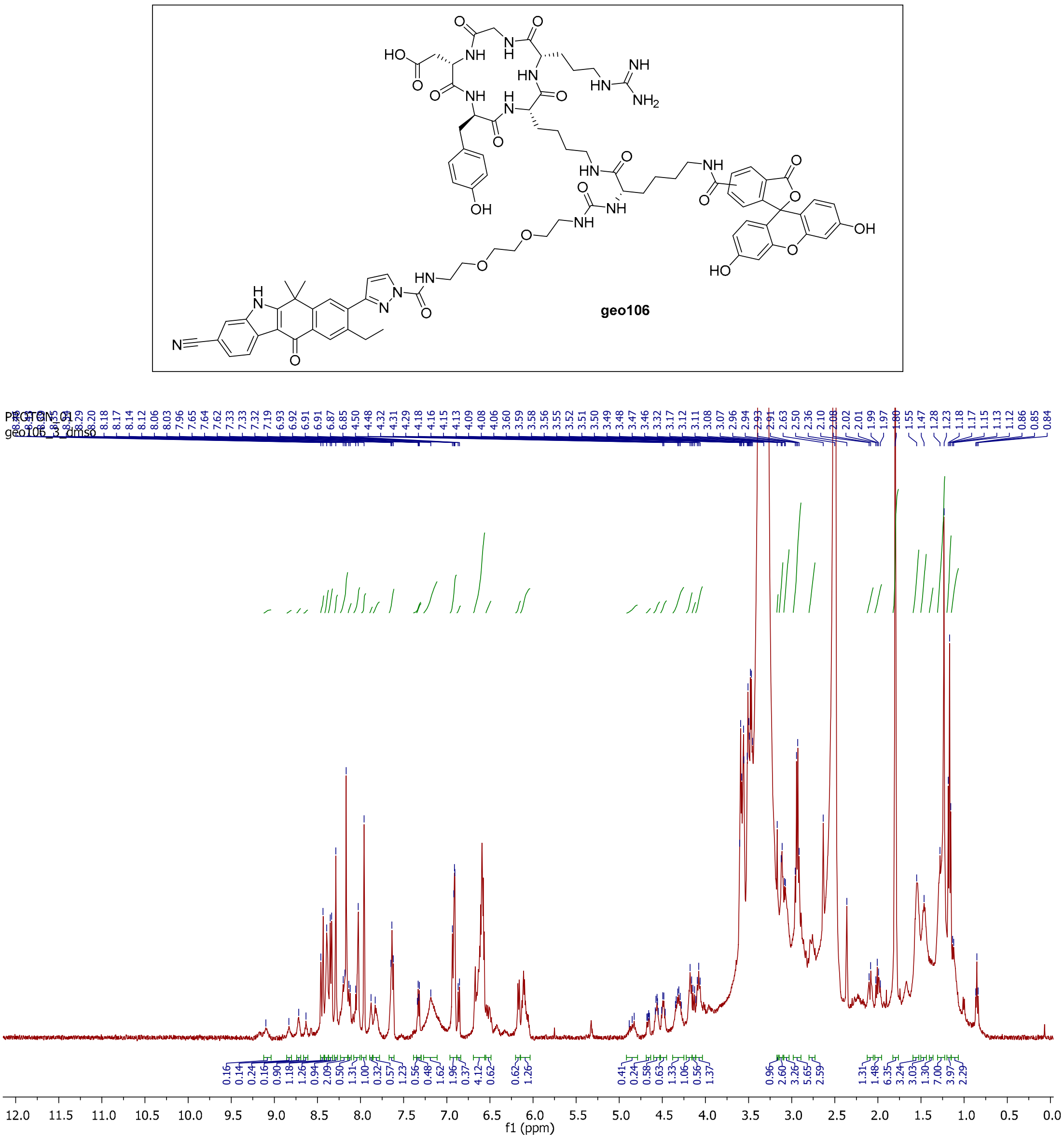

Figure S58. ^{1}H-NMR spectrum for compound **geo106**.

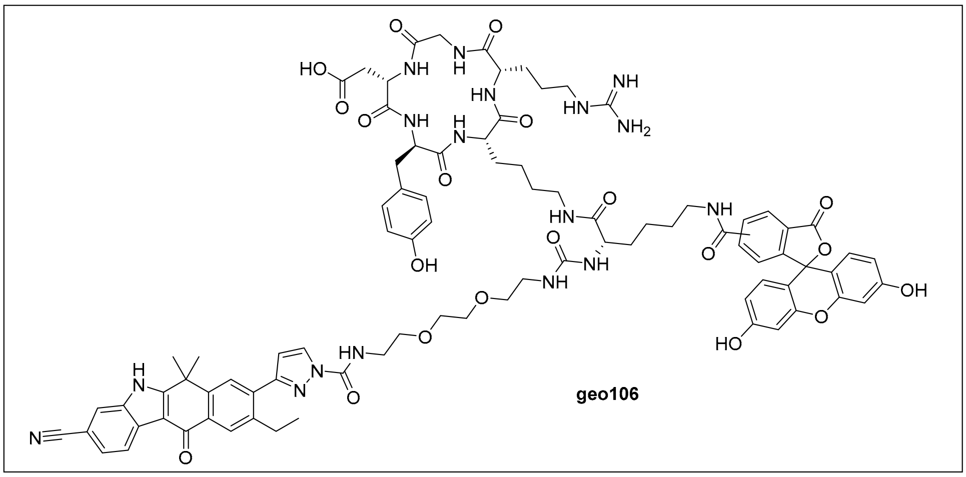

Method 5

Retention time: 21.3 m

area: 12951299 (absorbance units x minutes)

total area: 13466339 (absorbance units x minutes)

area %: (12951299/13466339)*100 = 96.1 %

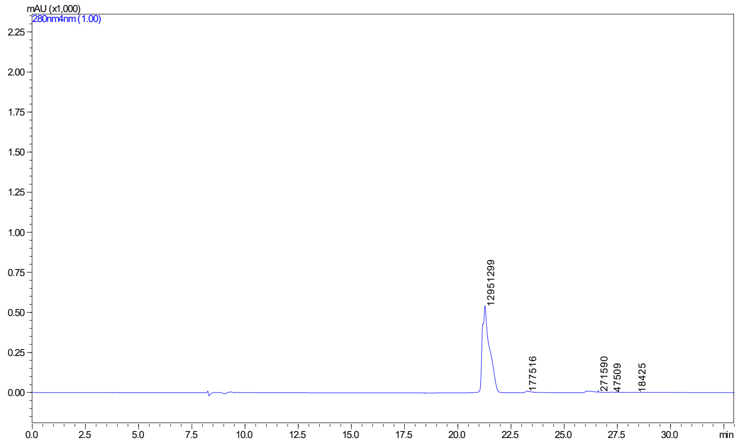

Figure S59. ESI-LCMS of compound **geo106** after HPLC purification

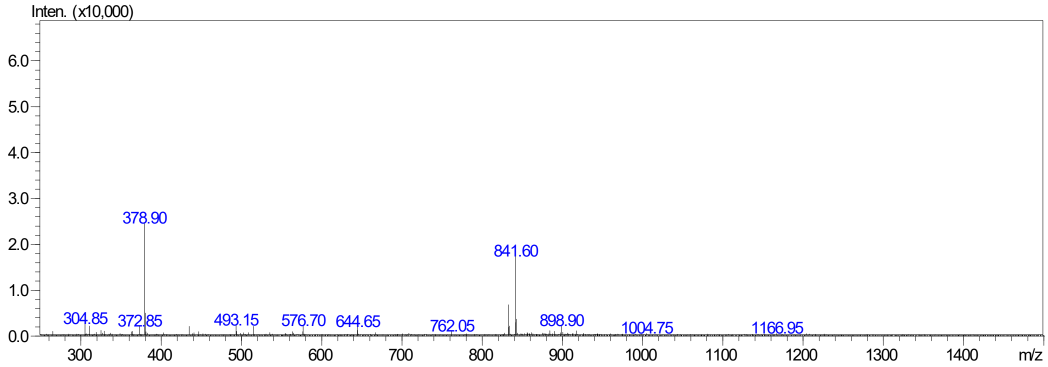

Figure S60. ESI-MS for **geo106**, negative mode: m/z calcd mass for $C_{86}H_{95}N_{17}O_{20}$ $[M/2-H]^{-}$= 841.84, was found 841.60.

Chemostability Studies

Compound **geo75**

- pH = 5.2, method 4

t = 0

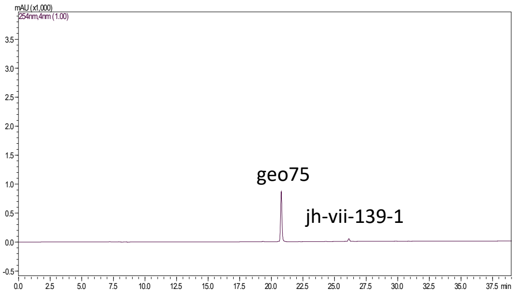

t = 5 h

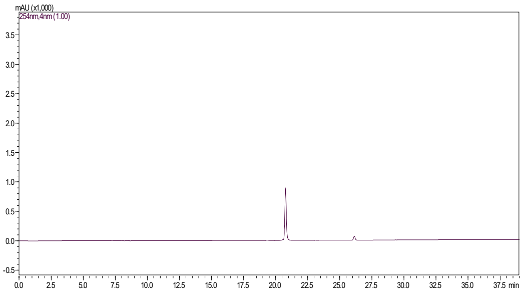

t = 24 h

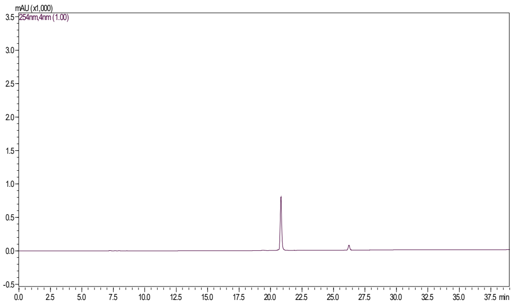

t = 48 h

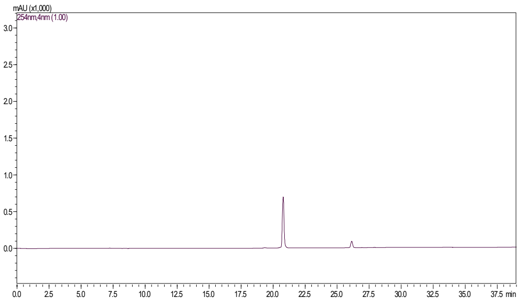

Figure S61. Stability of compound **geo75** at pH=5.2.

- pH = 7.4, method 4

t = 0

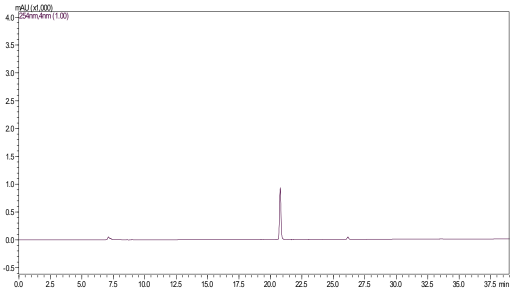

t = 5 h

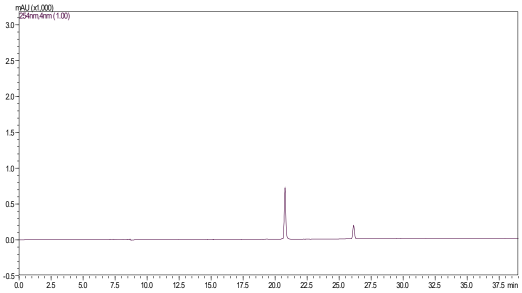

t = 24 h

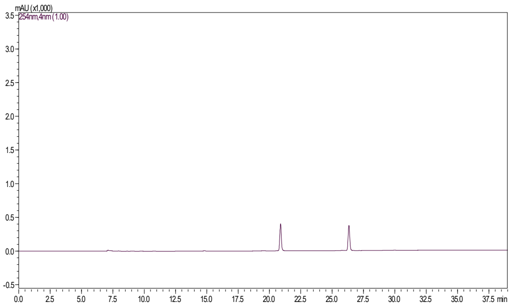

t = 48 h

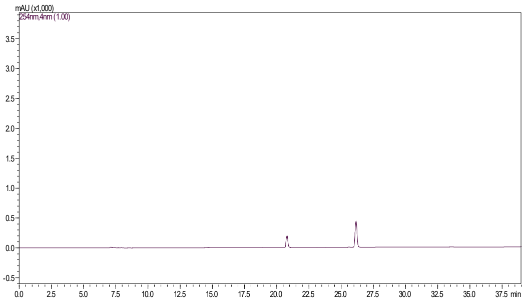

Figure S62. Stability of compound **geo75** at pH=7.4.

- DMEM, method 4

t = 0

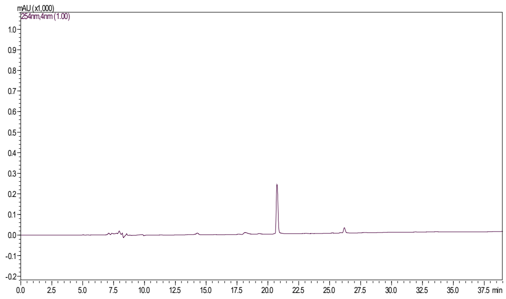

t = 3 h

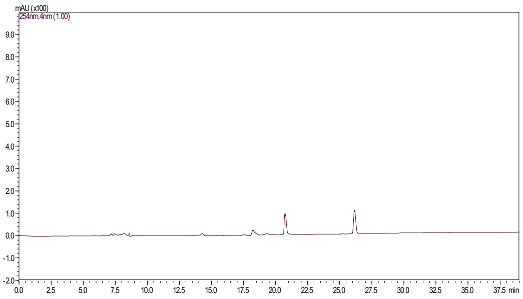

t = 6 h

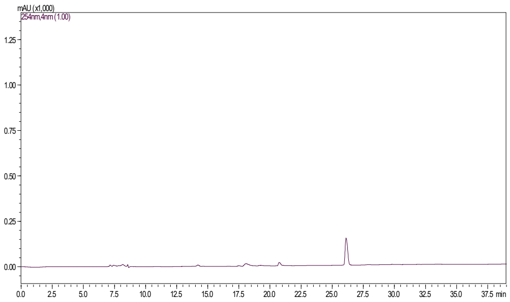

t = 9 h

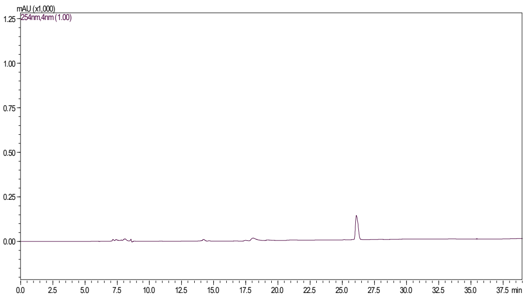

Figure S63. Stability of compound **geo75** in DMEM.

- Human Plasma, method 2

t = 0 h

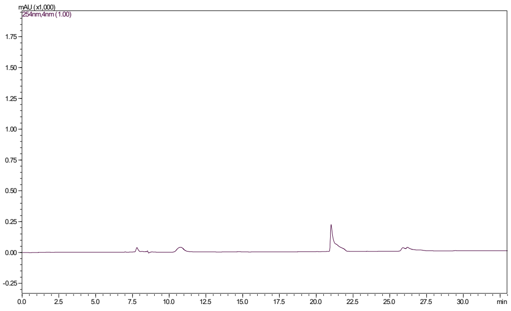

t = 1 h

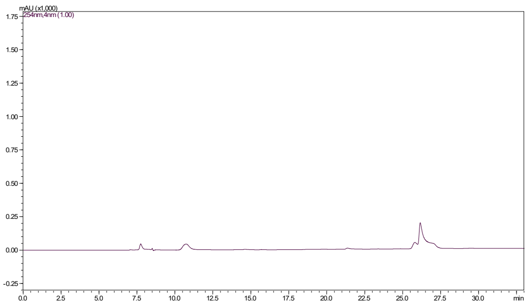

t = 2 h

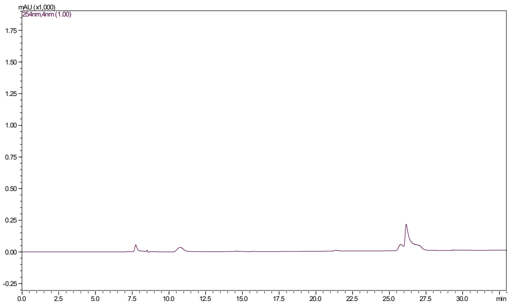

t = 24 h

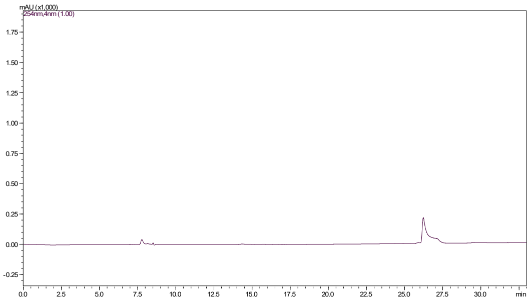

Figure S64. Stability of compound **geo75** in human plasma.

Compound **geo77**

➢ pH = 5.2, method 2

t = 0 h

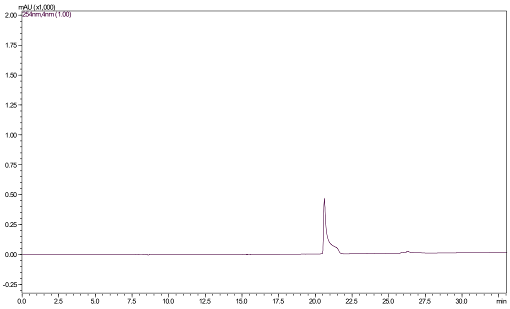

t = 5 h

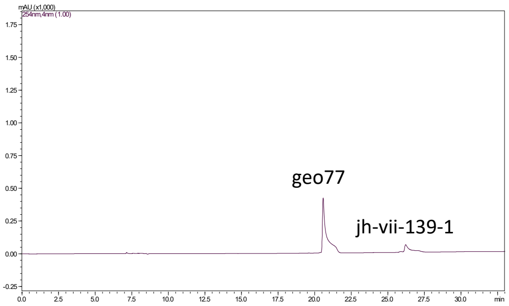

t = 24 h

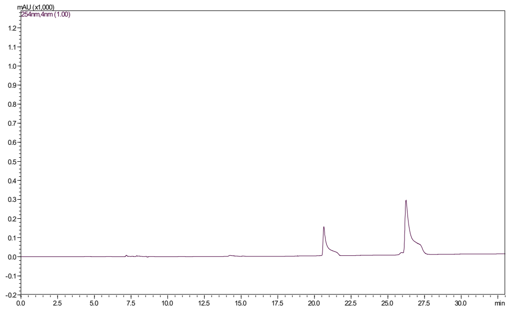

t = 48 h

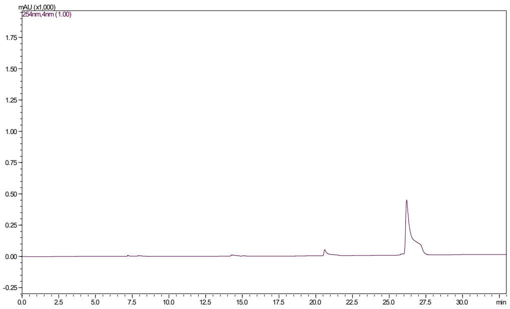

Figure S65. Stability of compound **geo77** at pH=5.2.

➢ pH = 7.4, method 2

t = 0 h

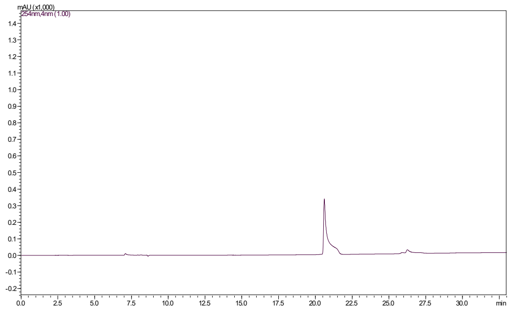

t = 2 h

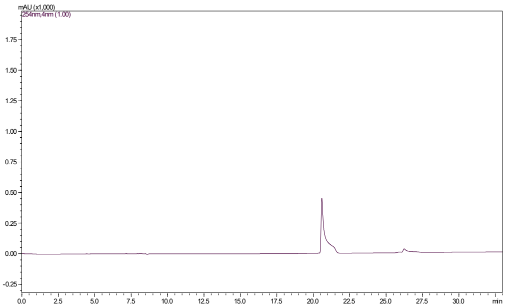

t = 5 h

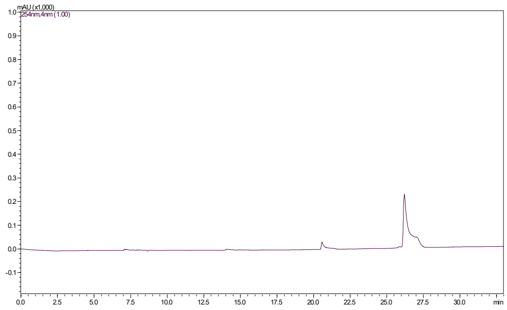

t = 24 h

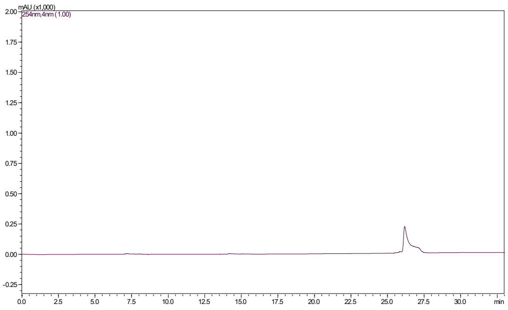

Figure S66. Stability of compound **geo77** at pH=7.4.

- DMEM, method 4

t = 0 h

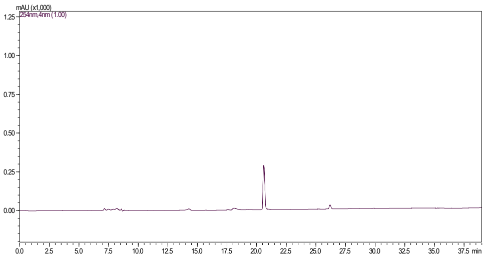

t = 1.5 h

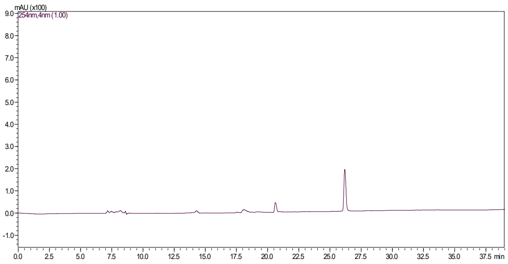

t = 3 h

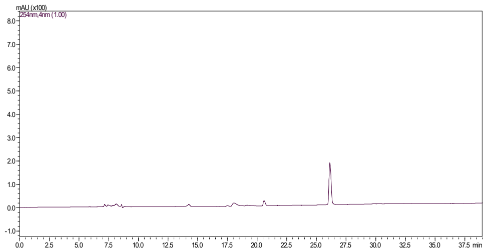

t = 7.5 h

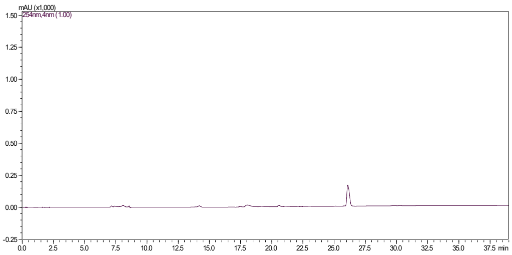

Figure S67. Stability of compound **geo77** in DMEM.

- Human Plasma, method 2

t = 0 h

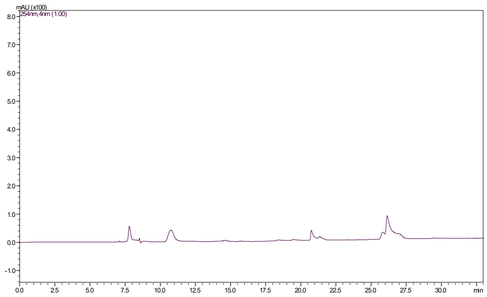

t = 1 h

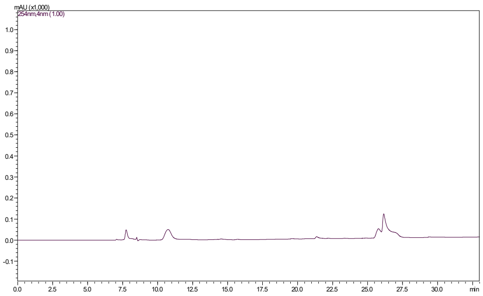

t = 2 h

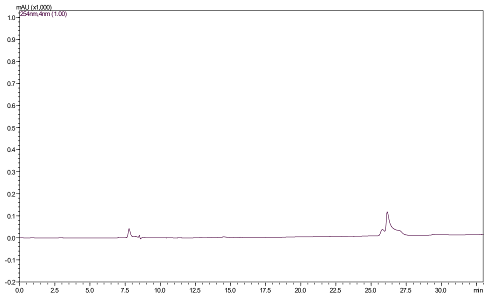

t = 24 h

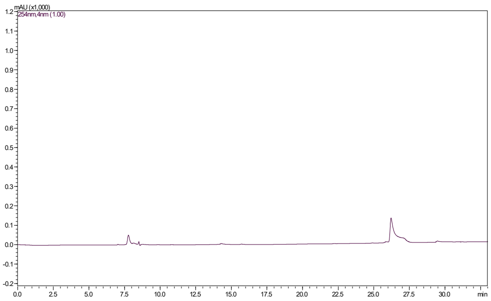

Figure S68. Stability of compound **geo77** in human plasma.

Compound **geo85**

- pH = 5.2, method 2

t = 0 h

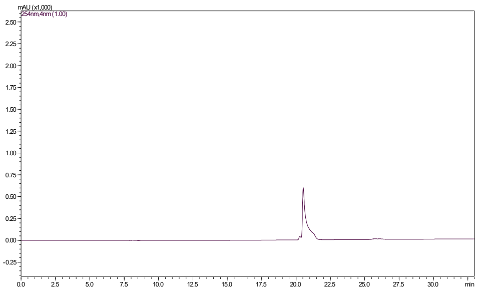

t = 3 h

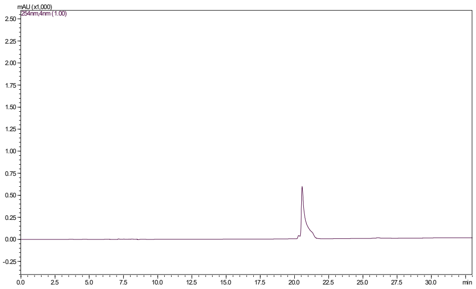

t = 5 h

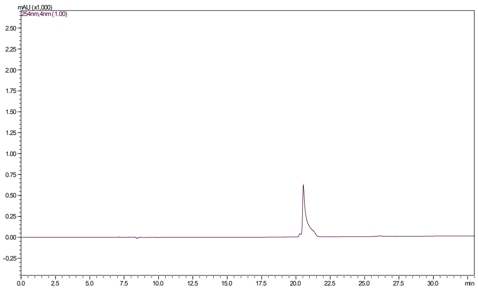

t = 48 h

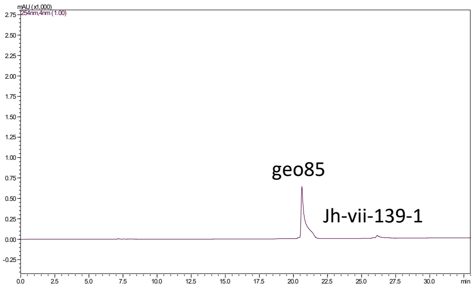

Figure S69. Stability of compound **geo85** at pH=5.2.

- pH = 7.4, method 2

t = 0 h

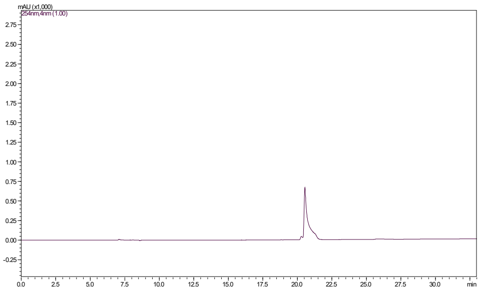

t = 3 h

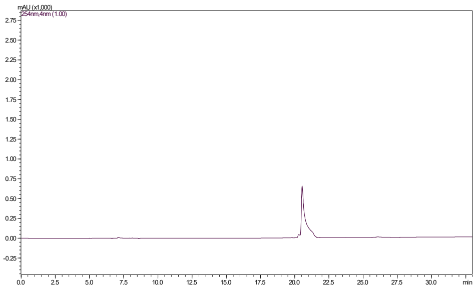

t = 5 h

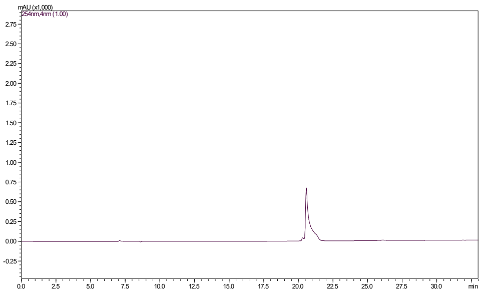

t = 48 h

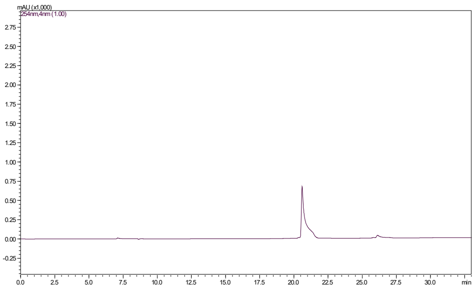

Figure S70. Stability of compound **geo85** at pH=7.4.

- DMEM, method 2

t = 0 h

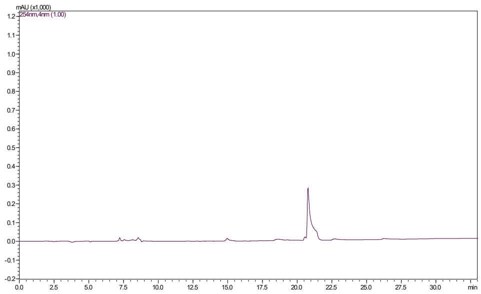

t = 3 h

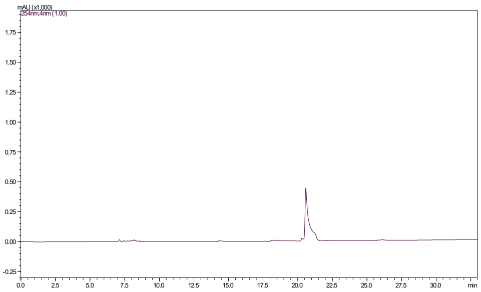

t = 24 h

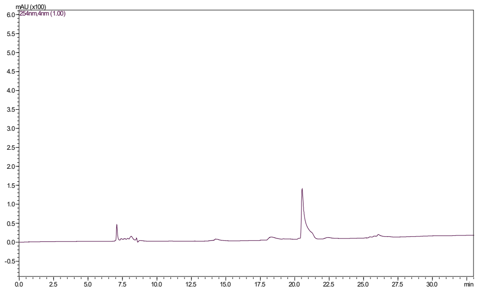

t = 48 h

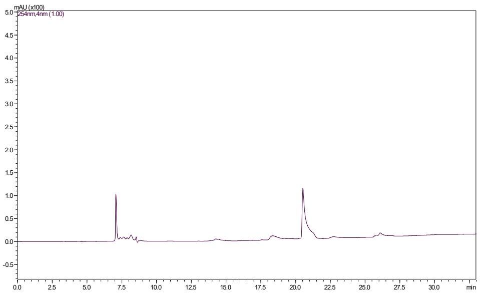

Figure S71. Stability of compound **geo85** in DMEM.

- Human Plasma, method 1

t = 0 h

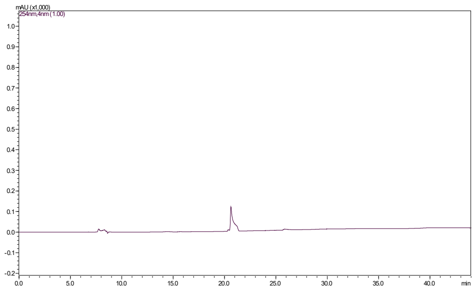

t = 3 h

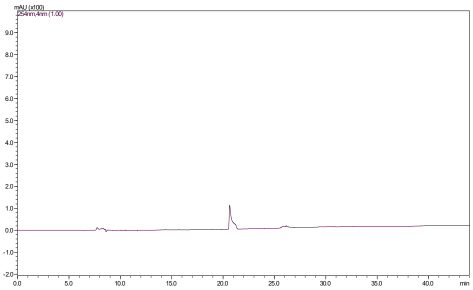

t = 5 h

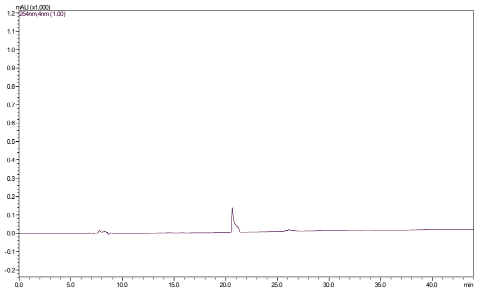

t = 48 h

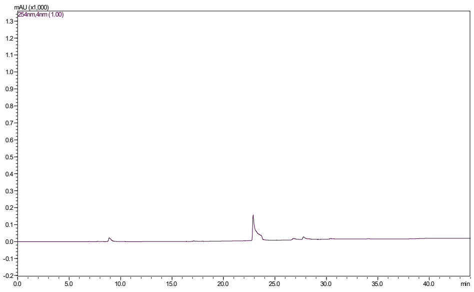

Figure S72. Stability of compound **geo85** in human plasma.

Compound **geo107**

- pH = 5.2, method 2

t = 0 h

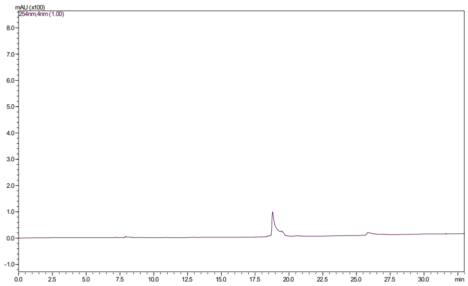

t = 3 h

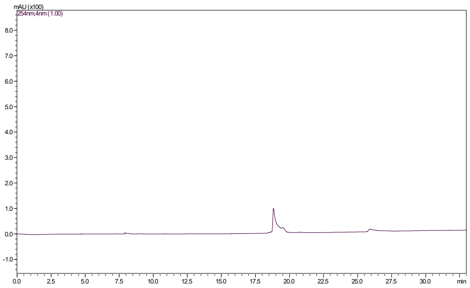

t = 5 h

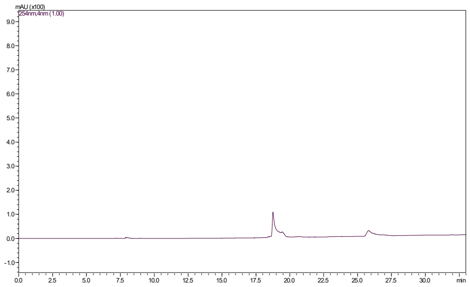

t = 24 h

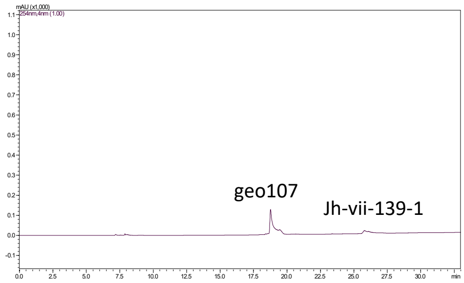

Figure S73. Stability of compound **geo107** at pH=5.2.

Compound **geo107**

- pH = 7.4, method 2

t = 0 h

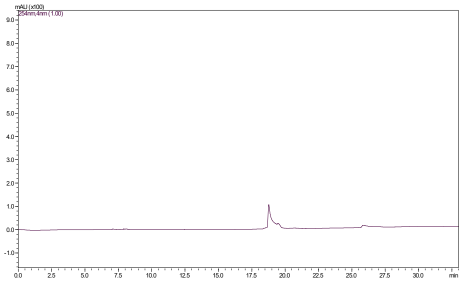

t = 3 h

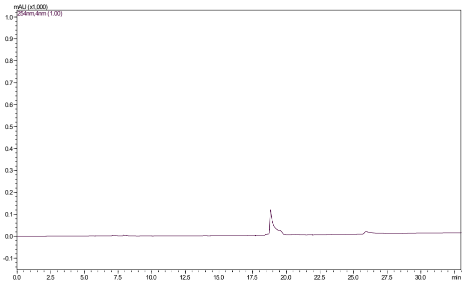

t = 5 h

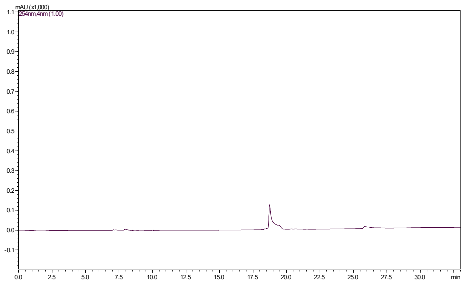

t = 24 h

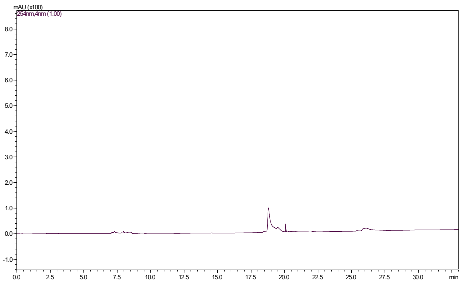

Figure S74. Stability of compound **geo107** at pH=7.4.

Compound **geo107**

- DMEM, method 2

t = 0 h

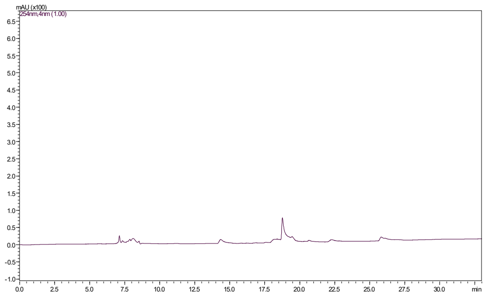

t = 2 h

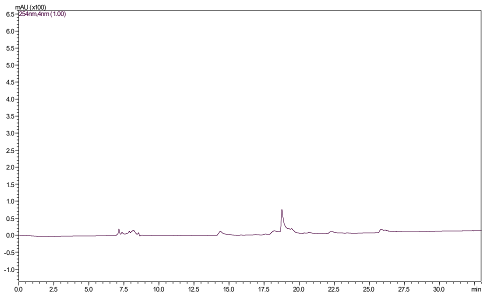

t = 5 h

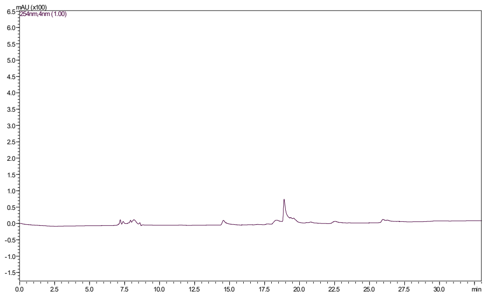

t = 48 h

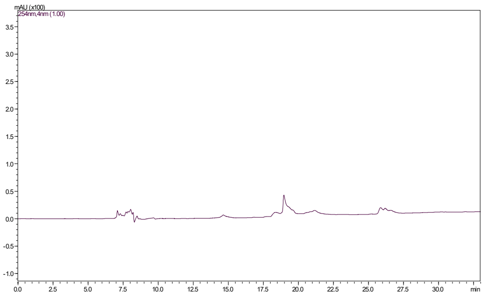

Figure S75. Stability of compound **geo107** in DMEM.

Compound **geo107**

- Human Plasma, method 2

t = 0 h

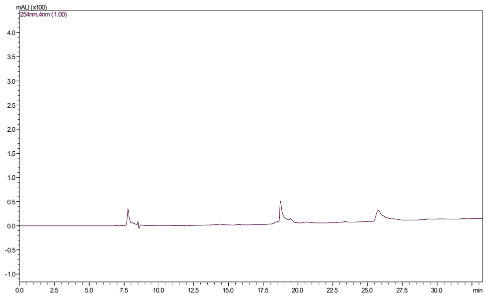

t = 2 h

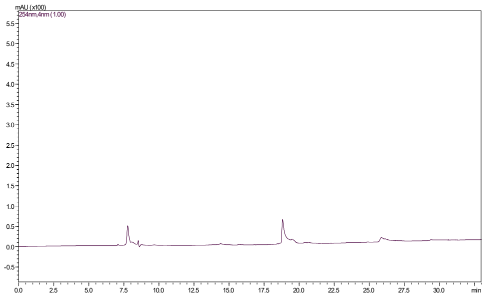

t = 5 h

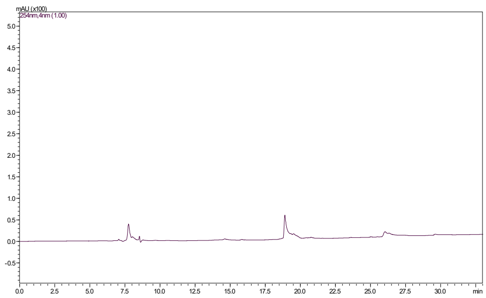

t = 48 h

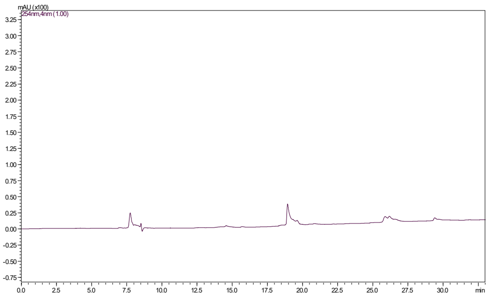

Figure S76. Stability of compound **geo107** in human plasma.

Compound **JH-VII-139-1**

➢ DMEM, method 1

t = 0 h

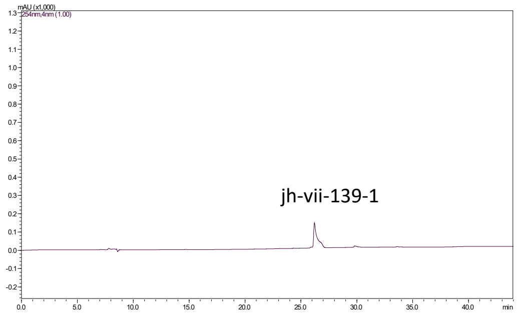

t = 2 h

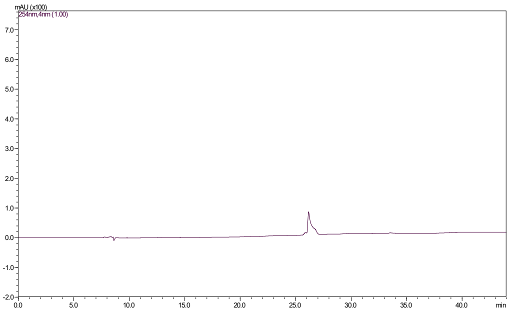

t = 24 h

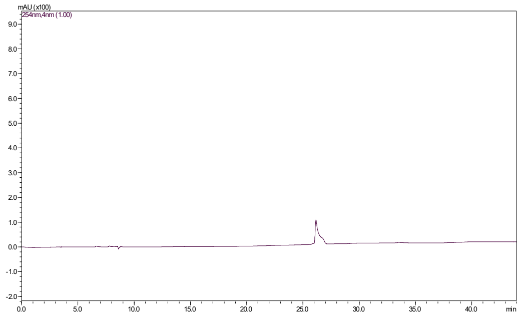

t = 48 h

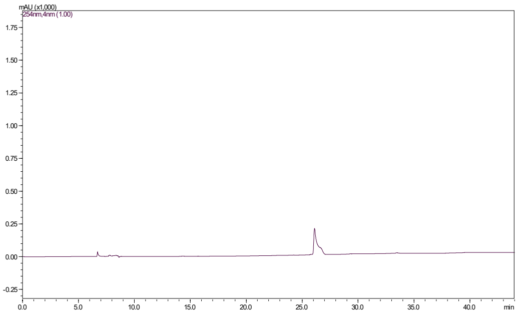

Figure S77. Stability of compound **JH-VII-139-1** in DMEM.

➢ Human Plasma, method 2

t = 0 h

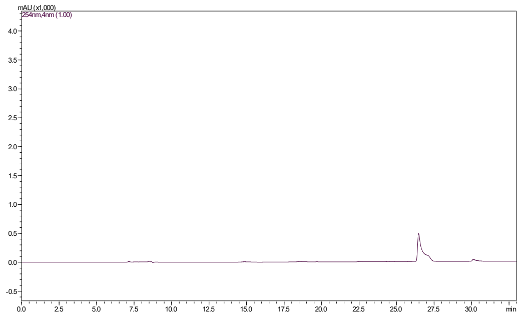

t = 3 h

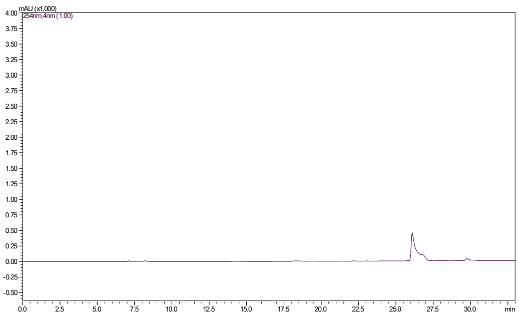

t = 5 h

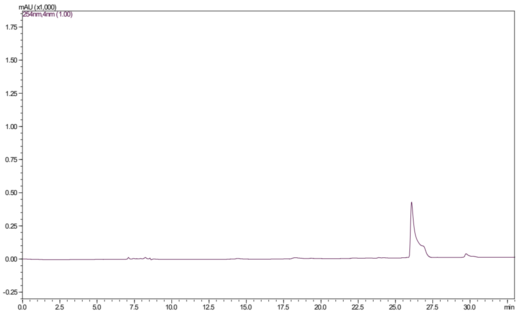

t = 48 h

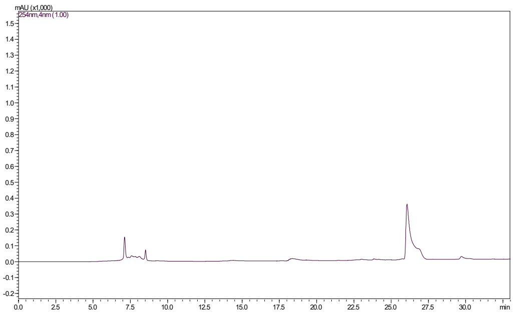

Figure S78. Stability of compound **JH-VII-139-1** in human plasma.

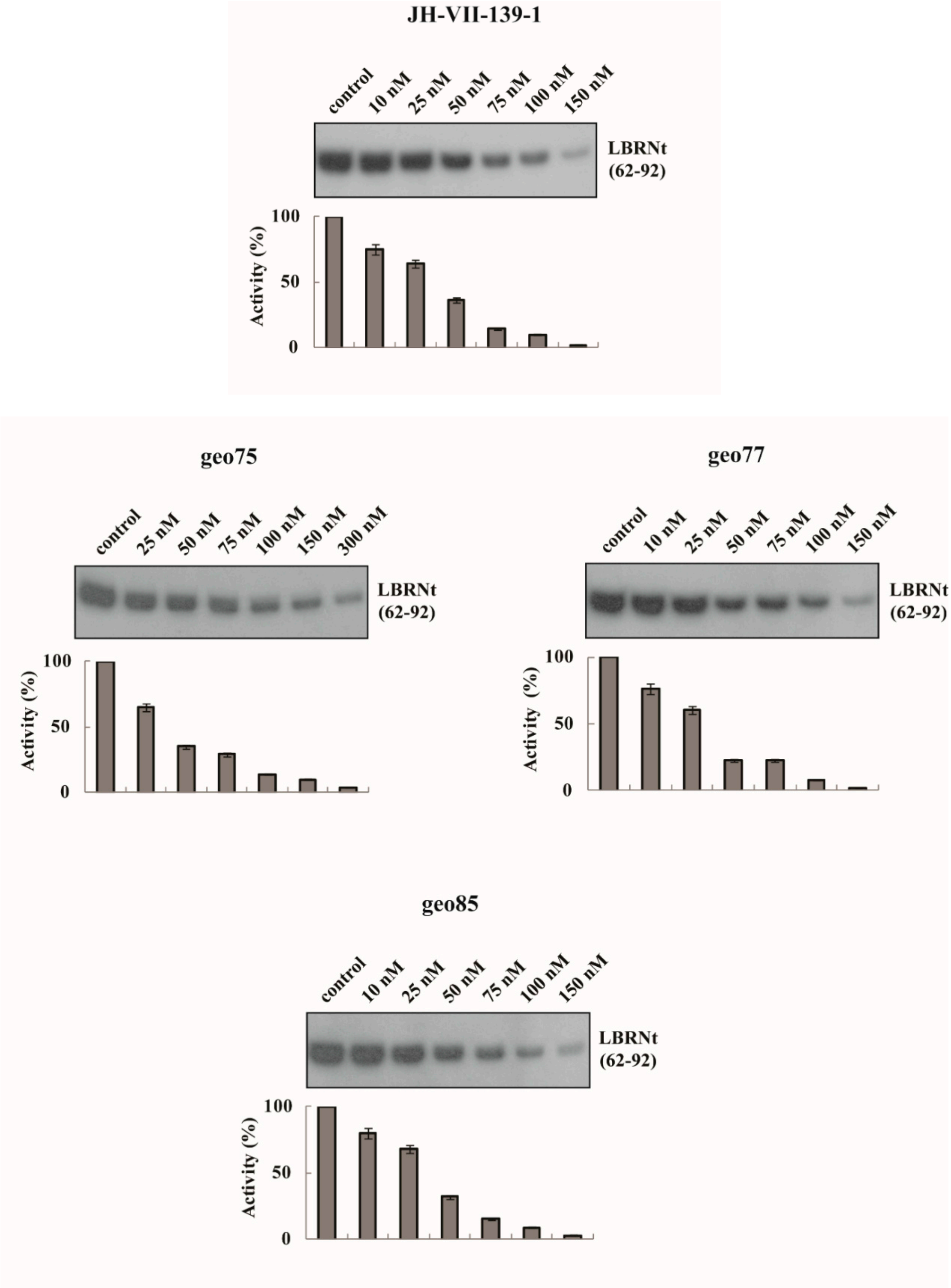

Figure S79. In vitro phosphorylation of GST-LBRNt(62-92) by GST-SRPK1 in the presence of increasing concentrations (10-300 nM) of JH-VII-139-1, **geo75**, **geo77** and **geo85** respectively. Phosphorylated bands were excised from the dry gel and Cherenkof counted.
